# THEM6‐mediated reprogramming of lipid metabolism supports treatment resistance in prostate cancer

**DOI:** 10.15252/emmm.202114764

**Published:** 2022-01-11

**Authors:** Arnaud Blomme, Coralie Peter, Ernest Mui, Giovanny Rodriguez Blanco, Ning An, Louise M Mason, Lauren E Jamieson, Grace H McGregor, Sergio Lilla, Chara Ntala, Rachana Patel, Marc Thiry, Sonia H Y Kung, Marine Leclercq, Catriona A Ford, Linda K Rushworth, David J McGarry, Susan Mason, Peter Repiscak, Colin Nixon, Mark J Salji, Elke Markert, Gillian M MacKay, Jurre J Kamphorst, Duncan Graham, Karen Faulds, Ladan Fazli, Martin E Gleave, Edward Avezov, Joanne Edwards, Huabing Yin, David Sumpton, Karen Blyth, Pierre Close, Daniel J Murphy, Sara Zanivan, Hing Y Leung

**Affiliations:** ^1^ CRUK Beatson Institute Garscube Estate Glasgow UK; ^2^ Institute of Cancer Sciences University of Glasgow Garscube Estate Glasgow UK; ^3^ Laboratory of Cancer Signaling GIGA‐Institute University of Liège Liège Belgium; ^4^ School of Engineering University of Glasgow Glasgow UK; ^5^ Centre for Molecular Nanometrology Department of Pure and Applied Chemistry Technology and Innovation Centre University of Strathclyde Glasgow UK; ^6^ GIGA‐Neurosciences Unit of Cell and Tissue Biology University of Liège Liège Belgium; ^7^ Department of Urologic Sciences Faculty of Medicine University of British Columbia Vancouver BC Canada; ^8^ Vancouver Prostate Centre Vancouver BC Canada; ^9^ UK Dementia Research Institute at University of Cambridge Department of Clinical Neurosciences University of Cambridge Cambridge UK

**Keywords:** ATF4, ER stress, lipid metabolism, prostate cancer, therapy resistance, Cancer, Metabolism

## Abstract

Despite the clinical benefit of androgen‐deprivation therapy (ADT), the majority of patients with advanced prostate cancer (PCa) ultimately develop lethal castration‐resistant prostate cancer (CRPC). In this study, we identified thioesterase superfamily member 6 (THEM6) as a marker of ADT resistance in PCa. THEM6 deletion reduces *in vivo* tumour growth and restores castration sensitivity in orthograft models of CRPC. Mechanistically, we show that the ER membrane‐associated protein THEM6 regulates intracellular levels of ether lipids and is essential to trigger the induction of the ER stress response (UPR). Consequently, THEM6 loss in CRPC cells significantly alters ER function, reducing *de novo* sterol biosynthesis and preventing lipid‐mediated activation of ATF4. Finally, we demonstrate that high THEM6 expression is associated with poor survival and correlates with high levels of UPR activation in PCa patients. Altogether, our results highlight THEM6 as a novel driver of therapy resistance in PCa as well as a promising target for the treatment of CRPC.

## Graphical abstract

Resistance to androgen‐deprivation therapy (ADT) leads to the development of lethal castration‐resistant prostate cancer. This study highlights THEM6 as a clinically relevant protein that supports ADT‐induced rewiring of lipid metabolism and sustains UPR activation, thus promoting ADT resistance.

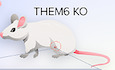

**The paper explained**

**Problem**

Androgen‐deprivation therapy (ADT) is the standard of care for patients with non‐resectable prostate cancer (PCa). Despite initial clinical benefit, patients ultimately relapse and develop lethal castration‐resistant prostate cancer (CRPC).

**Results**

We identified the uncharacterised protein THEM6 as a protein associated with ADT resistance in several *in vitro* and *in vivo* models of CRPC. Functionally, THEM6 loss impaired tumour growth and increased tumour response to ADT in PCa orthografts. Mechanistically, THEM6 was located at the membrane of the endoplasmic reticulum (ER) and primarily regulated ether lipid metabolism. Consequently, THEM6 expression was required to maintain sterol biosynthesis and to sustain activation of the ER stress response (UPR), two ER‐related processes that promote ADT resistance. Finally, we demonstrated that THEM6 levels were elevated in treatment‐resistant patient biopsies, associated with unfavourable patient outcome and correlated with sustained UPR activation in PCa patients.

**Impact**

The results highlight THEM6 as a clinically relevant marker of ADT resistance in prostate cancer. Because of its role in the regulation of lipid metabolism and ER biology, THEM6 represents a potential novel therapeutic target for the treatment of CRPC.

## Introduction

Androgen‐deprivation therapy (ADT) and androgen receptor (AR)‐targeted therapies have significantly improved outcomes for patients suffering from advanced prostate cancer (PCa). However, treatment resistance ultimately leads to the development of lethal castration‐resistant prostate cancer (CRPC), which remains a major therapeutic challenge.

Resistance to treatment is accompanied by a plethora of cellular and metabolic adaptations that allow cancer cells to cope with stress‐inducing factors (Marine *et al*, 2020). Together with mitochondria, the endoplasmic reticulum (ER) plays a central role in the regulation of stress‐signalling pathways. Indeed, the ER is critical for the establishment of a complex stress response, termed the unfolded protein response (UPR), that orchestrates the cellular adaptation to various perturbations such as impairment of protein or lipid homeostasis. Activation of the UPR relies on the coordinated action of three major branches, each of them characterised by the activity of a specific ER stress sensor: Inositol Requiring Enzyme 1 (IRE1α), PRKR‐like Endoplasmic Reticulum Kinase (PERK) and Activating Transcription Factor 6 (ATF6). In cancer, correct induction of the UPR is required to support oncogenic transformation (Hart *et al*, 2012). Persistent activation of ER stress responses can further promote tumour progression, metastasis dissemination and resistance to therapy (Chen & Cubillos‐Ruiz, 2021). Therefore, clinical targeting of the UPR, alone or in combination with other treatment modalities, is regarded as a promising strategy for the treatment of aggressive cancer (Logue *et al*, 2018; Xie *et al*, 2018; Zhao *et al*, 2018; Jin & Saatcioglu, 2020). This strategy is particularly effective in the context of PCa, where inhibition of the IRE1a branch of the UPR significantly impairs tumour growth and activation of c‐MYC signalling (Sheng *et al*, 2019), an important feature of ADT resistance (Bernard *et al*, 2003). Similarly, targeting of the PERK‐eIF2a‐ATF4 branch of the UPR effectively reduces tumour progression and metastasis dissemination in preclinical models of CRPC (Nguyen *et al*, 2018), while ATF4 signalling is essential for PCa growth and survival (Pallmann *et al*, 2019).

The ER is also the primary site of lipid and cholesterol biosynthesis. Changes in lipid homeostasis, such as impaired membrane lipid saturation (Pineau *et al*, 2009) or imbalance in the levels of phospho‐ and sphingolipid species (Han *et al*, 2010; Thibault *et al*, 2012), can also contribute to ER stress. Recently, we and others demonstrated the importance of lipid remodelling in tumour resistance to antiandrogen therapy (Blomme *et al*, 2020; Tousignant *et al*, 2020). Thus, targeting lipid‐mediated ER stress could be considered as a potential therapeutic option for the treatment of CRPC.

Acyl‐CoA thioesterases (ACOTs) are a class of enzymes that hydrolyse acyl‐CoA molecules. In contrast with type I enzymes, type II ACOTs are related by structure rather than sequence, as evidenced by their low levels of sequence similarity (Cohen, 2013). By contrast, type II ACOTs are characterised by the presence of an evolutionarily conserved domain, the “Hotdog” domain, which confers the thioesterase enzymatic activity (Pidugu *et al*, 2009). Type II ACOTs also include members of the Thioesterase Superfamily (THEM), which primarily function to deactivate fatty acyl‐CoA thioesters and generate free fatty acids (FA) and CoA. THEM proteins are involved in the regulation of intracellular FA trafficking and have been shown to influence both lipogenesis and beta‐oxidation depending on the physiological context (Tillander *et al*, 2017). Interestingly, overexpression of thioesterase superfamily member 6 (THEM6/c8orf55) has been reported in several cancer types in a proteomic study focusing on the identification of colon cancer biomarkers (Kume *et al*, 2014). However, the biological role of THEM6 in normal and pathological physiology remains unexplored.

In this study, we identified THEM6 as a clinically relevant protein associated with ADT resistance in PCa. Mechanistically, we show that THEM6 maintains lipid homeostasis by controlling intracellular levels of ether lipids. Consequently, THEM6 expression is critical for ER membrane integrity, sterol biosynthesis and ATF4 activation under ADT conditions. Importantly, high THEM6 expression is frequently observed in CRPC patient biopsies, correlates with high levels of UPR activation and is associated with shortened patient survival. Taken together, our results highlight the potential of THEM6 as a future therapeutic option for the treatment of CRPC.

## Results

### THEM6 expression is increased upon ADT resistance

**Figure 1:**
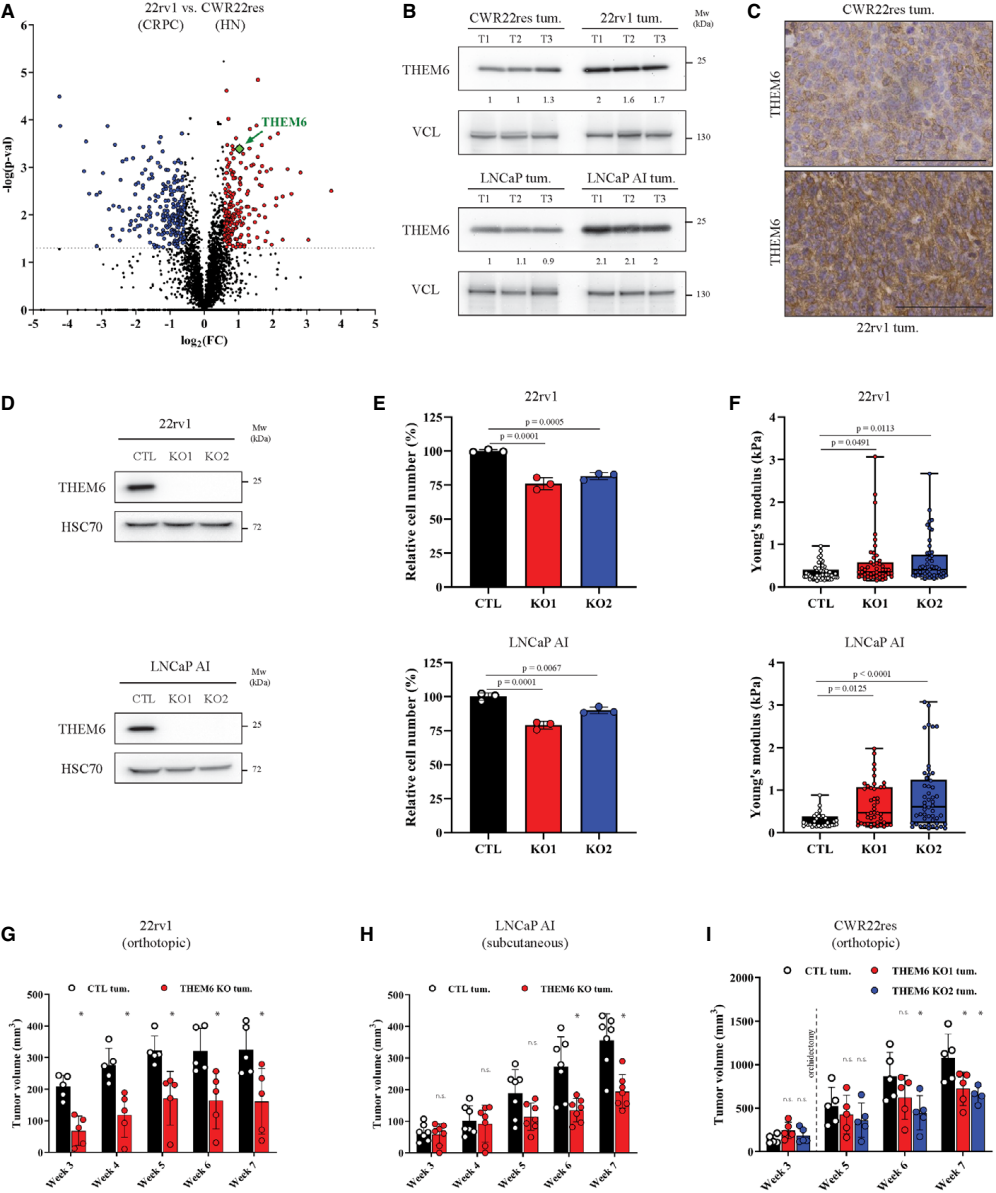
Loss of THEM6 impairs PCa tumour growth following ADT Volcano plot of the differentially modulated proteins in 22rv1 (CRPC) versus CWR22res (HN) tumours. Red and blue dots represent the proteins that are significantly up‐ and down‐regulated, respectively (*P*‐value < 0.05, FC = 1.5).Western blot analysis of THEM6 expression in CRPC (22rv1 and LNCaP AI) and HN (CWR22res and LNCaP) prostate orthografts. VCL was used as a sample loading control.IHC staining of THEM6 expression in CWR22res and 22rv1 orthografts. Scale bar represents 100 µm.Western blot analysis of THEM6 expression in CTL and THEM6 KO CRPC cells. HSC70 was used as a sample loading control.Proliferation of CTL and THEM6 KO CRPC cells after 72 h. Data are expressed as a percentage of CTL cells.Cell stiffness (Young's modulus) of CTL and THEM6 KO CRPC cells measured by atomic force microscopy.Tumour volume (measured by ultrasound) of CTL and THEM6 KO 22rv1‐derived orthografts developed in surgically castrated mice.Tumour volume of CTL and THEM6 KO LNCaP AI‐derived xenografts developed in surgically castrated mice.Tumour volume (measured by ultrasound) of CTL and THEM6 KO CWR22res‐derived orthografts. Orchidectomy was performed 3 weeks after cell implantation. Data information: Panels (E, G, H, I) Data are presented as mean values ± SD. Panel (F) Centre line corresponds to median of data, top and bottom lines correspond to maximal and minimal values. Statistical analysis: (E, F) One‐way ANOVA with a Dunnett's multiple comparisons test. (G, H) two‐tailed Mann–Whitney *U*‐test. (I) Kruskal–Wallis test. Data reproducibility: Panel (A) *n* = 3 tumours per group. Panel (B) *n* = 1 gel loaded with three prostate orthografts per condition. Panels (C, D) representative image from 3 independent biological experiments. Panel (E) *n* = 3 independent biological experiments. Panel (F) (up): *n* = 46 (CTL); 47 (KO1); 49 (KO2) cells measured. Panel (F) (down): *n* = 31 (CTL); 43 (KO1); 50 (KO2) cells measured. Panels (G, I) *n* = 5 mice per group. Panel (H) *n* = 7 mice per group. Source data are available online for this figure.

**Figure EV1:**
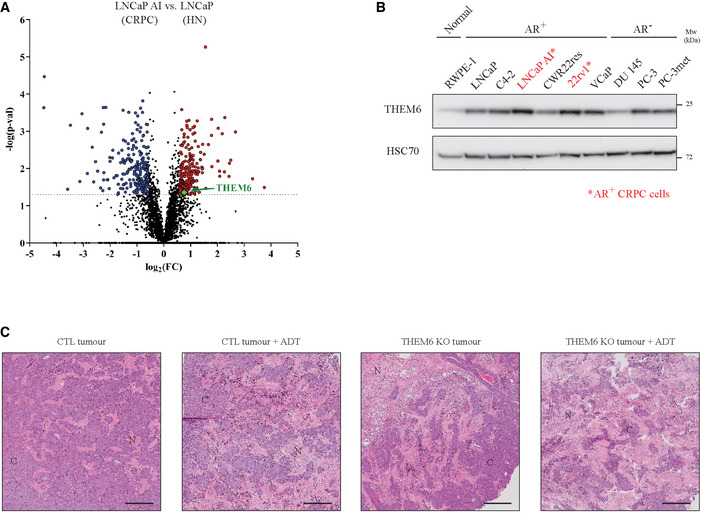
THEM6 is overexpressed following ADT resistance Volcano plot of the differentially modulated proteins in LNCaP AI (CRPC) versus LNCaP (HN) tumours. Red and blue dots represent the proteins that are significantly up‐ and down‐regulated, respectively (*P*‐value ≤ 0.05, FC = 1.5).Western blot analysis of THEM6 expression in PCa cells. HSC70 was used as a sample loading control. Within a panel of PCa cell lines, androgen receptor (AR)‐positive CRPC (namely 22rv1 and LNCaP AI) cells express THEM6 at higher levels than their respective isogenic HN (LNCaP and CWR22res) counterparts.Representative pictures of haematoxylin/eosin staining on orthografts from CWRres CTL and THEM6 KO tumours. C = cancer cells; N = necrotic area. Scale bar represents 1,000 µm. Data information: Data reproducibility: (A) *n* = 3 tumours per group. (B) representative image from three independent biological experiments. (C) representative image from three tumours per group. Source data are available online for this figure.

We previously performed an in‐depth proteomic analysis comparing *in vivo* hormone‐naïve (HN) and castration‐resistant (CRPC) orthograft models of PCa (Martinez *et al*, 2021). For this purpose, we injected matched pairs of isogenic human PCa cell lines (namely 22rv1/CWR22res and LNCaP AI/LNCaP) into the prostate of immuno‐deficient mice to generate tumour orthografts. Importantly, CRPC cells (22rv1 and LNCaP AI) were routinely cultured in absence of androgens and subsequently injected into castrated mice, while the matched HN cells (CWR22res and LNCaP) were cultured in androgen‐containing medium and further injected into intact mice. We then sought to identify the proteins that were commonly regulated following ADT in the different models. From this comparison, we identified THEM6/c8orf55 as a protein significantly upregulated in CRPC tumours (22rv1 and LNCaP AI) when compared to HN counterparts (CWR22res and LNCaP, respectively; Figs 1A and EV1A). Increased THEM6 levels in CRPC conditions were validated *in vivo* (Fig 1B and C) and *in vitro* (Fig EV1B). Of note, THEM6 expression was the lowest in normal prostate epithelial cells (RWPE‐1) when compared with multiple PCa cell lines (Fig EV1B). Immunohistochemistry (IHC) staining of prostate orthografts confirmed THEM6 over‐expression in CRPC tumours and highlighted strong cytoplasmic and perinuclear staining in tumour epithelial cells (Fig 1C).

### Loss of THEM6 affects PCa tumour growth and increases tumour response to ADT

To investigate the role of THEM6 in CRPC, we generated stable CRISPR‐based THEM6 knockout cell lines (hereafter referred to as THEM6 KO; Fig 1D). On average, THEM6 KO resulted in a mild (~ 20–25%) decrease in CRPC cell proliferation (Fig 1E). Interestingly, THEM6‐depleted cells also displayed increased cell stiffness (Fig 1F), a phenotype which is indicative of membrane and cytoskeleton reorganisation and associated with impaired migratory abilities (Rudzka *et al*, 2019). *In vivo*, loss of THEM6 significantly reduced tumour volume in a CRPC model of 22rv1‐derived orthografts (assessed by ultrasonography, Fig 1G). Similarly, THEM6 KO significantly impaired the growth of LNCaP AI tumours developed in castrated mice (Fig 1H). In both models, orchidectomy was performed at the time of cell transplantation to mimic the *in vivo* environment of ADT. To complement the use of the CRPC models, we tested whether THEM6 loss would also sensitise PCa tumours to acute ADT treatment. For this purpose, we applied the CWR22res orthograft model which allows us to study the effects of ADT on pre‐established tumours (orchidectomy 3 weeks post‐injection), thus better resembling treatment in clinical patients (Patel *et al*, 2018). Similar to 22rv1 and LNCaP AI, THEM6 KO strongly impaired tumour growth following ADT in the CWR22res orthograft model (Fig 1I). In addition to decreased tumour size, THEM6‐deficient orthografts exhibited large necrotic areas and decreased cellularity, especially under ADT conditions (Fig EV1C). Taken together, these data support a pro‐tumorigenic role for THEM6 in PCa.

### THEM6 regulates cellular lipid metabolism

**Figure 2:**
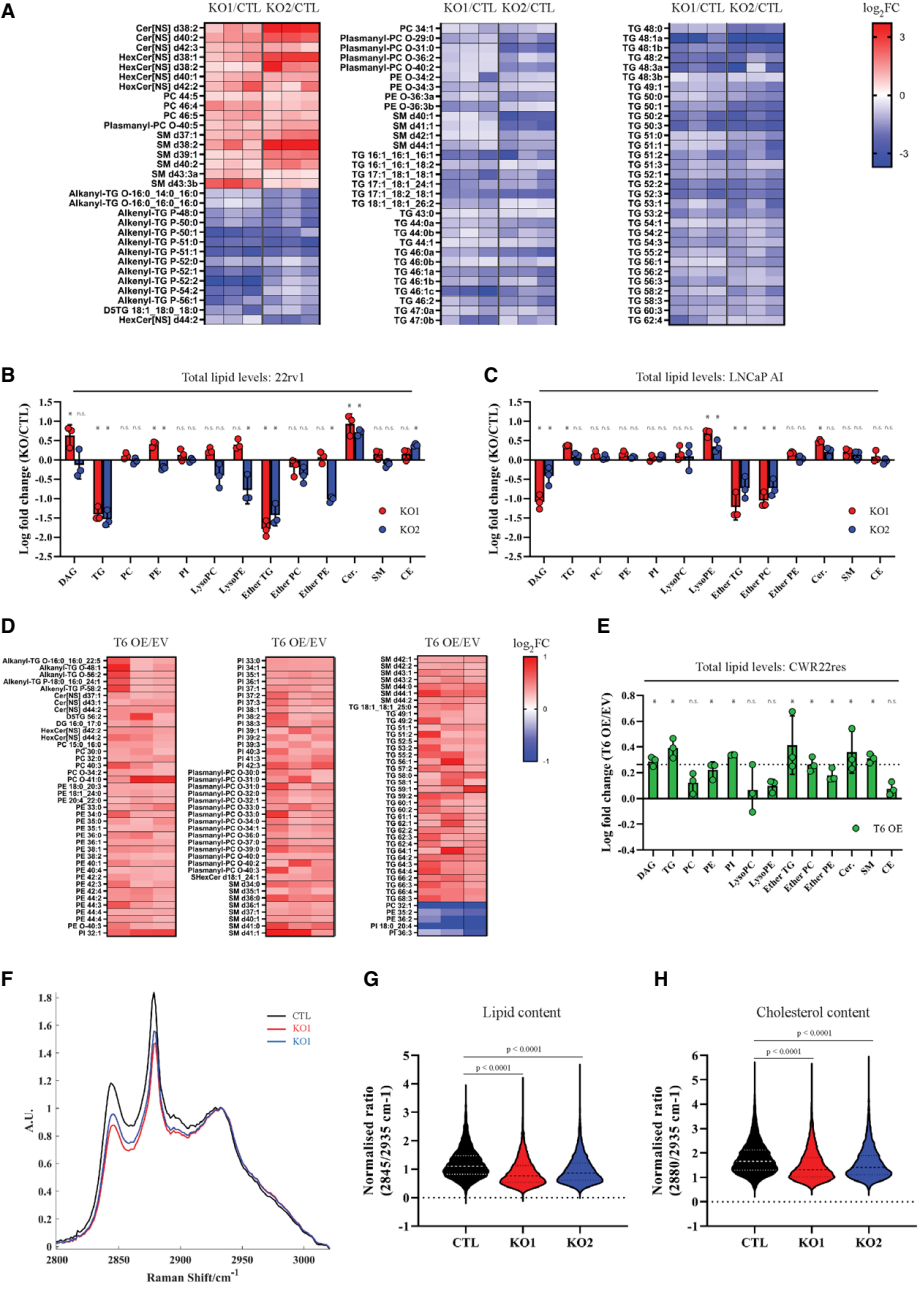
Loss of THEM6 alters lipid homeostasis AHeatmap illustrating the steady‐state levels of significantly regulated lipids in THEM6 KO 22rv1 cells when compared to CTL (*P* ≤ 0.05, FC = 1.3). Values are expressed as log_2_(FC).B, CChanges in lipid content (total amount) observed in THEM6 KO CRPC cells when compared to CTL.DHeatmap illustrating the steady‐state levels of significantly regulated lipids in THEM6 OE CWR22res cells when compared to EV (*P* ≤ 0.05, FC = 1.2). Values are expressed as log_2_(FC).EChanges in lipid content (total amount) observed in THEM6 OE CWR22res cells when compared to EV.FAverage Raman spectra of CWR22res CTL and THEM6 KO orthografts.G, HQuantification of tumour lipid (2,845 cm^−1^‐peak) and cholesterol (2,880 cm^−1^‐peak) content obtained from (F). Data information: AU, arbitrary unit. Panels (B, C, E) Data are presented as mean values ± SD. Panels (G, H) Centre line corresponds to median of data, top and bottom lines correspond to upper and lower quartiles. Statistical analysis: (B, C) *P‐value < 0.05 using a one‐way ANOVA with a Dunnett's multiple comparisons test. (E) *P‐value < 0.05 using a two‐tailed Student's *t*‐test. (G, H) Kruskal–Wallis test. Data reproducibility: Panels (A, B, C, D, E) *n* = 3 independent biological experiments. Panel (F) *n* = 4 mice per group. Panel (G, H) *n* = 6,581 (CTL); 12,047 (KO1); 6,493 (KO2) peak intensities that were extracted from four mice per group. CE, Cholesteryl ester; Cer, Ceramide; DAG, diacylglycerol; EV, Empty Vector; LysoPC, lysophosphatidylcholine; LysoPE, lysophosphatidylethanolamine; PC, phosphatidylcholine; PE, phosphatidylethanolamine; PI, phosphatidylinositol; SM, sphingomyelin; TG, triglyceride. Source data are available online for this figure.

**Figure EV2:**
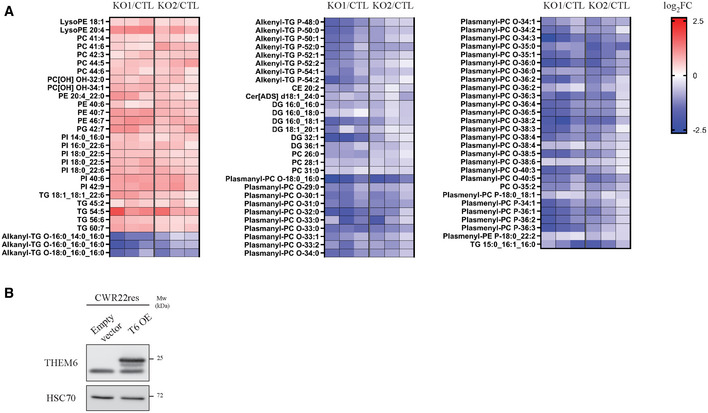
Loss of THEM6 alters lipid homeostasis Heatmap illustrating the steady‐state levels of significantly regulated lipids in THEM6 KO LNCaP AI cells when compared to CTL (*P* ≤ 0.05, FC = 1.3). Values are expressed as log_2_(FC).Western blot analysis of THEM6 expression in CWR22res cells overexpressing THEM6 (T6 OE). HSC70 was used as a sample loading control. Data information: Data reproducibility: (A) *n* = 3 independent biological experiments. (B) representative image from two independent biological experiments. Source data are available online for this figure.

As a member of the THEM superfamily, THEM6 exhibits an evolutionarily conserved “Hotdog” domain predicted to confer thioesterase activity (Tillander *et al*, 2017). Rewiring of lipid metabolism is a common feature of ADT resistance (Blomme *et al*, 2020). Therefore, we hypothesised that THEM6 could participate in the lipid rearrangement required for CRPC progression. To evaluate the impact of THEM6 loss on the lipid composition of CRPC cells, we compared the lipid profiles of control (CTL) and THEM6 KO cells using LC‐MS lipidomics. Strikingly, loss of THEM6 in 22rv1 cells resulted in a profound remodelling of the cellular lipidome. THEM6 depletion was associated with a strong reduction in the intracellular levels of multiple triglyceride (TG) and ether lipid species (ether triglycerides [ether TG] and ether phospholipids [ether phosphatidylcholines (ether PC) or ether phosphatidylethanolamines (ether PE)]). In contrast, THEM6 KO cells displayed increased amounts of ceramides (Fig 2A). In addition to specific lipid changes, THEM6 KO also significantly affected the total amount of TGs, ether TGs and ceramides in 22rv1 cells (Fig 2B). Interestingly, the intracellular levels of several ether lipid species (but not TG) were also strongly reduced in LNCaP AI THEM6 KO cells when compared to their respective CTL (Figs 2C and EV2A). In line with these data, transient overexpression of THEM6 in CWR22res cells (Fig EV2B) resulted in increased levels of specific lipid molecules, including multiple species of TGs, ether‐TGs and ether‐PCs (Fig 2D). Total amounts of TGs and ether‐TGs were also significantly increased following THEM6 overexpression (Fig 2E). Taken together, these results suggest that THEM6 loss might primarily affect ether lipid homeostasis. We further assessed the effect of THEM6 KO on lipid content *in vivo* by performing Raman spectroscopy on ADT‐treated CWR22res orthografts. Raman spectroscopy allows assessment and quantitation of lipids (band at 2,845 cm^−1^) and cholesterol (band at 2,880 cm^−1^) content on paraffin‐embedded tumour slides in a non‐destructive manner (Fig 2F). Results from the analysis provided evidence that THEM6‐deficient tumours (Fig 1I) display significantly less lipids (Fig 2G) and cholesterol (Fig 2H) than CTL tumours, thus confirming a role for THEM6 in the maintenance of the tumour lipidome.

### THEM6 is an ER membrane protein that is essential to maintain ER integrity

**Figure 3:**
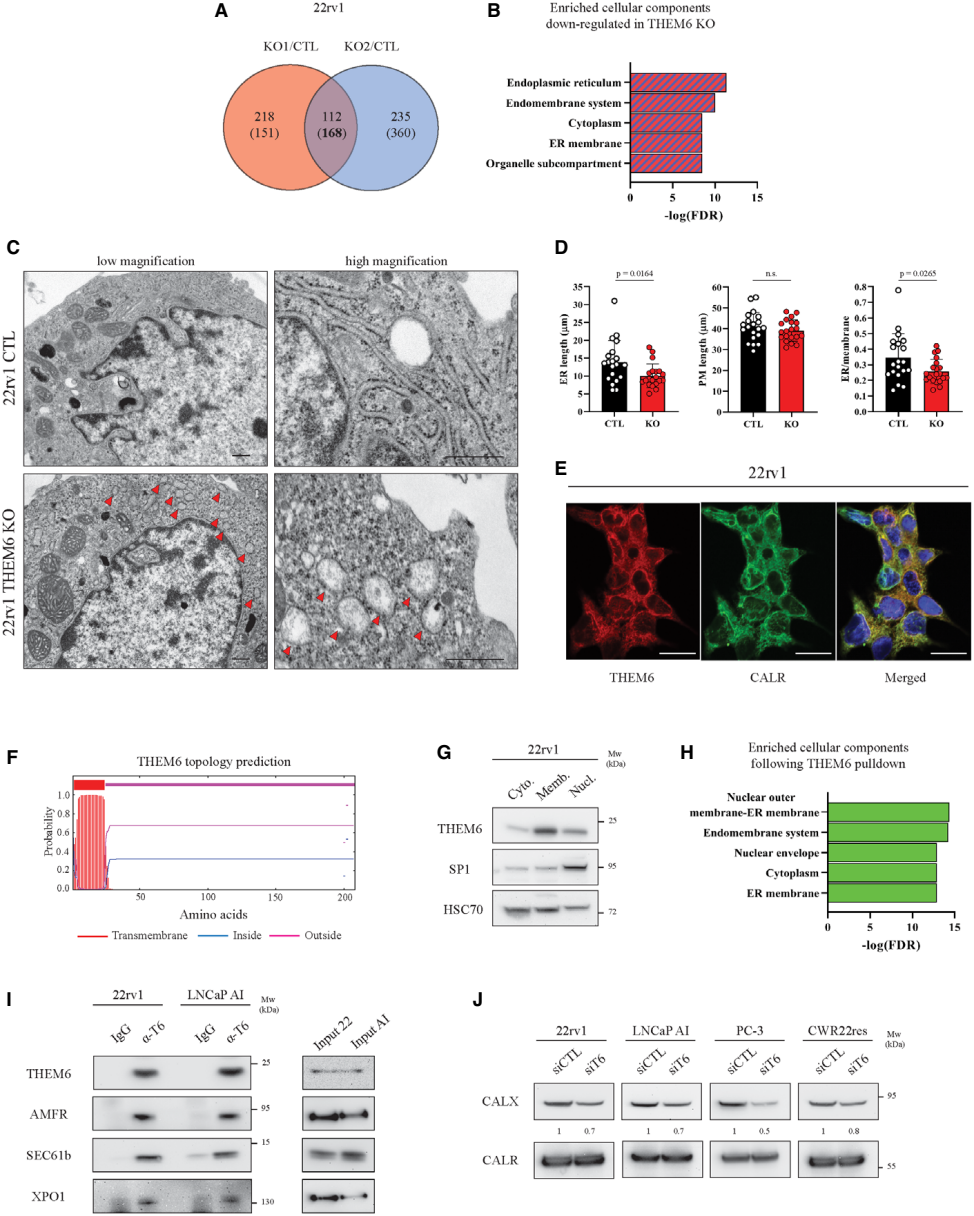
THEM6 interacts with multiple ER membrane components involved in protein transport Venn diagram highlighting commonly modulated proteins (*P*‐value ≤ 0.05, FC = 1.3) in THEM6 KO 22rv1 cells (two clones) when compared to CTL. Up‐regulated proteins are on top; Down‐regulated proteins are into brackets.Enriched cellular components commonly down‐regulated in THEM6 KO 22rv1 cells (two clones) when compared to CTL.Representative electron microscopy (EM) pictures of CTL and THEM6 KO 22rv1 cells taken at low (left) and high (right) magnification. Red arrows point towards abnormal ER structure. Scale bar represents 500 nm.Quantification of ER membrane length, plasma membrane length and ER/plasma membrane ratio using the EM pictures from (C).Immunofluorescence showing co‐localisation of THEM6 and the ER marker calreticulin in 22rv1 cells. Scale bar represents 20 µm.Prediction of transmembrane domain in the sequence of the THEM6 protein. Topology prediction was performed using the TMHMM server (http://www.cbs.dtu.dk/services/TMHMM).Western blot analysis of THEM6 expression in cytoplasmic (cyto.), membrane/organelle (memb.) and nuclear fractions (nucl.) of 22rv1 cells. HSC70 and SP1 were used as cytoplasmic and nuclear‐enriched markers, respectively.Enriched cellular components in THEM6‐interacting proteins following THEM6 pulldown in T6 OE HEK293 cells.Western blot analysis of THEM6, AMFR, SEC61b and XPO1 expression in CRPC cells following THEM6 immunoprecipitation in CRPC cells.Western blot analysis of CALX and CALR expression in PCa cells following THEM6 silencing. Data information: Panels (B, H) Enrichment analysis was performed using the STRING database (http://string‐db.org). Panel (D) Data are presented as mean values ± SD. Statistical analysis: (D) Unpaired *t*‐test. Data reproducibility: Panel (D) *n* = 20 cells/condition. Panels (E, G, J) representative image from three independent biological experiments. Panel (I) representative image from two independent biological experiments. Source data are available online for this figure.

**Figure EV3:**
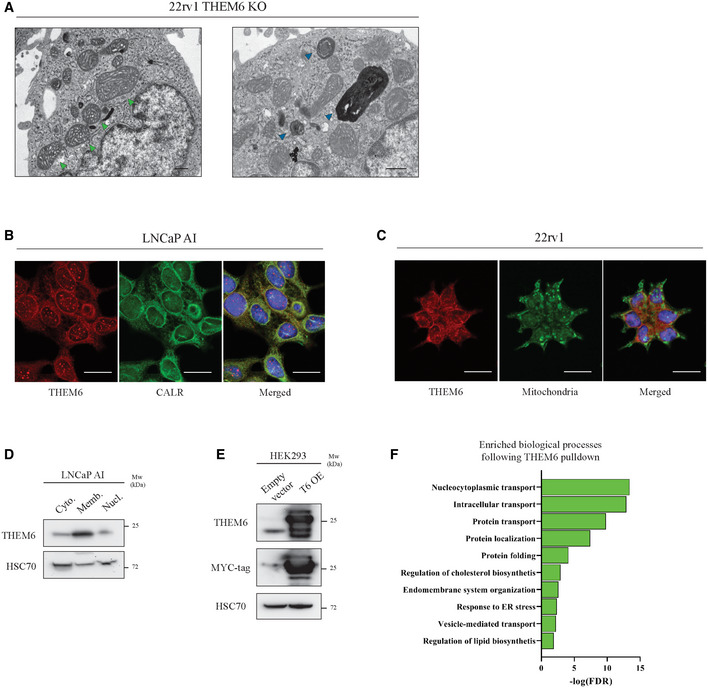
THEM6 is an ER membrane protein Representative electron microscopy pictures of THEM6 KO 22rv1 cells. Green and blue arrows point towards enlarged mitochondria and abnormal lysosomal structure, respectively. Scale bar represents 500 nm.Immunofluorescence showing co‐localisation of THEM6 and the ER marker calreticulin in LNCaP AI cells. Scale bar represents 20 µm.Immunofluorescence showing distinct localisations for THEM6 and mitochondria in 22rv1 cells. Scale bar represents 20 µm.Western blot analysis of THEM6 expression in cytoplasmic (cyto.), membrane/organelle (memb.) and nuclear fractions (nucl.) of LNCaP AI cells. HSC70 was used as cytoplasmic‐enriched marker.Western blot analysis of THEM6 and MYC‐tag expression in HEK293 cells overexpressing a MYC‐tagged version of THEM6 (T6 OE). HSC70 was used as a sample loading control.Enriched biological processes in THEM6‐interacting proteins following THEM6 pulldown in T6 OE HEK293 cells. Data information: Panel (F) Enrichment analysis was performed using the STRING database (http://string‐db.org). Data reproducibility: (B, C, D) representative image from 3 independent biological experiments. (E) representative image from two independent biological experiments. Source data are available online for this figure.

To gain further insights into the role of THEM6 in cancer cells, we performed a proteomic comparison of 22rv1 cells proficient or depleted for THEM6 (Fig 3A). Proteomic analysis highlighted a large cluster of ER‐related proteins that were significantly down‐regulated in the absence of THEM6 (FC = 1.3, *P* < 0.05, Fig 3B and Table EV1). In addition, the majority of these proteins were described as membrane proteins (Fig 3B). Lipids are essential components of biological membranes, and the ER is particularly sensitive to lipid perturbation (Volmer & Ron, 2015). Electron microscopy confirmed the negative impact of THEM6 depletion on ER morphology. Indeed, THEM6 KO cells presented with abnormal ER, exhibiting rounded structures with highly dilated lumens (Fig 3C). To assess the impact of THEM6 loss on the ER membrane, we further quantified the length of the ER, the length of the plasma membrane (PM) as well as the ratio ER/plasma membrane. While THEM6 loss did not affect the PM length, the size of the ER membrane and therefore the ER/plasma membrane ratio were reduced in THEM6 KO cells in comparison to CTL (Fig 3D). The presence of abnormally enlarged mitochondria and large multilamellar lysosomes was also frequently observed in THEM6 KO cells, potentially reflecting general membrane perturbations following THEM6 loss (Fig EV3A). Interestingly, THEM6 strongly co‐localised with the ER marker calreticulin (Figs 3E and EV3B), but not with mitochondria (Fig EV3C). This result suggests that THEM6 is predominantly associated with the ER, contrasting with the mitochondrial localisation reported for other THEM superfamily members (THEM2/4/5) (Cohen, 2013). Furthermore, topological analysis of the THEM6 protein sequence highlighted a 17‐amino acid signal peptide that corresponds to a well‐defined N‐terminal transmembrane domain (Fig 3F). Finally, Western blot (WB) analysis after subcellular fractionation confirmed the presence of THEM6 in the insoluble organellular/membrane fraction of CRPC cells (Figs 3G and EV3D).

### THEM6 interacts with multiple ER membrane components involved in protein transport

Lipid metabolism and protein homeostasis are highly interconnected processes (Surma *et al*, 2013). In particular, ether lipids and plasmalogen derivatives play a key role in regulating membrane protein trafficking and homeostasis (Jiménez‐Rojo & Riezman, 2019). Due to its localisation at the ER membrane and its role in regulating lipid balance, THEM6 might therefore be important for the maintenance of protein homeostasis in the ER. In line with this idea, pull‐down experiments followed by MS analysis identified 152 proteins that significantly interacted with THEM6 in THEM6‐overexpressing HEK‐293 cells (FC = 10, *P* < 0.05, Fig EV3E and Table EV2). Most of the THEM6 interactors were located at the ER membrane, or at the interface between the ER and the nucleus (Fig 3H) and were mainly involved in protein transport (Fig EV3F). Among others, several exportins, importins, transportins and components of the oligosaccharyltransferase (OST) complex were identified as strong THEM6‐interacting partners (Table EV2). We further validated the results of our interactomics by performing immunoprecipitation experiments in 22rv1 and LNCaP AI cells. We confirmed the direct interactions between endogenous THEM6 and several membrane proteins from different subcellular compartments (XPO1 at the outer nuclear membrane, AMFR and SEC61b in the ER) in both CRPC cell lines, thus indicating that the function of THEM6 in the ER might be conserved across different cell types (Fig 3I). Finally, we observed that acute THEM6 silencing in PCa cell lines led to a consistent decrease in the expression of the ER membrane‐associated lectin calnexin (CALX) without affecting the levels of the soluble homolog calreticulin (CALR; Fig 3J). Taken together, these results suggest that THEM6 is important to maintain the integrity of the ER membrane and that THEM6 loss might preferentially affect the expression of membrane proteins.

### THEM6 loss affects *de novo* sterol and FA synthesis in cancer cells

**Figure 4:**
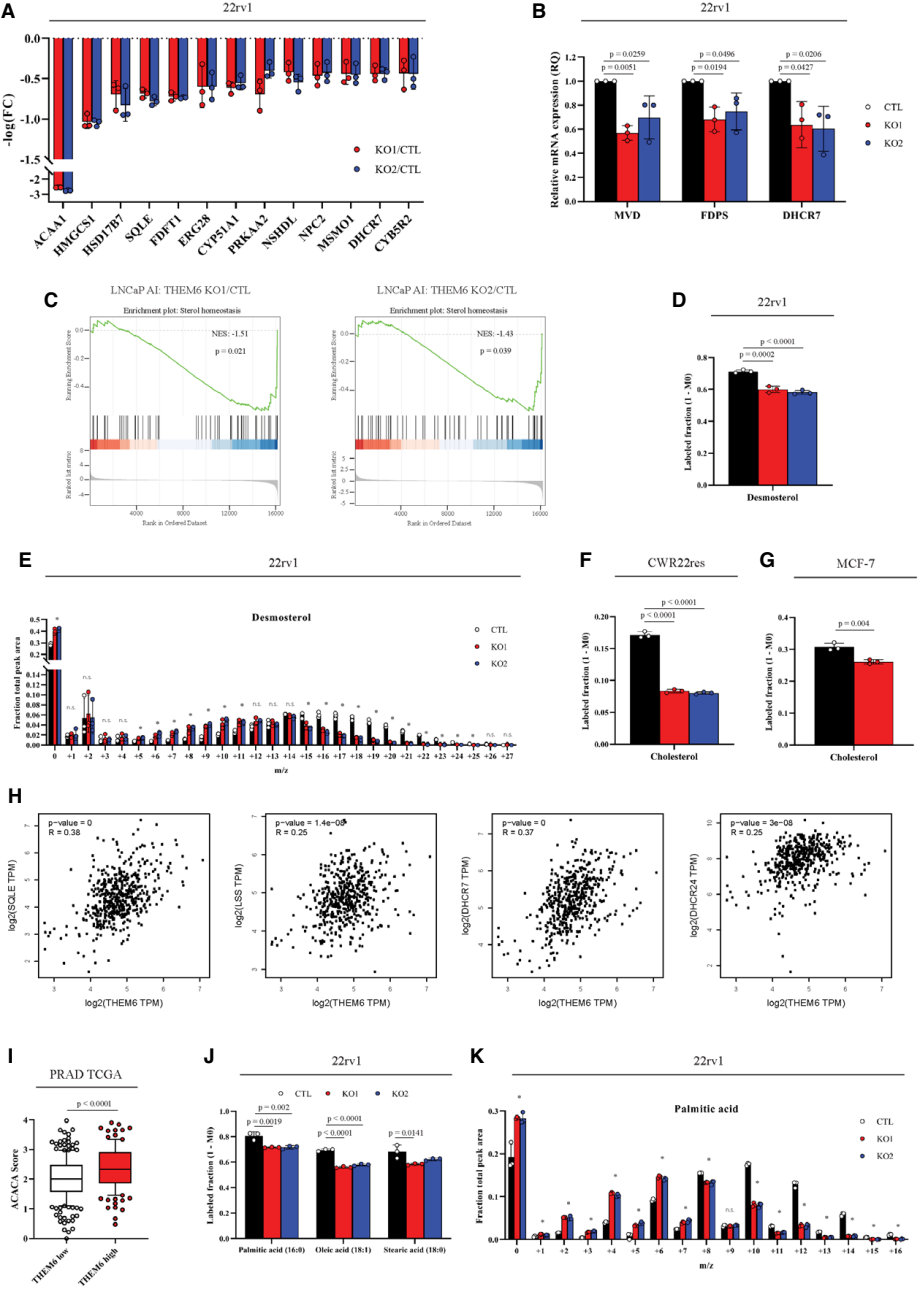
Loss of THEM6 affects *de novo* sterol and fatty acid synthesis Proteomic analysis highlighting proteins associated with sterol biosynthetic pathway and significantly down‐regulated in THEM6 KO 22rv1 cells when compared to CTL (*P*‐value ≤ 0.05, FC = 1.3).RT–qPCR analysis of *MVD, FDPS* and *DHCR7* expression in CTL and THEM6 KO 22rv1 cells. *CASC3* was used as a normalising control.Gene set enrichment plots analysed from THEM6 KO LNCaP AI cells using the GOBP “Sterol homeostasis” gene set.Labelled desmosterol fraction derived from ^13^C‐glucose and ^13^C‐glutamine in CTL and THEM6 KO 22rv1 cells after 72 h of incubation.Relative isotopologue distribution of desmosterol in CTL and THEM6 KO 22rv1 cells after 72 h of incubation.Labelled cholesterol fraction derived from ^13^C‐glucose and ^13^C‐glutamine in CTL and THEM6 KO CWR22res cells after 72 h of incubation.Labelled cholesterol fraction derived from ^13^C‐glucose and ^13^C‐glutamine in CTL and THEM6 KO MCF‐7 cells after 72 h of incubation.Pearson's correlation analysis of *SQLE*, *LSS, DHCR7* and *DHCR24* with *THEM6* using the PRAD TCGA dataset. Results were obtained using the GEPIA website http://gepia.cancer‐pku.cn/.Differential expression of ACACA in high and low THEM6 tumours according to the PRAD TCGA dataset.Labelled palmitic, oleic and stearic acid fractions derived from ^13^C‐glucose and ^13^C‐glutamine in CTL and THEM6 KO 22rv1 cells after 72 h of incubation.Relative isotopologue distribution of palmitic acid in CTL and THEM6 KO 22rv1 cells after 72 h of incubation. Data information: Panels (A, B, D, E, F, G, J, K) Data are presented as mean values ± SD. Panel (I) Centre line corresponds to median of data, top and bottom of box correspond to 90^th^ and 10^th^ percentile, respectively. Whiskers extend to adjacent values (minimum and maximum data points not considered outliers). Statistical analysis: (B, D, E, F, J, K) *P‐value < 0.05 using one‐way ANOVA with a Dunnett's multiple comparisons test. (G) *P‐value < 0.05 using a two‐tailed Student *t*‐test. (I) two‐tailed Mann–Whitney *U*‐test. Data reproducibility: Panels (A, B) *n* = 3 independent biological experiments. Panels (D, E, F, G, J, K) *n* = 3 independent wells from the same cell culture. Panel (I) *n* = 225 tumours for THEM6 low and *n* = 121 tumours for THEM6 high. Source data are available online for this figure.

**Figure EV4:**
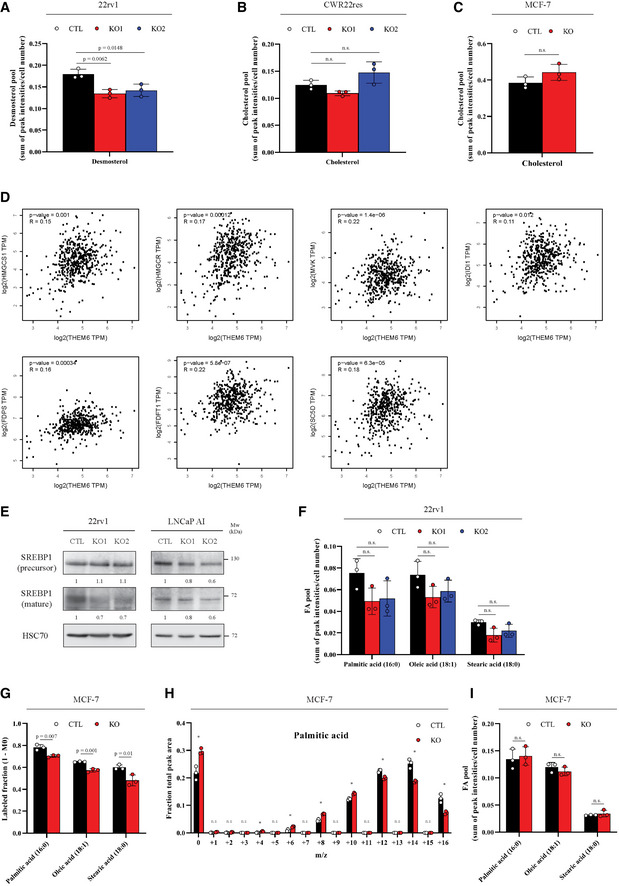
Loss of THEM6 affects *de novo* sterol and FA synthesis Total pool of desmosterol in CTL and THEM6 KO 22rv1 cells. Data extracted from Fig 4D and E.Total pool of cholesterol in CTL and THEM6 KO CWR22res cells. Data extracted from Fig 4F.Total pool of cholesterol in CTL and THEM6 KO MCF‐7 cells. Data extracted from Fig 4G.Pearson's correlation analysis of *HMGCS1, HMGCR, MVK, IDI1, FDPS, FDFT1, SC5D* with *THEM6* using the PRAD TCGA dataset. Results were obtained using the GEPIA website http://gepia.cancer‐pku.cn/.Western blot analysis of SREBP1 (precursor and mature forms) expression in CTL and THEM6 KO CRPC cells. HSC70 was used as a sample loading control.Total pool of palmitic, oleic and stearic acid in CTL and THEM6 KO 22rv1 cells. Data extracted from Fig 4J and K.Labelled palmitic, oleic and stearic acid fractions derived from ^13^C‐glucose and ^13^C‐glutamine in CTL and THEM6 KO MCF‐7 cells after 72 h of incubation.Relative isotopologue distribution of palmitic acid in CTL and THEM6 KO MCF‐7 cells after 72 h of incubation.Total pool of palmitic, oleic and stearic acid in CTL and THEM6 KO 22rv1 cells. Data extracted from (G, H). Data information: Panels (A, B, C, F, G, H, I) Data are presented as mean values ± SD. Statistical analysis: (A, B, F) One‐way ANOVA with a Dunnett's multiple comparisons test. (C, G, H, I) two‐tailed Student *t*‐test. Data reproducibility: (A, B, C, F, G, H, I) *n* = 3 independent wells from the same cell culture. (E) representative image from three independent biological experiments. Source data are available online for this figure.

In addition to protein trafficking, the ER is also the primary site for lipid and cholesterol synthesis. Therefore, we postulated that THEM6 depletion would impact these metabolic processes. Supporting this idea, our proteomic analysis of the 22rv1 THEM6‐deficient cells highlighted a down‐regulation of multiple proteins involved in sterol biosynthesis (Fig 4A). Sterol biosynthesis is of particular interest in the context of CRPC, as cholesterol serves as a precursor for *de novo* androgen synthesis and sustains ADT resistance (Chang *et al*, 2013; Patel *et al*, 2018). We first validated the decreased expression of several enzymes involved in sterol biosynthesis in 22rv1 THEM6 KO cells (Fig 4B). Similarly, RNAseq analysis of LNCaP AI cells highlighted a strong negative enrichment of a sterol homeostasis gene signature in the THEM6 KO cells (Fig 4C). Next, we functionally tested the effect of THEM6 loss on *de novo* sterol biosynthesis by incubating cancer cells with [U13C]‐glucose and [U13C]‐glutamine and following ^13^C incorporation into sterols using GC‐MS. Surprisingly, we were not able to detect a significant proportion of labelled cholesterol in 22rv1 cells. Instead, these cells accumulated large amounts of *de novo* synthesised desmosterol, an immediate precursor of cholesterol (Figs 4D and E and EV4A). In agreement with the proteomic data, 22rv1 THEM6 KO cells accumulated significantly less ^13^C‐enriched desmosterol than CTL cells (Fig 4D and E). In contrast to 22rv1 cells, the isogenic CWR22res cell line displayed a significant proportion of labelled cholesterol after incubation with [U13C]‐glucose and [U13C]‐glutamine. In this cell line, ^13^C‐enrichment of cholesterol was also significantly reduced following THEM6 depletion (Figs 4F and EV4B). To validate these findings, we also assessed the metabolic impact of THEM6 loss in the highly lipogenic and steroidogenic breast cancer MCF‐7 cell line (Fig 4G). In line with the results obtained with the PCa cell lines, we observed that THEM6 depletion in MCF‐7 cells resulted in decreased levels of *de novo* synthesised cholesterol (Figs 4G and EV4C). Furthermore, using the Prostate Adenocarcinoma (PRAD) TCGA dataset, we found that THEM6 expression strongly correlated with the expression of several enzymes involved in the late steps (SQLE, LSS, DHCR7 and DHCR24, Fig 4H), but not in the early steps (mevalonate pathway, Fig EV4D), of sterol biosynthesis, suggesting that THEM6 also contributes to this pathway in PCa patients. Similarly, unbiased analysis of the PRAD TCGA dataset revealed that Acetyl‐CoA Carboxylase (ACACA) and Fatty Acid Synthase (FASN), two regulatory enzymes of the fatty acid (FA) synthesis, were among the top up‐regulated proteins in THEM6‐enriched patient tumours (Fig 4I and Table EV3). Moreover, THEM6‐deficient cells displayed decreased expression (LNCaP AI, Fig EV4E) or activation (22rv1, Fig EV4E) of the lipogenic transcription factor SREBP‐1, pointing towards an altered lipid synthesis in these cells (Fig EV4E). Therefore, we assessed the contribution of THEM6 to FA synthesis by determining the relative proportions of ^13^C‐labelled palmitic, oleic and stearic acids in the presence and absence of THEM6 (Figs 4J and K, and EV4F). Similar to sterols, THEM6 KO cells accumulated significantly less labelled FA when compared to CTL, indicating reduced FA synthesis in the absence of THEM6. Importantly, the negative impact of THEM6 KO on *de novo* FA synthesis was also confirmed using the MCF‐7 cell line (Fig EV4G–I). Altogether, these results suggest that THEM6 expression is critical for the regulation of *de novo* lipid synthesis.

### THEM6 is required to trigger ATF4 induction in CRPC cells

**Figure 5:**
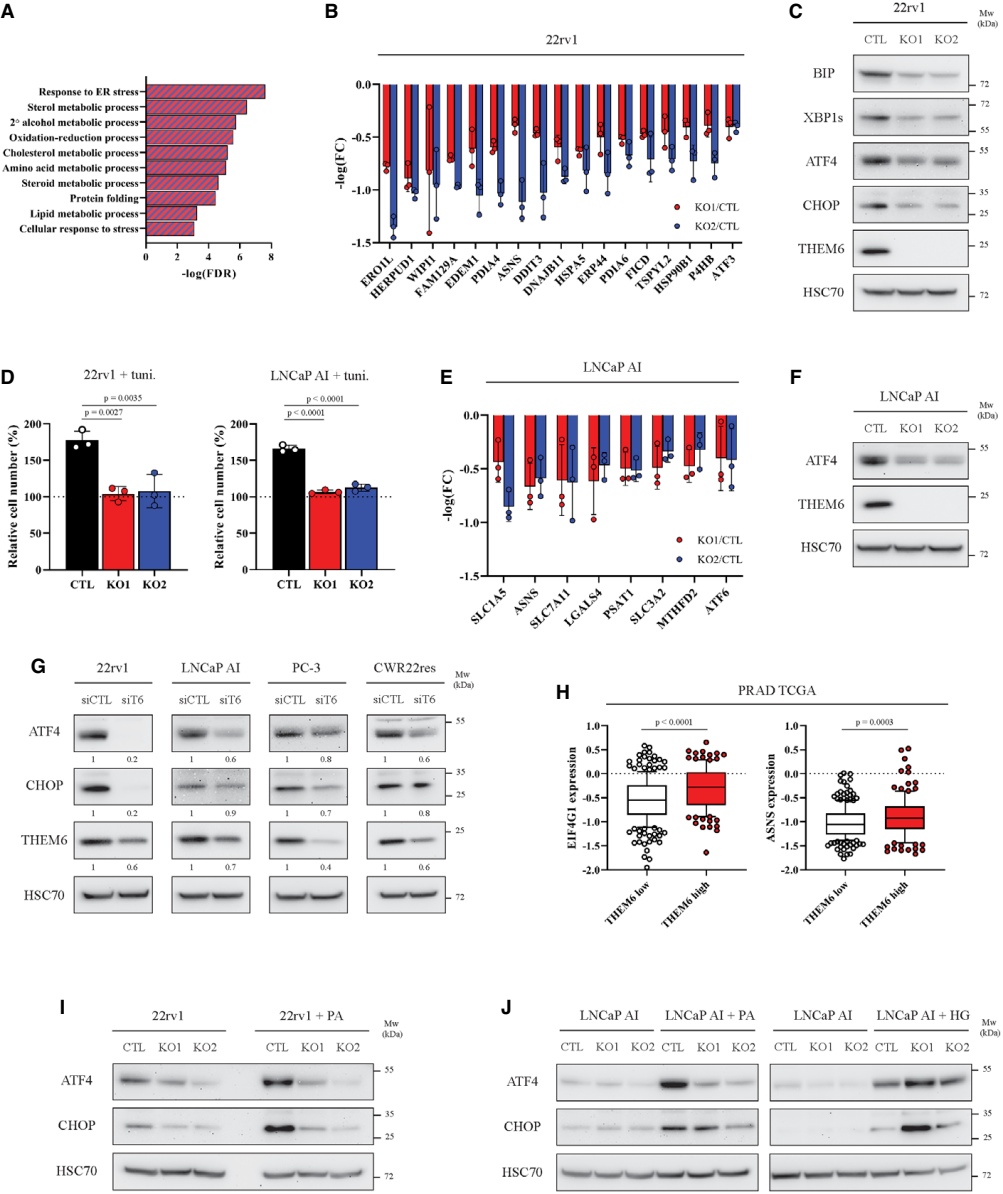
THEM6‐mediated lipid remodelling is required for UPR activation Enriched biological processes commonly down‐regulated in THEM6 KO 22rv1 cells (two clones) when compared to CTL.Proteomic analysis highlighting proteins associated with the ER stress response significantly down‐regulated in THEM6 KO 22rv1 cells when compared to CTL (*P*‐value ≤ 0.05, FC = 1.3).Western blot analysis of BIP, XBP1s, ATF4, CHOP and THEM6 expression in CTL and THEM6 KO 22rv1 cells.Proliferation of CTL and THEM6 KO CRPC cells treated with tunicamycin (2.5 µg/ml) for 72 h. Cell count is normalised to initial number of cells at T0.Proteomic analysis highlighting ATF4 targets significantly down‐regulated in THEM6 KO LNCaP AI cells when compared to CTL (*P*‐value ≤ 0.05, FC = 1.3).Western blot analysis of ATF4 and THEM6 expression in CTL and THEM6 KO LNCaP AI cells.Western blot analysis of ATF4, CHOP and THEM6 expression in PCa cells following THEM6 silencing.Differential expression of EIF4G1 and ASNS in high and low THEM6 tumours according to the PRAD TCGA dataset.Western blot analysis of ATF4 and CHOP expression in CTL and THEM6 KO 22rv1 cells treated with palmitic acid (200 µM) for 48 h.Western blot analysis of ATF4 and CHOP expression in CTL and THEM6 KO LNCaP AI cells treated with palmitic acid (200 µM) or hexadecylglycerol (50 µM) for 48 h. Data information: Panels (C, F, G, I, J) HSC70 was used as a sample loading control. Panels (B, D, E) Data are presented as mean values ± SD. Panel (H) Centre line corresponds to median of data, top and bottom of box correspond to 90^th^ and 10^th^ percentile, respectively. Whiskers extend to adjacent values (minimum and maximum data points not considered outliers). Statistical analysis: (D) One‐way ANOVA with a Dunnett's multiple comparisons test. (H) two‐tailed Mann–Whitney *U*‐test. Data reproducibility: Panels (C, F, G, I, J) representative image from three independent biological experiments. Panels (B, D, E) *n* = 3 independent biological experiments. Panel (H) *n* = 225 tumours for THEM6 low and *n* = 121 tumours for THEM6 high. Source data are available online for this figure.

Perturbation of ER homeostasis results in the activation of a tightly regulated stress response programme, the unfolded protein response (UPR), in order to rapidly alleviate ER stress (Hetz *et al*, 2020). Enrichment pathway analysis of the THEM6‐deficient 22rv1 cells highlighted “Response to ER stress” as the main pathway regulated in absence of THEM6 (Fig 5A), with 17 proteins referenced in this pathway significantly down‐regulated in both KO clones (FC = 1.3, *P* < 0.05, Fig 5B and Table EV1). Interestingly, BIP (HSPA5), the main regulator of the UPR, was identified among the proteins significantly down‐regulated in 22rv1 THEM6 KO cells (Fig 5B), and we confirmed this result by WB (Fig 5C). Impaired UPR activation in THEM6‐deficient cells was further evidenced by decreased levels of the UPR effectors XBP1s (spliced isoform of XBP1), ATF4 and CHOP (DDIT3; Fig 5C). As a consequence, THEM6 KO significantly sensitised CRPC cells to prolonged ER stress, caused by chronic tunicamycin treatment (Fig 5D).

**Figure EV5:**
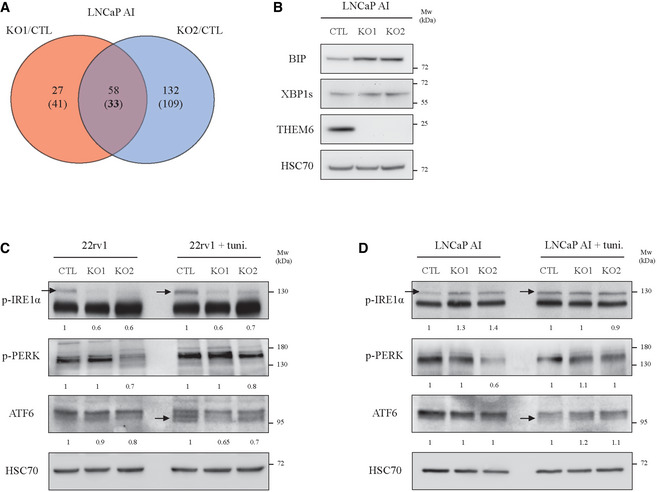
THEM6 regulates UPR activation in CRPC Venn diagram highlighting commonly modulated proteins (*P*‐value ≤ 0.05, FC = 1.3) in THEM6 KO LNCaP AI cells (two clones) when compared to CTL. Up‐regulated proteins are on top; Down‐regulated proteins are into brackets.Western blot analysis of BIP, XBP1s and THEM6 expression in CTL and THEM6 KO LNCaP AI cells.Western blot analysis of p‐IRE1α, p‐PERK and ATF6 expression in CTL and THEM6 KO 22rv1 cells treated or not with tunicamycin.Western blot analysis of p‐IRE1α, p‐PERK and ATF6 expression in CTL and THEM6 KO LNCaP AI cells treated or not with tunicamycin. Data information: Panels (B, C, D) HSC70 was used as a sample loading control. Data reproducibility: (B, C, D) representative image from three independent biological experiments. Source data are available online for this figure.

Proteomic analysis of THEM6 KO in LNCaP AI cells also highlighted ER perturbation as the main consequence of THEM6 depletion (Fig EV5A and Table EV4). Of note, THEM6 KO in this cell type led to an accumulation of several ER chaperones, including BIP, and did not consistently affect XBP1s levels (Fig EV5B). In contrast with the IRE1a‐XBP1s signalling, the proteomic analysis highlighted many ATF4 targets that were down‐regulated in LNCaP AI THEM6 KO cells (Fig 5E), suggesting that THEM6 might be particularly important for ATF4 activation in PCa. In line with this idea, we found that ATF4 levels were strongly reduced in THEM6‐deficient LNCaP AI cells (Fig 5F) and that both ATF4 and CHOP were also decreased following transient THEM6 silencing in multiple PCa cell lines (Fig 5G). In addition, analysis of the PRAD TCGA data revealed that EIF4G1, a member of the EIF4F complex required for ER stress‐induced translation of ATF4, and ASNS, a canonical ATF4 target, were significantly enriched in tumours with high THEM6 expression (Fig 5H and Table EV3), thus underpinning the importance of THEM6 in the establishment of the ATF4‐mediated stress response.

Upon ER stress, activation of ATF4 is canonically regulated by the PERK‐eiF2α pathway (Chevet *et al*, 2015). In order to understand how THEM6 loss could affect ATF4 expression, we assessed the activation of the three branches of the UPR (Wang & Kaufman, 2014). We evaluated the phosphorylation levels of PERK and IRE1α, and the proteolytic processing of ATF‐6 in CTL and THEM6 KO cells treated with tunicamycin (Fig EV5C and D). Surprisingly, while 22rv1 THEM6 KO cells displayed decreased activation levels of IRE1α and ATF6 following tunicamycin treatment, we did not observe consistent changes in the phosphorylation levels of PERK (Fig EV5C). Moreover, tunicamycin‐induced activation of all three pathways was not affected in the LNCaP AI model (Fig EV5D), suggesting that THEM6 loss might affect ATF4 activation in a non‐canonical way.

Lipid‐mediated stress also leads to UPR activation (Volmer & Ron, 2015). Moreover, in yeast, lipid‐mediated induction of the UPR results in the activation of a specific transcriptional programme that is distinct from the one induced by proteotoxic stress (Fun & Thibault, 2020). Because of the implication of THEM6 in maintaining lipid homeostasis (Figs 2 and 4), we wondered whether the inability of the THEM6‐deficient cells to induce ATF4 was due to changes in their lipid composition. While CTL cells strongly induced ATF4 expression following palmitic acid (PA) treatment, ATF4 levels remained unaffected by PA treatment in THEM6 KO cells (Fig 5I). Importantly, treatment with the ether lipid precursor hexadecylglycerol (HG) induced ATF4 expression to a similar level in both THEM6 KO and CTL cells (Fig 5J). Taken together, these results suggest that the stress‐induced activation of ATF4 depends, at least in part, on THEM6‐mediated lipid remodelling in CRPC.

### High THEM6 expression is associated with poor clinical outcome and correlates with high levels of UPR activation in PCa patients

**Figure 6:**
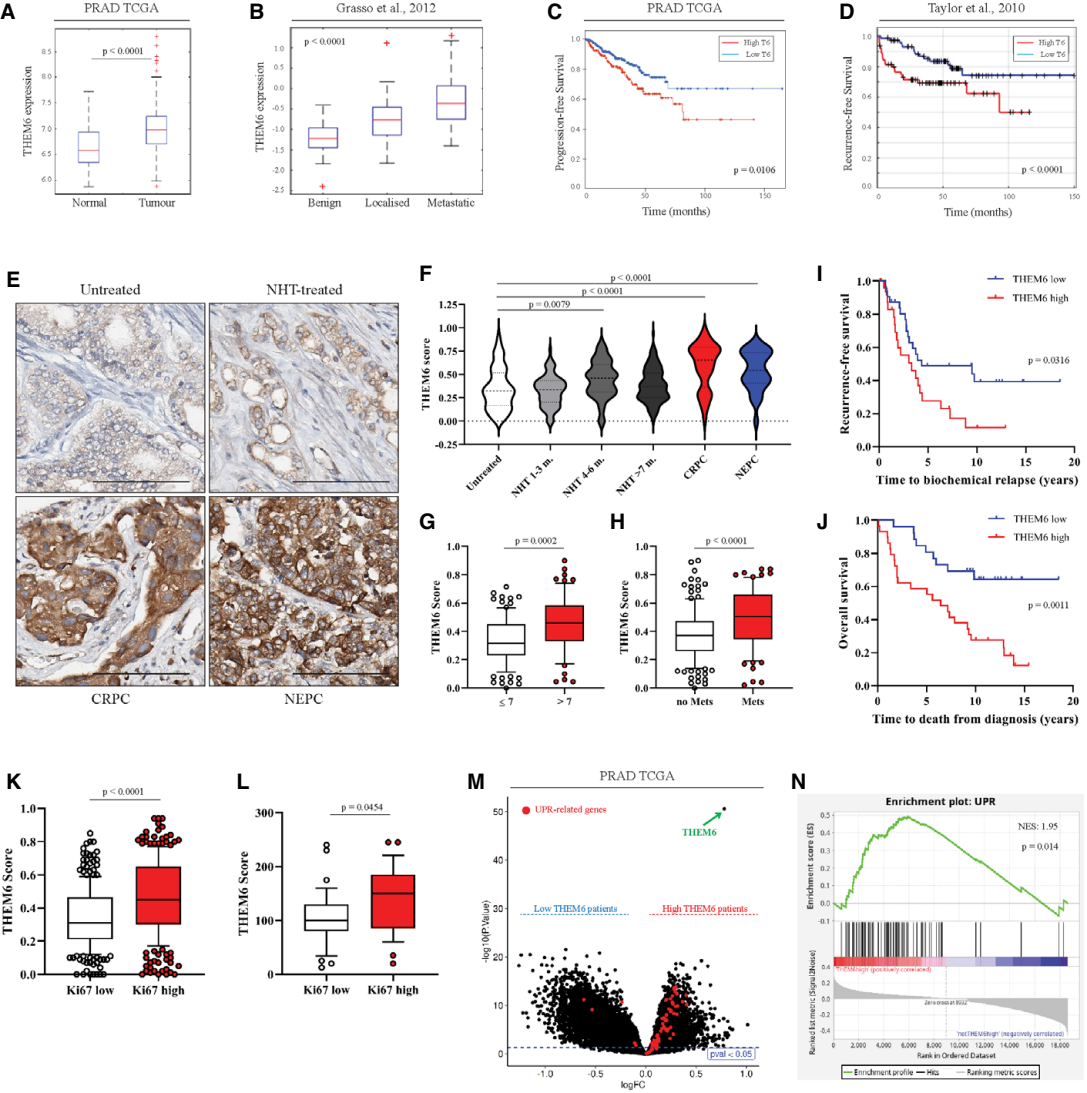
THEM6 is a clinically relevant target in CRPC AGene expression analysis of THEM6/c8orf55 in normal and tumoural prostate tissues according to the PRAD TCGA dataset (*n* = 489).BGene expression analysis of THEM6/c8orf55 in benign, localised and metastatic tumoural prostate tissues according to the GSE35988 dataset (*n* = 122).CKaplan–Meier progression‐free survival analysis of PCa patients stratified according high and low THEM6 expression using the PRAD TCGA dataset.DKaplan–Meier recurrence‐free survival analysis of PCa patients stratified according to median THEM6 expression using the GSE21034 dataset (*n* = 179).EIHC staining of THEM6 expression in treatment naïve, NHT‐treated, CRPC and NEPC tumours. Scale bar represents 100 µm.FQuantification of THEM6 staining in PCa tissue samples.G, HQuantification of THEM6 expression in PCa tissue samples according to Gleason score (G) and metastatic status (H) in the patients analysed in (F).I, JKaplan–Meier recurrence‐free (I) and overall (J) survival analysis of PCa patients stratified according to median THEM6 expression.KQuantification of THEM6 expression in PCa tissue samples according to Ki67 expression in the patients analysed in (F).LQuantification of THEM6 expression in PCa tissue samples according to Ki67 expression in the patients analysed in (I, J).MVolcano plot of the differentially modulated genes in PCa patients from the PRAD TCGA stratified according to THEM6 expression. Red dots represent the UPR‐related genes extracted from the UPR‐gene signature obtained from (Adamson *et al*, 2016).NGene set enrichment plot analysed from PCa patients of the PRAD TCGA dataset using the UPR gene signature obtained from (Adamson *et al*, 2016). Data information: Panels (A, B) Center line corresponds to median of data, top and bottom of box correspond to 75^th^ and 25^th^ percentile, respectively. Whiskers extend to adjacent values (minimum and maximum data points not considered outliers). Panel (F) Centre line corresponds to median of data, top and bottom lines correspond to upper and lower quartiles. Panels (G, H, K, L) Centre line corresponds to median of data, top and bottom of box correspond to 90^th^ and 10^th^ percentile, respectively. Whiskers extend to adjacent values (minimum and maximum data points not considered outliers). Statistical analysis: (A, B) pairwise ANOVA. (C, D, I, J) logrank test. (F) One‐way ANOVA with a Dunnett's multiple comparisons test. (G, H, K, L) two‐tailed Mann–Whitney *U*‐test. Data reproducibility: Panels (A, C) *n* = 489 tumours. Panel (B) *n* = 122 tumours. Panel (D) *n* = 179. Panels (E, F) *n* = 132; 90; 66; 137; 44; 30 tumours for Untreated; NHT 1–3; NHT4–6; NHT > 7; CRPC; NEPC respectively. Panel (G) *n* = 99 tumours for Gleason score ≤ 7 and *n* = 63 tumours for Gleason score > 7. Panel (H) *n* = 151 tumours for non‐metastatic patients and *n* = 76 tumours for metastatic patients. Panels (I, J) *n* = 35 tumours for THEM6 low and *n* = 34 tumours for THEM6 high. Panel (K) *n* = 269 tumours for Ki67 low and *n* = 270 tumours for Ki67 high. (L) *n* = 35 tumours for Ki67 low and *n* = 29 tumours for Ki67 high. Panel (M) *n* = 489 tumours. Source data are available online for this figure.

**Figure EV6:**
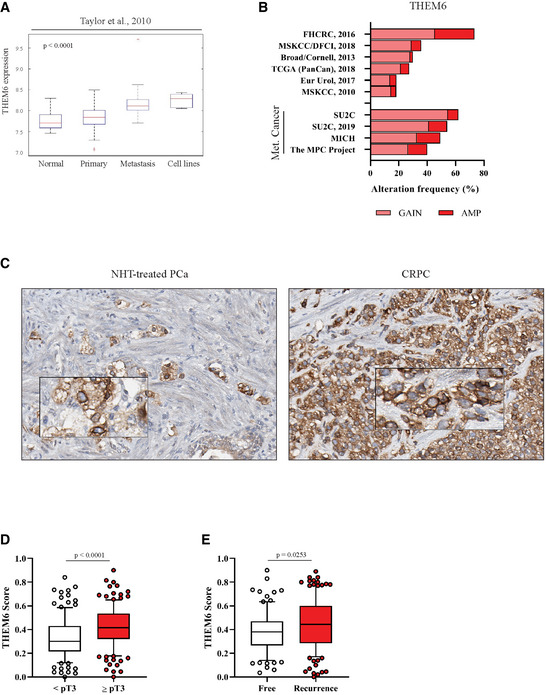
THEM6 is a clinically relevant target in CRPC AGene expression analysis of THEM6/c8orf55 in normal prostate tissues, primary tumours, distant metastases and PCa cell lines according to the GSE21034 dataset (*n* = 179).BPercentage of PCa patients showing genomic alteration (copy number gain or amplification) for *THEM6* in publicly available datasets of PCa. Analysis was performed using the cbioportal website https://www.cbioportal.org/.CHigh magnification pictures of THEM6 staining in NHT‐treated and CRPC tumours.D, EQuantification of THEM6 expression in PCa tissue samples according to T‐stage (D) and cancer recurrence (E). Data information: Panel (A) Center line corresponds to median of data, top and bottom of box correspond to 75^th^ and 25^th^ percentile, respectively. Whiskers extend to adjacent values (minimum and maximum data points not considered outliers). Panels (D, E) Center line corresponds to median of data, top and bottom of box correspond to 90^th^ and 10^th^ percentile, respectively. Whiskers extend to adjacent values (minimum and maximum data points not considered outliers). Statistical analysis: (A) pairwise ANOVA. (D, E) two‐tailed Mann–Whitney *U*‐test. Data reproducibility: (D) *n* = 129 tumours for T stage score < 3 and *n* = 135 tumours for T stage score ≥ 3. (E) *n* = 91 for disease‐free patients and *n* = 136 for recurred patients. Source data are available online for this figure.

Finally, we specifically assessed the clinical relevance of THEM6 in PCa. For this purpose, we first examined THEM6/c8orf55 expression in three publicly available datasets of localised and metastatic PCa (Figs 6A–D and EV6A and B). Analysis of the PRAD TCGA data revealed that *THEM6* mRNA expression was significantly higher in tumour tissue than in normal prostate epithelium (Fig 6A). Similarly, *THEM6* levels gradually increased from benign to localised and metastatic PCa lesions (Grasso *et al*, 2012) (Fig 6B) and were also elevated in distant metastases (Taylor *et al*, 2010) (Fig EV6A). In addition to high mRNA levels, we observed that *THEM6* was frequently amplified at the genomic level in PCa patients (average genomic alteration frequency of 34%, Fig EV6B). Of note, the incidence of *THEM6* amplification was even higher in metastatic cases (average genomic alteration frequency of 51%, Fig EV6B). Importantly, PCa patients presenting with high THEM6 expression consistently displayed shortened progression‐free and recurrence‐free survival in both the PRAD TCGA and the MSKCC (Taylor *et al*, 2010) cohorts, respectively (Fig 6C and D). To further demonstrate the importance of THEM6 in clinical PCa, we performed IHC on a tissue microarray (TMA, *n* = 297, Table EV5) comprised of tumours obtained from treatment‐naïve (Untreated), treatment‐responsive (neoadjuvant hormonal therapy, NHT‐treated), treatment‐resistant (CRPC) and neuroendocrine (NEPC) PCa patients (Figs 6E and EV6C). CRPC and NEPC tumours exhibited significantly higher levels of the THEM6 protein than untreated tumours, with the highest score obtained for CRPC (Fig 6F). High THEM6 levels significantly correlated with high Gleason score and high T‐stage tumours (Figs 6G and EV6D) and were also associated with the presence of metastases and cancer recurrence (Figs 6H and EV6E). We further validated THEM6 as a prognostic factor in PCa by staining a second TMA composed of tumour biopsies from treatment‐naïve patients documented with a 20‐year follow‐up (*n* = 69, Table EV5). In this second cohort, high THEM6 protein expression was strongly associated with poor patient survival (recurrence‐free and overall survival, Fig 6I and J). Moreover, in both patient cohorts, high THEM6 levels were significantly associated with high Ki67 expression (Fig 6K and L), indicating that THEM6 is expressed at higher levels in highly proliferative tumours. Finally, based on our *in vitro* data, we hypothesised that high levels of THEM6 could sustain UPR activation in PCa patients. To test this hypothesis, we took advantage of a recently defined UPR‐gene signature (Adamson *et al*, 2016) and evaluated the enrichment of this signature in two groups of patients stratified according to high and low THEM6 expression (PRAD TCGA dataset). Strikingly, most of the UPR‐related genes were significantly enriched in high THEM6 tumours (Fig 6M). Consequently, we found that the UPR‐gene signature was positively enriched in high THEM6 PCa patients (NES = 1.95, *P*‐value = 0.014, Fig 6N). Altogether, these results indicate that high THEM6 expression correlates with poor survival and high levels of UPR activation in PCa patients.

## Discussion

The remarkable reliance of PCa on AR signalling led to the generation of effective targeted therapies, such as enzalutamide, abiraterone acetate and other small molecule inhibitors of the AR pathway, which have significantly improved the clinical management of non‐resectable prostate tumours. However, resistance to such therapies ultimately results in the development of lethal CRPC. A detailed characterisation of the molecular signalling pathways associated with treatment resistance will therefore identify novel actionable targets and foster innovative therapeutic options. For example, a better understanding of treatment‐induced metabolic rewiring (Blomme *et al*, 2020) or specific dependencies on stress response pathways (Jin & Saatcioglu, 2020) might uncover vulnerabilities with the potential to synergise with current treatments. Our laboratory recently engaged in a comparison of different *in vivo* models of ADT resistance at multi‐omics levels (Martinez *et al*, 2021). From this comprehensive dataset, we identified thioesterase superfamily member 6 (THEM6) as a clinically relevant protein overexpressed in CRPC. In patients, THEM6 expression correlated with tumour aggressiveness and was highest in treatment‐resistant tumours. Furthermore, high THEM6 expression was associated with poor clinical tumour parameters, such as enhanced tumour proliferation and metastatic dissemination, and correlated with shortened overall and disease‐free patient survival. THEM6, formerly c8orf55, was originally discovered as a potential biomarker for colon cancer in a proteomic‐based analysis of human samples. The authors further validated THEM6 overexpression in several tumour types, including prostate (Kume *et al*, 2014). THEM6 has also been reported in additional proteomic studies, mainly comparing tumoural and respective non‐malignant tissues from various origins (Brentnall *et al*, 2009; Gamez‐Pozo *et al*, 2017).

Despite no characterised biological function, THEM6 has recently been classified in the THEM thioesterase superfamily due to the presence of a “HotDog” domain, an evolutionarily conserved domain with a predicted thioesterase activity (Pidugu *et al*, 2009). Members of the THEM superfamily (THEM1/ACOT11, THEM2/ACOT13, THEM4 and THEM5) display an acyl‐CoA thioesterase activity, although presenting differences in specificity towards their fatty acyl‐CoA substrates. Consequently, the THEM proteins have been described to play various roles in FA and lipid metabolism (Tillander *et al*, 2017). In line with these studies, we found that THEM6 depletion significantly altered the lipid composition of cancer cells. In particular, loss of THEM6 resulted in consistent decreases in the levels of several ether lipid classes (ether TGs and ether PCs). Despite being relatively understudied in this context, ether lipids are frequently over‐represented in tumours (Fontaine *et al*, 2020) and support a pro‐oncogenic phenotype (Benjamin *et al*, 2013). Ether lipids are a subset of glycerolipids characterised by the presence of an ether‐alkyl or a vinyl ether‐alkyl (plasmalogens) bond at the glycerol sn‐1 position. Their synthesis is initiated in peroxisomes and terminates in the ER, where they undergo active acyl chain remodelling (Phuyal *et al*, 2015). In addition to their roles in redox homeostasis (Engelmann, 2004; Zou *et al*, 2020) and cellular signalling, ether lipids are essential components of biological membranes. The importance of ether lipids in membrane protein trafficking, as well as their coordinate regulation with sphingolipids, has recently been described in mammalian cells (Jiménez‐Rojo *et al*, 2020). Interestingly, we identified THEM6 as an ER membrane protein whose absence primarily impacts ER function and morphology. Indeed, THEM6‐deficient cells displayed abnormal ER structure with the presence of highly dilated lumen, a phenotype that is also observed following dysregulation of ether lipid metabolism (Thai *et al*, 2001). The observation that both mitochondrial and lysosomal structures were also affected in THEM6 KO cells further suggests that THEM6 loss induces a global perturbation of membrane homeostasis. Moreover, THEM6 closely interacted with many membrane proteins involved in protein transport (exportins, importins, transportins, components of the ERAD machinery and the OST complex), and THEM6 silencing led to a selective down‐regulation of the membrane‐associated chaperone calnexin, without affecting its soluble homolog calreticulin. CALX and CALR share substantial sequence identity, but the former is a type 1 transmembrane protein, while the second mainly resides in the ER lumen (Williams, 2006). Of note, this decrease in CALX levels was much more pronounced upon transient siRNA transfection than following CRISPR‐mediated KO, suggesting that THEM6‐deficient cells might have adapted to compensate for this effect. Taken together, these results suggest that THEM6 is involved in the regulation of membrane homeostasis and trafficking. An appealing hypothesis would be that THEM6 influences membrane properties by controlling the balance between ester‐ and ether‐bound lipids within the ER, for example by regulating the pool of FA that must be incorporated into ether lipids. The fact that THEM6 is the only member of the THEM superfamily presenting with a transmembrane domain supports this hypothesis, but at the same time renders the purification and structural analysis of the THEM6 protein very challenging. Therefore, additional work remains to be done to fully characterise THEM6 enzymatic activity and uncover its biochemical substrates.

The ER is essential for the maintenance of both lipid and protein homeostasis. Perturbation of ER homeostasis, induced by increased proteo‐ or lipotoxicity, activates a complex signalling network, called the UPR, which aims at rapidly reducing ER stress (Hetz *et al*, 2020). Importantly, we demonstrate that THEM6 is required for a correct activation of the UPR. In particular, the inability of cancer cells to activate the ATF4/CHOP pathway was a constant feature of THEM6‐deficient PCa cancer cells, irrespective of their AR status. ATF4 has recently been highlighted as an important regulator of PCa growth and survival (Pallmann *et al*, 2019). While the molecular mechanisms involved in the THEM6‐mediated control of ATF4 remain to be fully uncovered, we showed that THEM6‐depleted cells were unable to respond to palmitate‐induced ER stress but managed to activate the UPR in response to hexadecylglycerol treatment, a precursor of the ether lipid synthesis. This result not only demonstrates the implication of THEM6 in ether lipid metabolism but also highlights its importance in the establishment of the lipid‐mediated stress response. Importantly, failure to activate the UPR in response to ER stress has also been reported in the context of THEM1 and THEM2 deficiencies. Indeed, genetic ablation of *Them1* in mice attenuated diet‐ and chemical‐induced ER stress responses, as well as UPR‐mediated lipogenesis (Zhang *et al*, 2012). Of note, the authors also speculated that *Them1* could influence ER stress levels by modulating the phospholipid composition of the ER membrane. Similarly, an elegant study from the same group demonstrated that *Them2*‐mediated trafficking of saturated fatty acid critically regulates ER membrane fluidity and is therefore required for calcium‐dependent induction of ER stress in non‐physiological condition (Ersoy *et al*, 2018).

Endoplasmic reticulum stress induction and subsequent activation of the UPR have been described to promote tumorigenesis and treatment resistance in cancer (Chevet *et al*, 2015). In PCa, resistance to ADT involves the activation of a sustained stress response together with major metabolic reprogramming (Bader & McGuire, 2020; Blomme *et al*, 2020). As such, therapeutic targeting of the UPR has emerged as a promising strategy for the treatment of advanced PCa (Nguyen *et al*, 2018; Sheng *et al*, 2019; Jin & Saatcioglu, 2020). Importantly, we demonstrate that high THEM6 expression significantly correlates with high levels of UPR activation in PCa patients. These data suggest that THEM6 overexpression following ADT could facilitate UPR activation and allow cancer cells to survive therapy‐induced ER stress. Therefore, developing small molecule inhibitors specifically targeting THEM6 enzymatic activity might represent a promising therapeutic approach to develop adjuvant therapy to ADT. Furthermore, patient stratification according to the levels of tumoural THEM6 could be used to delineate subsets of patients that would benefit from UPR pharmacological inhibition (Hetz *et al*, 2019). Overall, through its role in lipid remodelling and its ability to regulate stress‐induced UPR, THEM6 might provide an actionable target with the potential to be considered for the development of future anticancer therapies.

## Materials and Methods

### Cell culture

LNCaP, C4‐2, CWR22res, VCaP, DU‐145, PC‐3 and PC‐3met were cultured in RPMI (Gibco, Thermo Fisher Scientific, Waltham, MA, USA) medium supplemented with 10% foetal bovine serum (Gibco, Thermo Fisher Scientific, Waltham, MA, USA) and 2 mM glutamine (Gibco, Thermo Fisher Scientific, Waltham, MA, USA). 22rv1 and LNCaP AI cells were maintained in phenol‐free RPMI (Gibco, Thermo Fisher Scientific, Waltham, MA, USA) supplemented with 10% charcoal‐stripped serum (Gibco, Thermo Fisher Scientific, Waltham, MA, USA) and 2 mM glutamine. MCF‐7 were maintained in DMEM (Gibco, Thermo Fisher Scientific, Waltham, MA, USA) supplemented with 10% foetal bovine serum (Gibco, Thermo Fisher Scientific, Waltham, MA, USA) and 2 mM glutamine. CWR22Res cells (hormone‐responsive variant of CWR22 cells) were obtained from Case Western Reserve University, Cleveland, Ohio. All other cell lines were obtained from ATCC. All cells were maintained at 37°C under 5% CO_2_ and routinely harvested with trypsin. All cell lines were authenticated by STR DNA profiling and were tested negative for mycoplasma using the Mycoalert mycoplasma detection kit (Lonza, Basel, Switzerland). Cells were kept in culture for a maximum of 10 passages after recovery from frozen vials.

### Generation of CRISPR THEM6 KO cells

1 × 10^6^ cells were transfected with commercially available THEM6/DSCD75 KO or CTL plasmids (Santa Cruz Technologies, Dallas, TX, USA) using a nucleofector (Amaxa Biosystems, Lonza, Basel, Switzerland) according to the manufacturer's instructions. Clonal selection of THEM6 KO cells was performed using puromycin (Sigma‐Aldrich, St Louis, MI, USA). Protein depletion was confirmed using Western blot.

### Cell proliferation

1 × 10^6^ cells were seeded in 6‐well plates and allowed to attach overnight. The next day, cells were harvested with trypsin (T0) or allowed to grow for additional 72 h. Cells were then counted using a CASY cell counter (Roche, Basel, Switzerland). Final cell number was normalised to the initial cell count obtained at T0. Data are expressed as relative percentage of CTL cells.

### Measurement of cell stiffness using Atomic Force Microscopy

The mechanical properties of individual cells were measured using an Atomic Force Microscope Nanowizard II (JPK Instruments, Bruker, Berlin, Germany) with cell heater attachment mounted on an inverted optical microscope (Zeiss Observer Axio A.1, Zeiss, Cambridge, UK). Force indentation measurements were carried out as described previously (McPhee *et al*, 2010; Rudzka *et al*, 2019). Briefly, the AFM colloidal probes were prepared by gluing a 5.2‐µm spherical silica bead (Bangs Laboratories, Inc, Fishers, IN, USA) at the end of a tipless cantilever (Arrow‐TL2‐50 with a spring constant of 0.03 N/m, Nanoworld Innovative Technologies, Neuchatel, Switzerland) and calibrated prior to use. Cells were seeded on petri dishes at a concentration of 10^4^ cells cm^−2^ and cultured overnight. During the experiment, cells were kept at 37°C and in 25 mM HEPES buffered medium to maintain pH levels. Five points over the central nucleus area of each cell were measured, with ~ 50 cells per population taken from multiple dishes to ensure reproducibility. The Young's modulus was derived by fitting the extension part of a force–distance curve with the Hertzian spherical model using an in‐house R Script (Lin *et al*, 2007). GraphPad Prism 8.4.2 was used to produce graphs and perform statistical analysis of the data.

### Proteomic analysis

1 × 10^6^ cells were seeded in 6‐well plates and allowed to grow for 48 h. Cells were then lysed in SDS‐containing buffer (2% SDS, 50 mM triethylammonium bicarbonate, pH 8.5) and briefly sonicated and centrifuged at 16,000 *g* for 5 min at 4°C. Protein concentration was determined using BCA assay (Thermo Fisher Scientific, Waltham, MA, USA) and samples were stored at −80°C until further processing. For each experiment, 40 µg of protein extracts were reduced with 10 mM DTT and newly generated thiols were subsequently alkylated with 55 mM iodoacetamide. Alkylated proteins were precipitated using trichloroacetic acid (TCA). Washed pellets were reconstituted in 50 µl of 200 mM HEPES and digested first with Endoproteinase Lys‐C (ratio 1:33 enzyme:lysate, Alpha Laboratories, Eastleigh, UK) for 1 h, followed by an overnight trypsin digestion (ratio 1:33 enzyme:lysate, Promega, Madison, WI, USA).

The digested peptides from each experiment, and a pool sample, were differentially labelled using TMT10‐plex reagent (Thermo Fisher Scientific, Waltham, MA, USA). Each sample was labelled with 0.1 mg of TMT reagent dissolved in 50 μl of 100% anhydrous acetonitrile. The reaction was carried out at room temperature for 2 h. Fully labelled samples were mixed in equal amount and desalted using a Sep Pak C18 reverse phase solid‐phase extraction cartridges (Waters Corporation, Milford, MA, USA). After clean‐up, the TMT‐labelled peptides were fractionated using high pH reverse phase chromatography on a C18 column (150 × 2.1 mm i.d. ‐ Kinetex EVO [5 μm, 100 Å]) using an HPLC system (Agilent, LC 1260 Infinity II, Agilent, Santa Clara, CA, USA). A two‐step gradient was applied, from 1 to 28% B in 42 min, then from 28 to 46% B in 13 min to obtain a total of 21 fractions for MS analysis.

Pull‐down experiments were performed on Myc‐tagged THEM6 overexpressing cells (hereafter referred to as Affinity Purification Mass Spectrometry experiment, AP‐MS). Agarose beads were resuspended in a 2 M Urea and 100 mM ammonium bicarbonate buffer and stored at −20°C. Biological triplicates were digested with Lys‐C (Alpha Laboratories, Eastleigh, UK) and trypsin (Promega, Madison, WI, USA) on beads as previously described (Hubner *et al*, 2010). Prior mass spectrometry analysis, digested peptides were desalted using StageTip (Rappsilber *et al*, 2007).

Peptides from all experiments were separated by nanoscale C18 reverse‐phase liquid chromatography using an EASY‐nLC II 1200 (Thermo Fisher Scientific, Waltham, MA, USA), using a binary gradient with buffer A (2% acetonitrile) and B (80% acetonitrile), both containing 0.1% formic acid. Samples were loaded into fused silica emitter (New Objective) packed in‐house with ReproSil‐Pur C18‐AQ, 1.9 μm resin (Dr Maisch GmbH). Packed emitters were heated by means of a column oven (Sonation, Biberach, Germany) integrated into the nanoelectrospray ion source (Thermo Fisher Scientific, Waltham, MA, USA). An Active Background Ion Reduction Device (ABIRD, ESI Source Solutions, Woburn, MA, USA) was used to decrease air contaminants signal level. The Xcalibur software (Thermo Fisher Scientific, Waltham, MA, USA) was used for data acquisition.

An Orbitrap Fusion Lumos mass spectrometer (Thermo Fisher Scientific, Waltham, MA, USA) was used for the TMT‐labelled proteome analysis. Peptides were loaded into a packed 50 cm fused silica emitter kept at 50°C and eluted at a flow rate of 300 nl/min using different gradients optimised for three sets of fractions: 1–7, 8–15 and 16–21 as previously described (Cao *et al*, 2020). A full scan over mass range of 350–1,400 *m*/*z* was acquired at 60,000 resolution at 200 *m*/*z*, with a target value of 500,000 ions for a maximum injection time of 20 ms. Higher energy collisional dissociation fragmentation was performed on the 15 most intense ions, for a maximum injection time of 100 ms, or a target value of 100,000 ions. Peptide fragments were analysed in the Orbitrap at 50,000 resolution.

AP‐MS was carried out using an Orbitrap Q‐Exactive HF mass spectrometer (Thermo Fisher Scientific, Waltham, MA, USA) using 20 cm fused silica emitter kept at 35°C to separate the peptides over a 60‐min gradient at a flow rate of 300 nl/min. For the full scan, a resolution of 60,000 at 200 *m*/*z* was used to scan the mass range from 350 to 1,400 *m*/*z*. The top 10 most intense ions in the full MS were isolated for fragmentation with a target of 50,000 ions, for a maximum of 75 ms, at a resolution of 15,000 at 200 *m*/*z*.

The MS Raw data were processed with MaxQuant software (Cox & Mann, 2008) version 1.6.3.3 (TMT proteome experiments) or 1.5.5.1 (AP‐MS experiment) and searched with Andromeda search engine (Cox *et al*, 2011), querying SwissProt (UniProt Consortium, 2010) Homo sapiens (30/04/2019; 42,438 entries). First and main searches were performed with precursor mass tolerances of 20 and 4.5 ppm, respectively, and MS/MS tolerance of 20 ppm. Database was searched requiring specificity for trypsin cleavage and allowing maximum two missed cleavages. Methionine oxidation and N‐terminal acetylation were specified as variable modifications, and cysteine carbamidomethylation as fixed modification. The peptide, protein and site false discovery rate (FDR) was set to 1%.

For the TMT‐proteome analysis, MaxQuant was set to quantify on “Reporter ion MS2”, and TMT10plex was chose as Isobaric label. Interference between TMT channels was corrected by MaxQuant using the correction factors provided by the manufacturer. The “Filter by PIF” option was activated and a “Reporter ion tolerance” of 0.003 Da was used.

Proteins identified in the AP‐MS experiment were quantified according to the label‐free quantification algorithm available in MaxQuant (Cox *et al*, 2014).

MaxQuant outputs were analysed with Perseus software version 1.6.2.3 (Tyanova *et al*, 2016). From the ProteinGroups.txt file, Reverse and Potential Contaminant flagged proteins were removed, as well as protein groups identified with no unique peptides. Only proteins quantified in three out of three replicate experiments were included in the analysis. To determine significant change in protein abundance, a Student *t*‐test with a 5% FDR (permutation‐based) was applied using the corrected reporter ions Intensities for the TMT proteome analysis or label‐free quantitation intensities for the AP‐MS experiment.

### Lipidomic analysis

Lipidomic analysis was performed according to Blomme *et al* (2020). Single‐phase lipid extraction was carried out using an extracting solution of methanol–butanol (1:1 ratio, BuMe), kept at 4°C and supplemented with an internal standard (Splash Lipidomix, Avanti Polar Lipids, Alabaster, AL, USA) further used as a quality control. Briefly, plates were washed twice with ice‐cold PBS and incubated with BuMe on dry ice for 20 min. Extracted lipids were then transferred to 1.5 ml tubes and centrifuged for 10 min at 14,000 rpm (4°C). Supernatants were collected and stored at −80°C prior to LC‐MS analysis. Protein pellets attached to the plate were left to dry overnight and quantified for subsequent normalisation.

Lipid analyses were performed using an LC‐MS system including an Ultimate 3000 HPLC (Thermo Fisher Scientific, Waltham, MA, USA) coupled to a Q‐Exactive Orbitrap mass spectrometer (Thermo Fisher Scientific, Waltham, MA, USA). Polar and non‐polar lipids were separated on an Acquity UPLC CSH C18 column (100 × 2.1 mm; 1.7 µm; Waters Corporation, Milford, MA, USA) maintained at 60°C. The mobile phases consisted of 60:40 ACN: H_2_O with 10 mM ammonium formate, 0.1% formic acid and 5 µM of phosphoric acid (A) and 90:10 IPA:ACN with 10 mM ammonium formate, 0.1% formic acid and 5 µM phosphoric acid (B). The gradient was as follows: 0–2 min 30% (B); 2–8 min 50% (B); 8–15 min 99% (B); 15–16 min 99% (B); and 16–17 min 30% (B). Sample temperature was maintained at 6°C in the autosampler and 5 µL of sample were injected into the LC‐MS instrument.

Thermo Q‐Exactive Orbitrap MS instrument was operated in both positive and negative polarities, using the following parameters: mass range 240–1,200 *m*/*z* (positive) and 240–1,600 (negative), spray voltage 3.8 kV (ESI^+^) and 3 kV (ESI^−^), sheath gas (nitrogen) flow rate 60 units, auxiliary gas (nitrogen) flow rate 25 units, capillary temperature (320°C), full scan MS1 mass resolving power 70,000. Data‐dependent fragmentation (dd‐MS/MS) parameters for each polarity are as follows: TopN: 10, resolution 17,500 units, maximum injection time: 25 ms, automatic gain control target: 5e^5^ and normalised collision energy of 20 and 25 (arbitrary units) in positive polarity. TopN: 5, resolution 17,500 units, maximum injection time: 80 ms automatic gain control target: 5e^5^ and normalised collision energy of 20 and 30 (arbitrary units) in negative polarity. The instrument was externally calibrated to <1 ppm using ESI positive and negative calibration solutions (Thermo Fisher Scientific, Waltham, MA, USA).

Feature detection and peak alignment from .Raw files were performed using Compound Discoverer 3.2 (Thermo Fisher Scientific, Waltham, MA, USA). Files were also converted to .mgf format using MSConvert software (ProteoWizard) and MS2 files were searched against the LipiDex_ULCFA database using LipiDex software (Hutchins *et al*, 2018).

Data pre‐processing, filtering and basic statistics were performed using Perseus software version 1.6.14.0 after importing the final results table from LipiDex. For statistical purposes, we only kept lipid ions where the number of features was present in at least 60% of the samples. GraphPad Prism 8.4.2 was used to produce heatmaps.

### GC‐MS‐based determination of ^13^C‐sterols and fatty acids

Fatty acids (FA) and sterol measurements were performed as previously described in (Blomme *et al*, 2020) and (McGregor *et al*, 2020), respectively. Briefly, cells were incubated for 72 h in glucose‐ and glutamine‐free RPMI (Gibco, Thermo Fisher Scientific, Waltham, MA, USA) supplemented with 10% CSS, 2 mM [U^13^C]‐glutamine and 10 mM [U^13^C]‐glucose (Cambridge Isotopes Laboratories, UK).

Fatty acids were extracted in a methanol–chloroform–PBS buffer (750 µl 1:1 v/v PBS: methanol and 500 µl chloroform) supplemented with 50 µl of 1 mg/ml methanolic butylated hydroxytoluene (BHT, Sigma‐Aldrich, St Louis, MI, USA) and 20 µl of 0.05 mg/ml 17:0 PC (Avanti Polar Lipids, Albaster, AL, USA), used as an internal standard. Samples were centrifuged at 10,000 *g* for 5 min before the lower chloroform layer was extracted and dried under N_2_. Samples were reconstituted in 90 µl chloroform and incubated with 10 µl MethPrepII (Thermo Fisher Scientific, Waltham, MA, USA) for 20 min at room temperature.

Sterols were extracted in a methanol extraction buffer (1:9 v/v water: methanol) supplemented with 20 μl of lathosterol (100 ng/μl) internal standard. Saponification was performed by heating samples for 60 min at 80°C with 75 μl of 10 mol/l NaOH to obtain the total cholesterol pool. Upon cooling to room temperature, 200 μl water and 500 μl *n*‐hexane were sequentially added. Samples were then vortexed and the upper hexane layer was transferred to an autosampler vial. The *n*‐hexane extraction was repeated and samples dried under N_2_. Samples were reconstituted in 50 μl dry pyridine and 50 μl *N*‐Methyl‐*N*‐(trimethylsilyl) trifluoroacetamide (MSTFA, Sigma‐Aldrich, St Louis, MI, USA) silylation agent added. Samples were heated at 60°C for 60 min before cooling and immediate analysis by gas chromatography–mass spectroscopy (GC‐MS).

Fatty Acid Methyl Esters (FAMEs) and sterols were analysed using an Agilent 7890B GC system coupled to a 7000 Triple Quadrupole GC‐MS system, with a Phenomenex ZB‐1701 column (30 mm × 0.25 mm × 0.25 μm). For FAMEs, an initial temperature of 45°C was set to increase at 9°C/min, held for 5 min, then 240°C/min, held for 11.5 min, before reaching a final temperature of 280°C/min, held for 2 min. For cholesterol, the initial temperature was set at 200°C and increased at 20°C/min up to 280°C and held for 9 min. The instrument was operated in pulsed splitless mode in the electron impact mode, 50 eV, and mass ions were integrated for quantification using known standards to generate a standard curve. Palmitic, stearic and oleic acid peak areas were extracted using mass‐to‐charge ratios (*m*/*z*) 270, 298 and 296, respectively. Cholesterol peak areas were extracted from *m*/*z* 458. Mass Hunter B.06.00 software (Agilent) was used to quantify isotopomer peak areas before natural abundance isotope correction was performed using an in‐house algorithm.

Cholesterol was analysed using an Agilent 7890B GC system coupled to an Agilent 7000 Triple Quadrupole GC‐MS system, which was operating in a single quadrupole mode, with a Phenomenex ZB‐1701 column (30 mm × 0.25 mm × 0.25 μm). An initial temperature of 200°C was set to increase at 20°C/min up to 280°C and held for 9 min. The instrument was operated in splitless mode in the electron impact mode, 70eV, for quantification and 50eV for labelling experiments. Cholesterol was quantified and isotope labelling pattern analysed using Mass Hunter B.06.00 software (Agilent). Cholesterol and lathosterol internal standard peak areas were extracted from mass‐to‐charge ratio (*m*/*z*) 458 for both. Cholesterol was normalised to the internal standard, and a standard curve was used to quantify mg cholesterol per sample.

### Bioinformatics analysis

Gene expression data were downloaded from TCGA and the GEO website. TCGA RNASeqV2 data were shift log transformed; GSE35988 and GSE21034 data were log transformed, using mean of probes per gene. Expression values were grouped according to sample type and group distributions were plotted using the matlab routine boxplot with the bar indicating the median, the box spanning from the 25^th^ to the 75^th^ percentiles, and whiskers spanning 2.7\sigma. Outliers beyond that span are indicated in red. Significant difference between the groups was measured using overall ANOVA.

For survival analysis (GSE21034), gene expression data from human samples (excluding cell lines) was normalised as before, mean‐centred and clustered into three groups using kmeans. Kaplan–Meier survival curves for the groups were plotted using the matlab routine kmplot.

For gene set enrichment analysis (GSEA), genes from differential expression analysis were ranked according to log2FC and GSEA was carried out on ranked list using clusterProfiler v4.1.4 with fgsea v1.16.0 algorithm. The GSEA enrichment was done against the GOBP STEROL HOMEOSTASIS gene set obtained from the Molecular Signatures Database (MSigDB) (Liberzon *et al*, 2011) using msigdbr v7.4.1 package. Final GSEA plots were generated using modified version of gseaplot2 from enrichplot package.

HTSeq Counts from the PRAD‐TCGA were downloaded through the UCSC Xena browser and the patients were split into two groups based on THEM6 expression. Differential gene expression analysis was then conducted on the voom‐normalised counts using the Limma R package (Ritchie *et al*, 2015). The GSEA software was used to conduct gene set expression analysis between the two groups of patients using a custom gene set containing the UPR gene signature derived from (Adamson *et al*, 2016).

### Human prostate cancer orthografts

*In vivo* orthograft experiments were performed in accordance with the ARRIVE guidelines (Kilkenny *et al*, 2011) and approved by a local ethics committee (University of Glasgow) under the Project Licences P5EE22AEE and 70/8645 in full compliance with the UK Home Office regulations (UK Animals [Scientific Procedures] Act 1986) and EU directive. Mice were randomly allocated into experimental groups at the time of injection. Cell injections were performed blindly by the investigator (not knowing which cell suspension was prepared for the injection). For PCa cells, 10 × 10^6^ cells/mouse were suspended in a 1:1 solution of serum‐free medium and growth factor reduced Matrigel (Corning, NY, USA). 50 µl of cell suspension were injected orthotopically into the anterior prostate lobe of 10‐week‐old CD1‐*nude* male mice (Charles River Laboratories, UK). Orchidectomy was either performed at the time of injection (22rv1) or 3 weeks after cell implantation (CWR22res). Tumours were allowed to develop for 8 weeks. Tumour volume was measured weekly using a Vevo3100 ultrasound imaging system (Fujifilm Visualsonics, The Netherlands). For LNCaP AI cells, 10 × 10^6^ cells/mouse were suspended in 50 µl of a 1:1 solution of serum‐free medium and growth factor reduced Matrigel (Corning, NY, USA) and injected subcutaneously into the flank of 8‐week‐old CD1‐*nude* male mice (Charles River Laboratories, UK). Orchidectomy was performed at the time of injection. Tumours were allowed to grow for 8 weeks. Once the tumour was palpable, tumour volume was calliper measured once a week with volume calculated using the formula of an ellipsoid (4/3 × width/2 × length/2 × height/2). At the end of the experiment, tumours were collected, cut into pieces and either fixed in 10% formalin for histological procedures or snap‐frozen in liquid nitrogen.

### Raman Spectroscopy analysis

Raman spectra were acquired on a Renishaw inVia Raman microscope equipped with a 532 nm Nd:YAG laser giving a maximum power of 500 mW, 1,800 l/mm grating and a Nikon NIR Apo 60×/1.0 N.A. water dipping objective. Prior to Raman measurements, tissue sections were dewaxed in xylene (2 × 15 min), 100% ethanol (5 min), 95% EtOH (5 min) and 90% EtOH (5 min). A water dipping objective was used to map the tissue sections with a step size of 100 µm in *x* and *y*, with 1 s acquisition time, 100% laser power and a spectral centre of 3,000 cm^−1^.

For initial pre‐processing steps, Renishaw Wire 4.1 software was used. The inbuilt software functions were used to remove cosmic rays followed by baseline subtraction. Baseline was subtracted using the baseline subtraction intelligent fitting function (with an 11^th^ order polynomial fitting and noise tolerance set to 1.50, applied to the whole spectral dataset). Further data analysis steps were then performed using custom MATLAB^®^ scripts. Outlier spectra which gave high intensity due to saturation or fluorescence were removed using a threshold function. Spectra were cut to the region of interest between 2,800 and 3,020 cm^−1^. Spectra from tissue regions were then extracted (based on total spectral intensity), and all extracted spectra were min‐max scaled for comparison between conditions. For each map, the resultant spectral data set went through further quality control steps which involved firstly, excluding spectra with a total spectral intensity < 60,000 and then removing spectra out with one standard deviation of the mean. Spectra were then scaled to the peak at 2,933 cm^−1^, and spectral data sets for all CTL and THEM6 KO samples were combined. The average for each condition was plotted for comparison. The ratio of the intensities of the peaks at 2,845 and 2,935 cm^−1^ as well as the ratio of the intensities of the peaks at 2,880 and 2,935 cm^−1^ were determined for each spectral data point. GraphPad Prism 8.4.2 was used to produce graphs and perform statistical analysis of the data.

### Patient material and immunohistochemistry

This study was approved by the West of Scotland Research Ethics Committee (05/S0704/94) and the Chair of the University of British Columbia Clinical Research Ethics Board (UBC CREB number: H09‐01628). All patients involved in this study provided written informed consent and all experiments conformed to the principles set out in the WMA Declaration of Helsinki and the Department of Health and Human Services Belmont Report.

Immunohistochemical (IHC) staining for THEM6 was performed on 4 µm formalin‐fixed paraffin‐embedded sections which had previously been incubated at 60°C for 2 h. The IHC staining was performed on an Agilent Autostainer link 48 (Agilent, Santa Clara, CA, USA) or on a Ventana DISCOVERY Ultra (Ventana Medical Systems, Tucson, Arizona, USA). Briefly, formalin‐fixed paraffin‐embedded (FFPE) TMA sections were deparaffinised before undergoing antigen retrieval. Sections were then successively incubated with anti‐THEM6 antibody (rabbit, 1:5,000, ab121743, Abcam, Cambridge, UK) and respective secondary antibody before being detected using Liquid DAB (Agilent, Santa Clara, CA, USA) or UltraMap DAB anti‐Rb Detection Kit (Ventana Medical Systems, Tucson, Arizona, USA). Stained slides were scanned with Leica Aperio AT2 (Leica Microsystems, Concord, Ontario, Canada). The area of interest in the tumour images were delineated by pathologist. Positively stained cells were quantified with Aperio ImageScope (Leica Biosystems, Buffalo Grove, Illinois, USA). Statistical analysis of THEM6 expression in PCa patients was conducted using a one‐way ANOVA with a Dunnett's multiple comparisons test. Statistical analysis of patient survival in correlation to THEM6 expression was conducted using a logrank test. Patient information is provided in Table EV5.

### Electron microscopy

CTL and THEM6 KO 22rv1 cells were fixed for 2 h at 4°C in a solution composed of 2.5% glutaraldehyde in 0.1 M Sorensen's buffer (0.2 M NaH_2_PO_4_, 0.2 M Na_2_HPO_4_, pH 7.4). After several washes in the same buffer, the samples were post‐fixed for 60 min with 2% osmium tetroxide, washed in deionised water, dehydrated through graded ethanol (70, 95, and 100%) and embedded in epon for 48 h at 60°C. Ultrathin sections (700‐A thick) were obtained by means of an ultramicrotome (Reichert Ultracut E) equipped with a diamond knife. The ultrathin sections were mounted on palladium/copper grids coated with collodion and contrasted with uranyl acetate and lead citrate for 5 min each before being examined under a Jeol JEM1400 transmission electron microscope at 80 kV.

### siRNA transfection

70%‐confluent cells were transfected using Lipofectamine RNAimax (siRNA) or Lipofectamine 2000 (plasmid; Invitrogen, Thermo Fisher Scientific, Waltham, MA, USA) according to the manufacturer's protocol. ON‐TARGETplus smartpool siRNAs against *hTHEM6* (L‐020791‐01‐0005), *mThem6* (L‐057277‐01‐0005) as well as non‐targeting siRNA (D‐001810‐01‐20) were purchased from Dharmacon (Dharmacon, Horizon inspired cell solutions, Cambridge, UK). *THEM6* human‐tagged ORF clone overexpressing plasmid and respective control (RC201709 and PS100001, Origene, Rockville, MD, USA) were purchased from Origene. Protein and RNA extractions were performed 48 h after transfection.

### qPCR analysis

Total RNA was extracted from 80%‐confluent cells using the RNeasy Mini Kit (Qiagen, Hilden, Germany) with on‐column DNase digestion (RNase‐Free DNase Set, Qiagen, Hilden, Germany). 4 μg of RNA was then used for cDNA preparation using the High‐Capacity cDNA Reverse Transcription Kit (Thermo Fisher Scientific, Waltham, MA, USA). Real‐time PCR was performed using TaqMan Universal Master Mix (Thermo Fisher Scientific, Waltham, MA, USA) with primer‐appropriate Universal ProbeLibrary probes (Roche, Basel, Switzerland) and the ABI 7500 FAST qPCR system (Thermo Fisher Scientific, Waltham, MA, USA). *CASC3* gene was used as a normalising control. Gene expression is shown relative to control cell levels. Primers (Thermo Fisher Scientific, Waltham, MA, USA) used in this study are provided in Table EV6.

### Immunoblotting

20 µg of proteins extracted in SDS buffer (1% SDS supplemented with protease and phosphatase inhibitors) were loaded on to a 4–12% gradient SDS–PAGE gel (Invitrogen, Thermo Fisher Scientific, Waltham, MA, USA) and transferred to a PVDF membrane (GE Healthcare, Chicago, IL, USA). Membrane was sequentially probed overnight with primary antibodies (see Table EV7) diluted in 5% BSA‐TBST, and respective HRP‐conjugated secondary antibodies diluted in 5% milk‐TBST. Antigen revelation was performed using the ECL kit (GE Healthcare, Chicago, IL, USA), and images were acquired on a MyECL machine (Thermo Fisher Scientific, Waltham, MA, USA).

### Immunofluorescence

Cells seeded on glass coverslips were fixed in ice‐cold Methanol/Acetone buffer, washed, blocked in 5% BSA and probed with primary antibodies overnight (anti‐THEM6, ab121743, anti‐CALR, ab22683, Abcam, Cambridge, UK; anti‐mitochondria, MAB1273, Merck Millipore, Burlington, MA, USA). The next day, coverslips were washed and incubated with fluorophore‐coupled secondary antibodies (Abcam, Cambridge, UK). Coverslips were mounted using Diamond Prolong with DAPI (Thermo Fisher Scientific, Waltham, MA, USA). Pictures were taken on a Nikon A1R confocal microscope (Nikon Instruments Europe B.V., Amsterdam, The Netherlands).

### Statistical analysis

Statistical analyses were performed using GraphPad PRISM software v8.4.2 (GraphPad Software Inc, San Diego, CA, USA).

## Author contributions

**Arnaud Blomme:** Conceptualization; Data curation; Formal analysis; Investigation; Methodology; Writing – original draft; Project administration; Writing – review & editing. **Coralie Peter:** Investigation. **Ernest Mui:** Investigation. **Giovanny Rodriguez Blanco:** Formal analysis; Methodology. **Ning An:** Investigation. **Louise M Mason:** Investigation; Methodology. **Lauren E Jamieson:** Formal analysis; Methodology. **Grace H McGregor:** Formal analysis; Methodology. **Sergio Lilla:** Formal analysis; Investigation; Methodology. **Chara Ntala:** Formal analysis. **Rachana Patel:** Resources; Investigation; Methodology. **Marc Thiry:** Formal analysis; Investigation; Methodology. **Sonia H Y Kung:** Resources; Formal analysis. **Marine Leclercq:** Data curation; Formal analysis. **Catriona A Ford:** Investigation. **Linda Rushworth:** Investigation. **David J McGarry:** Investigation. **Susan Mason:** Methodology. **Peter Repiscak:** Data curation; Formal analysis. **Colin Nixon:** Methodology. **Mark Salji:** Resources. **Elke Markert:** Data curation; Formal analysis. **Gillian MacKay:** Methodology. **Jurre J Kamphorst:** Supervision. **Duncan Graham:** Supervision; Methodology. **Karen Faulds:** Supervision; Methodology. **Ladan Fazli:** Resources; Formal analysis. **Martin E Gleave:** Resources. **Edward Avezov:** Resources. **Joanne Edwards:** Resources; Formal analysis. **Huabing Yin:** Supervision; Methodology. **David Sumpton:** Formal analysis; Investigation; Methodology. **Karen Blyth:** Resources; Supervision. **Pierre Close:** Supervision. **Daniel J Murphy:** Resources; Supervision. **Sara Zanivan:** Supervision; Methodology. **Hing Y Leung:** Conceptualization; Funding acquisition; Project administration; Writing – review & editing.

In addition to the CRediT author contributions listed above, the contributions in detail are:

AB and HYL designed the study. AB, CP, EMu, GRB, NA, LMM, LEJ, GHM, SL, MT, SHYK, CAF, LKR, DJMc, SM, CNi, MJS performed the experiments. AB, GRB, LMM, LEJ, GHM, SL, CNt, MT, SHYK, ML, PR, EMa, GMM, JE, DS analysed the data. AB, RP, MT, JJK, DG, KF, LF, MEG, EA, JE, HY, KB, PC, DJMu, SZ and HYL interpreted and discussed the data. AB and HYL wrote the manuscript. All authors critically reviewed the manuscript.

## Supporting information



Appendix

Expanded View Figures PDF

Table EV1

Table EV2

Table EV3

Table EV4

Table EV5

Table EV6

Table EV7

Source Data for Expanded View

Source Data for Figure 1

Source Data for Figure 2

Source Data for Figure 3

Source Data for Figure 4

Source Data for Figure 5

Source Data for Figure 6

## Data Availability

For proteomics, the raw files and the MaxQuant search results files have been deposited as partial submission to the ProteomeXchange Consortium via the PRIDE partner repository (Perez‐Riverol *et al*, 2019). Data are available with identifiers PXD024407 (http://www.ebi.ac.uk/pride/archive/projects/PXD024407), PXD024433 (http://www.ebi.ac.uk/pride/archive/projects/PXD024433) and PXD024456 (http://www.ebi.ac.uk/pride/archive/projects/PXD024456). For lipidomics, the raw LC‐MS/MS files have been deposited on the Metabolomics Workbench repository (Sud *et al*, 2016). Data are available via the following link: http://dx.doi.org/10.21228/M84M70. GSEA code is available on https://github.com/prepiscak/them6_gsea.
