# Supplementary material for: THEM6‐mediated reprogramming of lipid metabolism supports treatment resistance in prostate cancer
Source: EMBO Mol Med. 2022 Jan 11;14(3):e14764. doi: 10.15252/emmm.202114764 (PMC8899912; doi:10.15252/emmm.202114764)
Supplement: Supplementary file 1 — Appendix [file EMMM-14-e14764-s003.pdf]

Appendix

Appendix Table S1

**Appendix Table S1:** Exact p-values for Figures 1G, 1H, 1I, 2B, 2C, 2E, 4E, 4K, EV4H	
Fig. 1G	Orthografts - 22rv1 THEM6 KO<br><br>two-tailed Mann-Whitney U test<br><br>KO/CTL<br>Week 3p = 0.0079<br>Week 4p = 0.0079<br>Week 5p = 0.0079<br>Week 6p = 0.0079<br>Week 7p = 0.0317
Fig. 1H	Xenografts - LNCaP AI THEM6 KO<br><br>two-tailed Mann-Whitney U test<br><br>KO/CTL<br>Week 3n.s.<br>Week 4n.s.<br>Week 5n.s.<br>Week 6p = 0.0175<br>Week 7p = 0.0041
Fig. 1I	Orthografts - CWR22res THEM6 KO<br><br>Kruskal-Wallis test<br><br>KO1/CTLKO2/CTL<br>Week 3n.s.n.s.<br>Week 5n.s.n.s.<br>Week 6n.s.p = 0.0324<br>Week 7p = 0.0357p = 0.0265
Fig. 2B	Total lipids - 22rv1 THEM6 KO<br><br>1-way ANOVA - Dunnett's multiple comparison test<br><br>KO1/CTLKO2/CTL<br>DAGp = 0.0451n.s.<br>TGp < 0.0001p < 0.0001<br>PCn.s.n.s.<br>PEp = 0.0001p = 0.0048<br>PIn.s.n.s.<br>LysoPCn.s.n.s.<br>LysoPEn.s.p = 0.0084<br>Ether TGp < 0.0001p = 0.0002<br>Ether PCn.s.n.s.<br>Ether PEn.s.p < 0.0001<br>Cer.p = 0.0007p = 0.0027<br>SMn.s.n.s.<br>CEn.s.p = 0.0003
Fig. 2C	Total lipids - LNCaP AI THEM6 KO<br><br>1-way ANOVA - Dunnett's multiple comparison test<br><br>KO1/CTLKO2/CTL<br>DAGp = 0.0005p = 0.0464<br>TGp = 0.0010n.s.<br>PCn.s.n.s.<br>PEn.s.n.s.<br>PIn.s.n.s.<br>LysoPCn.s.n.s.<br>LysoPEp = 0.0004p = 0.0118<br>Ether TGp = 0.0020p = 0.0225<br>Ether PCp = 0.0005p = 0.0034<br>Ether PEn.s.n.s.<br>Cer.p = 0.0003n.s.<br>SMn.s.n.s.<br>CEn.s.n.s.
Fig. 2E	Total lipids - CWR22res THEM6 OE<br><br>two-tailed Student t-test<br><br>OE/EV<br>DAGp = 0.0002<br>TGp = 0.0009<br>PCn.s.<br>PEp = 0.0043<br>PIp < 0.0001<br>LysoPCn.s.<br>LysoPEn.s.<br>Ether TGp = 0.0341<br>Ether PCp = 0.0013<br>Ether PEp = 0.0030<br>Cer.p = 0.0188<br>SMp = 0.0001<br>CEn.s.

Fig. 4E	Desmosterol<br><br>1-way ANOVA - Dunnett's multiple comparison test<br><br>KO1/CTLKO2/CTL<br>m/z 0p = 0.0002p < 0.0001<br>m/z +1n.s.n.s.<br>m/z +2n.s.n.s.<br>m/z +3n.s.n.s.<br>m/z +4n.s.n.s.<br>m/z +5p= 0.0004p = 0.0002<br>m/z +6p = 0.0096p = 0.0017<br>m/z +7p = 0.0007p = 0.0001<br>m/z +8p = 0.0029p = 0.0013<br>m/z +9p < 0.0001p < 0.0001<br>m/z +10p = 0.0099p = 0.0026<br>m/z +11p = 0.0022p = 0.0033<br>m/z +12n.s.p = 0.0325<br>m/z +13n.s.n.s.<br>m/z +14n.s.p = 0.0160<br>m/z +15p = 0.0027p = 0.0010<br>m/z +16p = 0.0002p < 0.0001<br>m/z +17p < 0.0001p < 0.0001<br>m/z +18p < 0.0001p < 0.0001<br>m/z +19p < 0.0001p < 0.0001<br>m/z +20p < 0.0001p < 0.0001<br>m/z +21p < 0.0001p < 0.0001<br>m/z +22p < 0.0001p < 0.0001<br>m/z +23p < 0.0001p < 0.0001<br>m/z +24p < 0.0001p < 0.0001<br>m/z +25p < 0.0001p < 0.0001<br>m/z +26n.s.p = 0.0249<br>m/z +27n.s.n.s.
Fig. 4K	Palmitic acid<br><br>1-way ANOVA - Dunnett's multiple comparison test<br><br>KO1/CTLKO2/CTL<br>m/z 0p = 0.0012p = 0.0020<br>m/z +1p = 0.0028p = 0.0188<br>m/z +2p < 0.0001p < 0.0001<br>m/z +3p < 0.0001p < 0.0001<br>m/z +4p < 0.0001p < 0.0001<br>m/z +5p = 0.0007p = 0.0002<br>m/z +6p < 0.0001p < 0.0001<br>m/z +7p = 0.0006p = 0.0002<br>m/z +8p < 0.0001p < 0.0001<br>m/z +9n.s.n.s.<br>m/z +10p < 0.0001p < 0.0001<br>m/z +11p < 0.0001p < 0.0001<br>m/z +12p < 0.0001p < 0.0001<br>m/z +13p < 0.0001p < 0.0001<br>m/z +14p < 0.0001p < 0.0001<br>m/z +15p = 0.0003p = 0.0003<br>m/z +16p < 0.0001p < 0.0001
Fig. EV4H	Palmitic acid<br><br>two-tailed Student t-test<br><br>KO1/CTL<br>m/z 0p = 0.0070<br>m/z +1p = 0.0328<br>m/z +2n.s.<br>m/z +3n.s.<br>m/z +4p = 0.0171<br>m/z +5n.s.<br>m/z +6p = 0.0055<br>m/z +7n.s.<br>m/z +8p = 0.0008<br>m/z +9n.s.<br>m/z +10p = 0.0016<br>m/z +11n.s.<br>m/z +12p = 0.0032<br>m/z +13n.s.<br>m/z +14p = 0.0025<br>m/z +15n.s.<br>m/z +16p = 0.0052
