# Supplementary material for: THEM6‐mediated reprogramming of lipid metabolism supports treatment resistance in prostate cancer
Source: EMBO Mol Med. 2022 Jan 11;14(3):e14764. doi: 10.15252/emmm.202114764 (PMC8899912; doi:10.15252/emmm.202114764)
Supplement: Supplementary file 2 — Expanded View Figures PDF [file EMMM-14-e14764-s007.pdf]

# Expanded View Figures

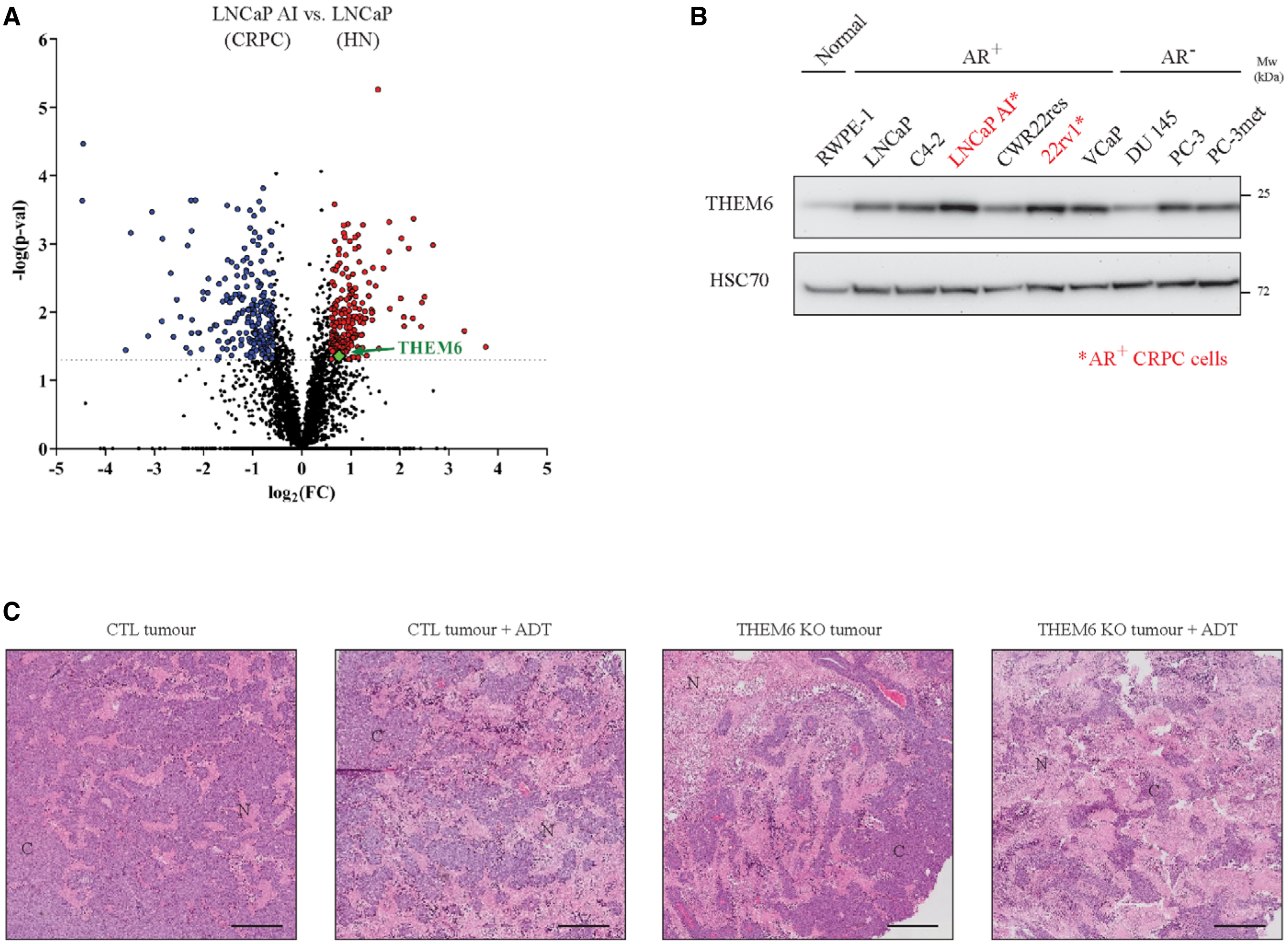


**Figure EV1. THEM6 is overexpressed following ADT resistance.**

- A Volcano plot of the differentially modulated proteins in LNCaP AI (CRPC) versus LNCaP (HN) tumours. Red and blue dots represent the proteins that are significantly up- and down-regulated, respectively (*P*-value ≤ 0.05, FC = 1.5).
- B Western blot analysis of THEM6 expression in PCa cells. HSC70 was used as a sample loading control. Within a panel of PCa cell lines, androgen receptor (AR)-positive CRPC (namely 22rv1 and LNCaP AI) cells express THEM6 at higher levels than their respective isogenic HN (LNCaP and CWR22res) counterparts.
- C Representative pictures of haematoxylin/eosin staining on orthografts from CWRres CTL and THEM6 KO tumours. C = cancer cells; N = necrotic area. Scale bar represents 1,000 µm.

Data information: Data reproducibility: (A) *n* = 3 tumours per group. (B) representative image from three independent biological experiments. (C) representative image from three tumours per group.
Source data are available online for this figure.


© 2022 The Authors

A

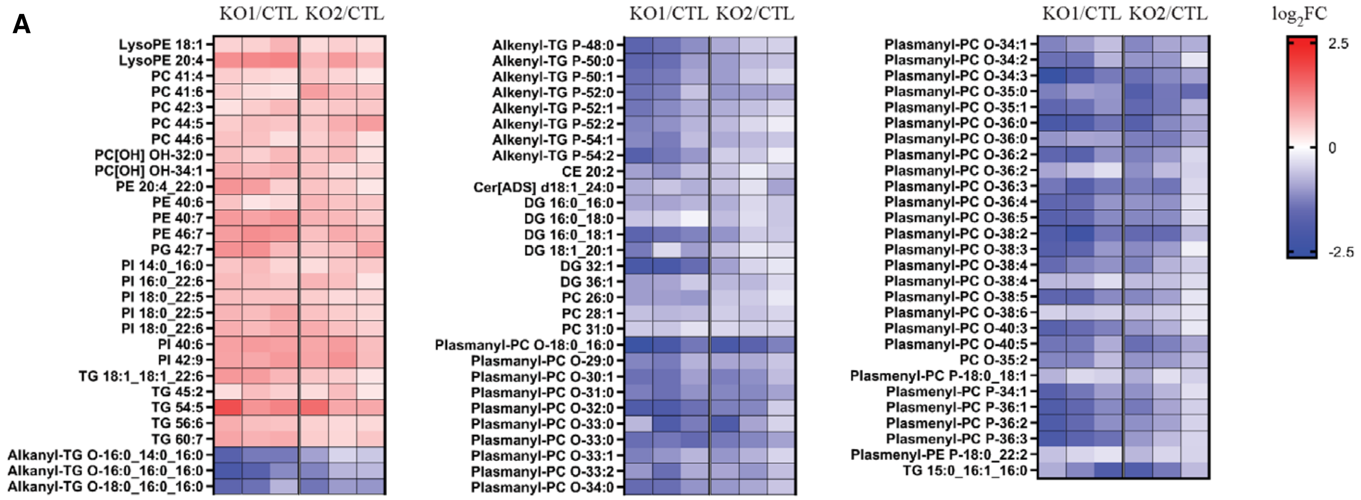


B

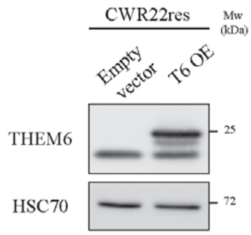


**Figure EV2. Loss of THEM6 alters lipid homeostasis.**

A Heatmap illustrating the steady-state levels of significantly regulated lipids in THEM6 KO LNCaP AI cells when compared to CTL ($P \leq 0.05$, FC = 1.3). Values are expressed as $log_2$(FC).

B Western blot analysis of THEM6 expression in CWR22res cells overexpressing THEM6 (T6 OE). HSC70 was used as a sample loading control.

Data information: Data reproducibility: (A) $n = 3$ independent biological experiments. (B) representative image from two independent biological experiments. Source data are available online for this figure.

© 2022 The Authors 

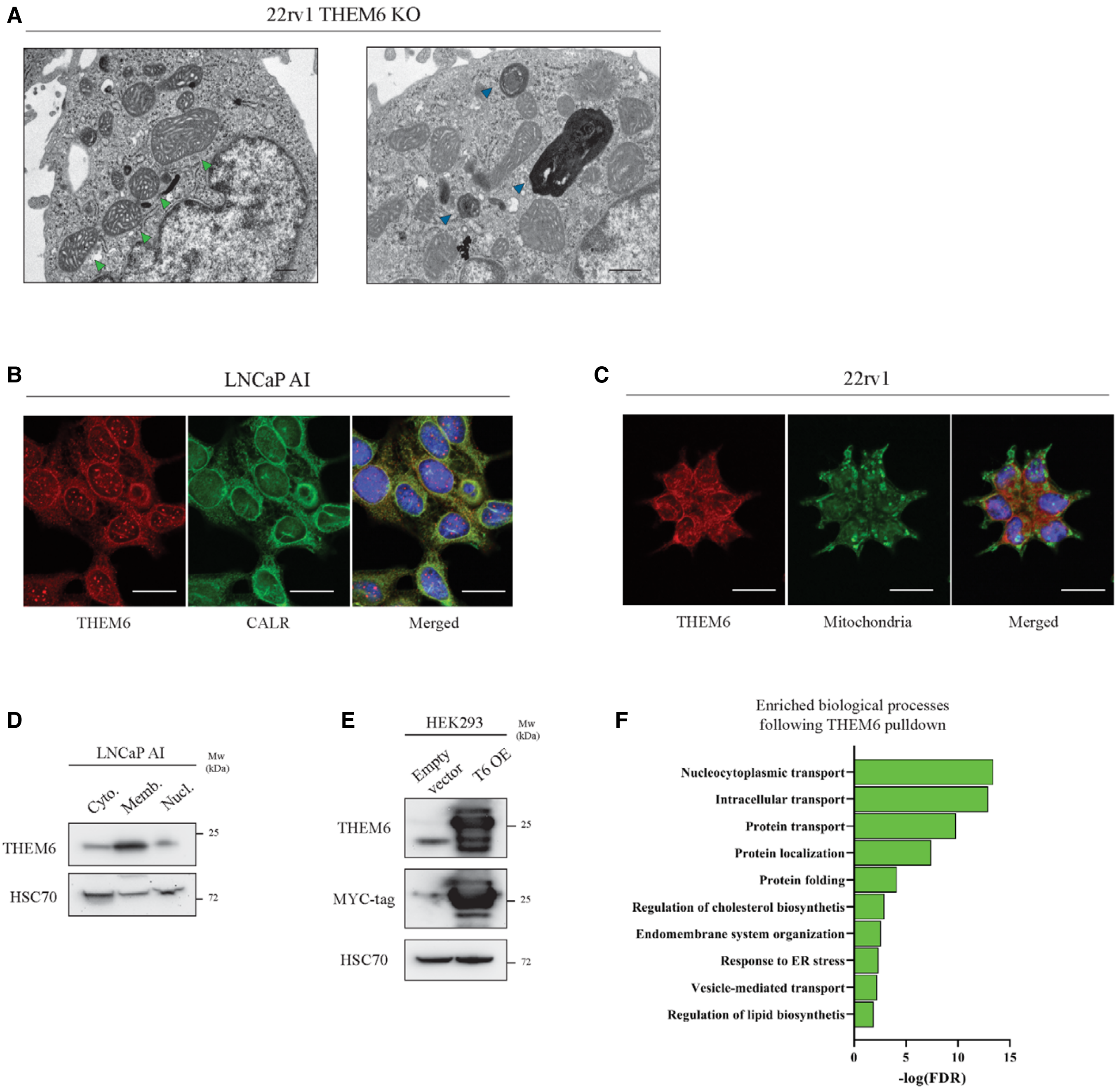


**Figure EV3. THEM6 is an ER membrane protein.**

- A Representative electron microscopy pictures of THEM6 KO 22rv1 cells. Green and blue arrows point towards enlarged mitochondria and abnormal lysosomal structure, respectively. Scale bar represents 500 nm.
- B Immunofluorescence showing co-localisation of THEM6 and the ER marker calreticulin in LNCaP AI cells. Scale bar represents 20 µm.
- C Immunofluorescence showing distinct localisations for THEM6 and mitochondria in 22rv1 cells. Scale bar represents 20 µm.
- D Western blot analysis of THEM6 expression in cytoplasmic (cyto.), membrane/organelle (memb.) and nuclear fractions (nucl.) of LNCaP AI cells. HSC70 was used as cytoplasmic-enriched marker.
- E Western blot analysis of THEM6 and MYC-tag expression in HEK293 cells overexpressing a MYC-tagged version of THEM6 (T6 OE). HSC70 was used as a sample loading control.
- F Enriched biological processes in THEM6-interacting proteins following THEM6 pulldown in T6 OE HEK293 cells.

Data information: Panel (F) Enrichment analysis was performed using the STRING database (http://string-db.org). Data reproducibility: (B, C, D) representative image from 3 independent biological experiments. (E) representative image from two independent biological experiments.
Source data are available online for this figure.

 © 2022 The Authors

**Figure EV4. Loss of THEM6 affects *de novo* sterol and FA synthesis.**

A Total pool of desmosterol in CTL and THEM6 KO 22rv1 cells. Data extracted from Fig 4D and E.
B Total pool of cholesterol in CTL and THEM6 KO CWR22res cells. Data extracted from Fig 4F.
C Total pool of cholesterol in CTL and THEM6 KO MCF-7 cells. Data extracted from Fig 4G.
D Pearson's correlation analysis of *HMGCS1*, *HMGCR*, *MVK*, *IDI1*, *FDPS*, *FDFT1*, *SC5D* with *THEM6* using the PRAD TCGA dataset. Results were obtained using the GEPIA website http://gepia.cancer-pku.cn/.
E Western blot analysis of SREBP1 (precursor and mature forms) expression in CTL and THEM6 KO CRPC cells. HSC70 was used as a sample loading control.
F Total pool of palmitic, oleic and stearic acid in CTL and THEM6 KO 22rv1 cells. Data extracted from Fig 4J and K.
G Labelled palmitic, oleic and stearic acid fractions derived from $^{13}C$-glucose and $^{13}C$-glutamine in CTL and THEM6 KO MCF-7 cells after 72 h of incubation.
H Relative isotopologue distribution of palmitic acid in CTL and THEM6 KO MCF-7 cells after 72 h of incubation.
I Total pool of palmitic, oleic and stearic acid in CTL and THEM6 KO 22rv1 cells. Data extracted from (G, H).

Data information: Panels (A, B, C, F, G, H, I) Data are presented as mean values ± SD. Statistical analysis: (A, B, F) One-way ANOVA with a Dunnett's multiple comparisons test. (C, G, H, I) two-tailed Student *t*-test. Data reproducibility: (A, B, C, F, G, H, I) $n = 3$ independent wells from the same cell culture. (E) representative image from three independent biological experiments.
Source data are available online for this figure.

© 2022 The Authors

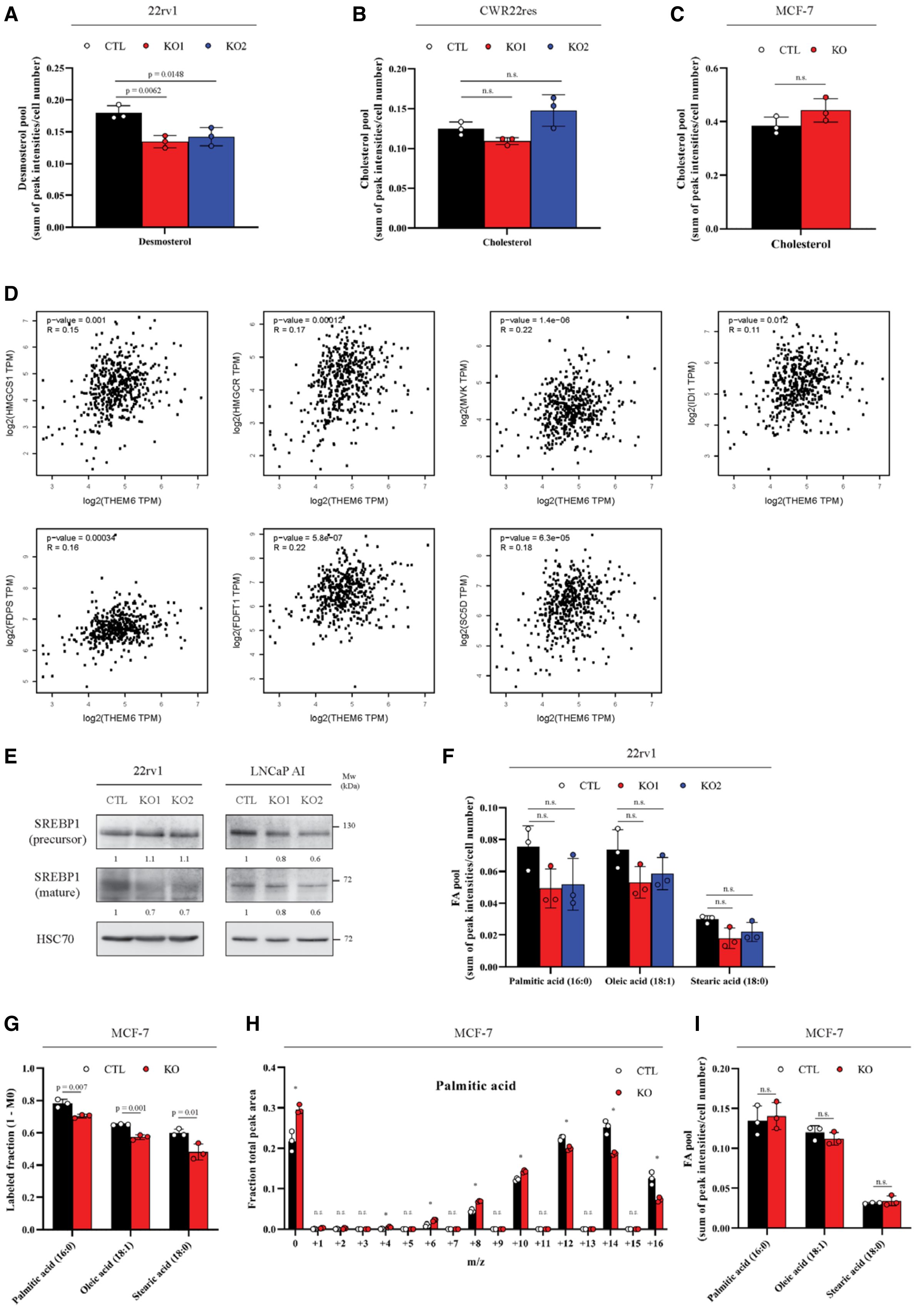


**Figure EV4.**

 © 2022 The Authors

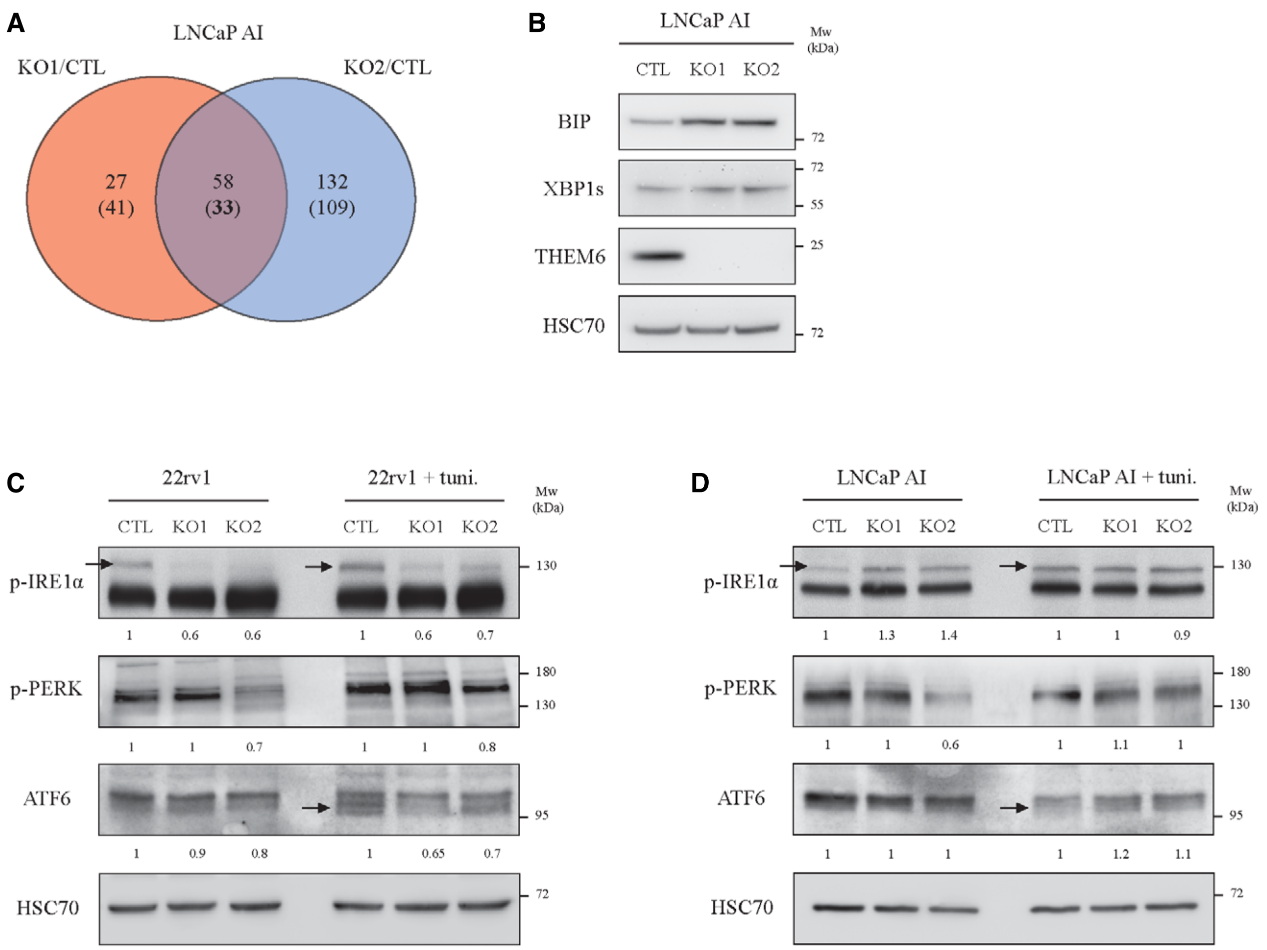


**Figure EV5. THEM6 regulates UPR activation in CRPC.**

A Venn diagram highlighting commonly modulated proteins (*P*-value ≤ 0.05, FC = 1.3) in THEM6 KO LNCaP AI cells (two clones) when compared to CTL. Up-regulated proteins are on top; Down-regulated proteins are into brackets.
B Western blot analysis of BIP, XBP1s and THEM6 expression in CTL and THEM6 KO LNCaP AI cells.
C Western blot analysis of p-IRE1α, p-PERK and ATF6 expression in CTL and THEM6 KO 22rv1 cells treated or not with tunicamycin.
D Western blot analysis of p-IRE1α, p-PERK and ATF6 expression in CTL and THEM6 KO LNCaP AI cells treated or not with tunicamycin.

Data information: Panels (B, C, D) HSC70 was used as a sample loading control. Data reproducibility: (B, C, D) representative image from three independent biological experiments.
Source data are available online for this figure.

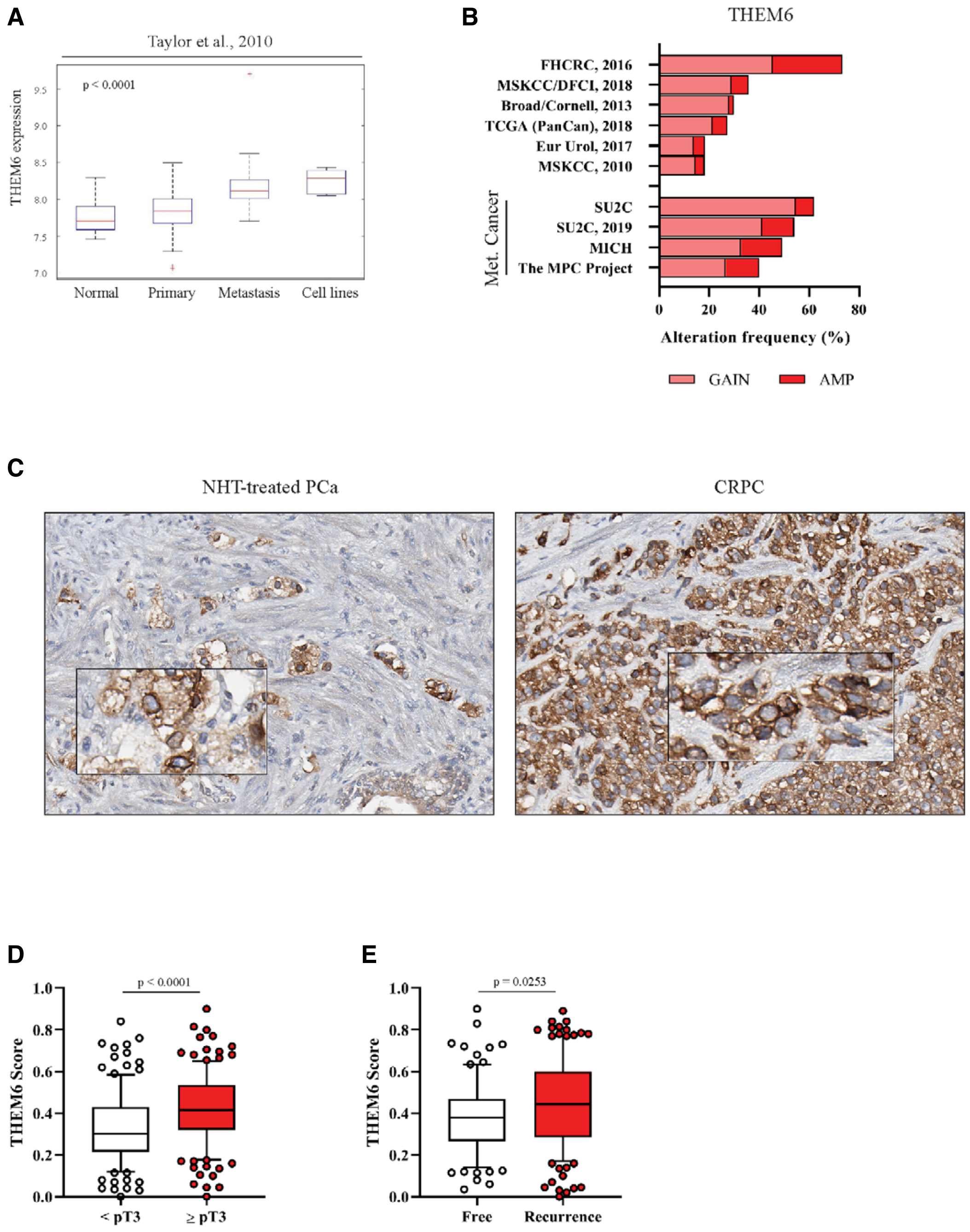


**Figure EV6. THEM6 is a clinically relevant target in CRPC.**

A Gene expression analysis of THEM6/c8orf55 in normal prostate tissues, primary tumours, distant metastases and PCa cell lines according to the GSE21034 dataset ($n = 179$).

B Percentage of PCa patients showing genomic alteration (copy number gain or amplification) for *THEM6* in publicly available datasets of PCa. Analysis was performed using the cbioportal website https://www.cbioportal.org/.

C High magnification pictures of THEM6 staining in NHT-treated and CRPC tumours.

D, E Quantification of THEM6 expression in PCa tissue samples according to T-stage (D) and cancer recurrence (E).

Data information: Panel (A) Center line corresponds to median of data, top and bottom of box correspond to 75$^{th}$ and 25$^{th}$ percentile, respectively. Whiskers extend to adjacent values (minimum and maximum data points not considered outliers). Panels (D, E) Center line corresponds to median of data, top and bottom of box correspond to 90$^{th}$ and 10$^{th}$ percentile, respectively. Whiskers extend to adjacent values (minimum and maximum data points not considered outliers). Statistical analysis: (A) pairwise ANOVA. (D, E) two-tailed Mann–Whitney *U*-test. Data reproducibility: (D) $n = 129$ tumours for T stage score $< 3$ and $n = 135$ tumours for T stage score $\geq 3$. (E) $n = 91$ for disease-free patients and $n = 136$ for recurred patients.
Source data are available online for this figure.

 © 2022 The Authors
