# Supplementary material for: THEM6‐mediated reprogramming of lipid metabolism supports treatment resistance in prostate cancer
Source: EMBO Mol Med. 2022 Jan 11;14(3):e14764. doi: 10.15252/emmm.202114764 (PMC8899912; doi:10.15252/emmm.202114764)
Supplement: Supplementary file 8 — Table EV6 [file EMMM-14-e14764-s008.docx]

**Table EV6:** List of primers used in this study.

| **Target Gene** | **Forward** | **Reverse** |
| --- | --- | --- |
| *hTHEM6* | ggagacaccaggctactaggac | tttccccagctgtaaggtga |
| *mTHEM6* | caagcaggccagagtagtca | cccactgtccctgagtaagc |
| *MVD* | gaccagggaaggggtcac | gcacttggtggtttcctga |
| *FDPS* | gagcggattctgcttttagg | gaagacccccacagatctca |
| *DHCR7* | aaaggggctttcatgtcgtt | cagactccaggcagagcac |
