# Supplementary material for: THEM6‐mediated reprogramming of lipid metabolism supports treatment resistance in prostate cancer
Source: EMBO Mol Med. 2022 Jan 11;14(3):e14764. doi: 10.15252/emmm.202114764 (PMC8899912; doi:10.15252/emmm.202114764)
Supplement: Supplementary file 9 — Table EV7 [file EMMM-14-e14764-s014.docx]

| **Target** | **Species** | **Company** | **Reference** | **Dilution** |
| --- | --- | --- | --- | --- |
| THEM6 | Rabbit | abcam | ab121743 | 1/1000 |
| VCL | Rabbit | Cell Signaling Technology | #13901 | 1/1000 |
| HSC70 | Mouse | Santa Cruz | sc-7298 | 1/1000 |
| CALR | Mouse | abcam | ab22683 | 1/1000 |
| Mitochondria | Mouse | Millipore | MAB1273 | 1/200 |
| SP1 | Rabbit | Cell Signaling Technology | #9389 | 1/1000 |
| AMFR | Rabbit | Cell Signaling Technology | #9590 | 1/1000 |
| SEC61b | Rabbit | Cell Signaling Technology | #14648 | 1/1000 |
| XPO1 | Rabbit | Cell Signaling Technology | #42649 | 1/1000 |
| MYC-tag | Mouse | Cell Signaling Technology | #2276 | 1/1000 |
| BiP | Rabbit | Cell Signaling Technology | #3177 | 1/1000 |
| XBP1s | Rabbit | Cell Signaling Technology | #40435 | 1/1000 |
| CHOP | Mouse | Cell Signaling Technology | #2895 | 1/500 |
| ATF4 | Rabbit | Cell Signaling Technology | #11815 | 1/500 |
| CALX | Rabbit | Cell Signaling Technology | #2679 | 1/1000 |
| p-PERK (T982) | Rabbit | abcam | ab192591 | 1/1000 |
| p-IRE1α (S724) | Rabbit | abcam | ab124945 | 1/1000 |
| ATF6 | Rabbit | Cell Signaling Technology | #65880 | 1/1000 |
| SREBP1 | Mouse | BD Bioscience | 557036 | 1/1000 |

**Table EV7:** List of antibodies used in this study.
