# Supplementary material for: THEM6‐mediated reprogramming of lipid metabolism supports treatment resistance in prostate cancer
Source: EMBO Mol Med. 2022 Jan 11;14(3):e14764. doi: 10.15252/emmm.202114764 (PMC8899912; doi:10.15252/emmm.202114764)
Supplement: Supplementary file 17 [file EMMM-14-e14764-s015.pdf]

EMBO Molecular Medicine

THEM6-mediated reprogramming of lipid metabolism supports treatment resistance in prostate cancer

Arnaud Blomme, Coralie Peter, Ernest Mui, Giovanny Rodriguez Blanco, Ning An, Louise Mason, Lauren Jamieson, Grace McGregor, Sergio Lilla, Chara Ntala, Rachana Patel, Marc Thiry, Sonia Kung, Marine Leclercq, Catriona Ford, Linda Rushworth, David McGarry, Susan Mason, Peter Repiscak, Colin Nixon, Mark Salji, Elke Markert, Gillian MacKay, Jurre Kamphorst, Duncan Graham, Karen Faulds, Ladan Fazli, Martin Gleave, Edward Avezov, Joanne Edwards, Huabing Yin, David Sumpton, Karen Blyth, Pierre Close, Daniel Murphy, Sara Zanivan, and Hing Leung
DOI: 10.15252/emmm.202114764

Corresponding authors: Hing Leung (h.leung@beatson.gla.ac.uk), Arnaud Blomme (a.blomme@uliege.be)

Review Timeline:

Submission Date:	23rd Jun 21
Editorial Decision:	9th Jul 21
Revision Received:	15th Nov 21
Editorial Decision:	26th Nov 21
Revision Received:	13th Dec 21
Accepted:	15th Dec 21

Editor: Lise Roth

Transaction Report:

(Note: With the exception of the correction of typographical or spelling errors that could be a source of ambiguity, letters and reports are not edited. Depending on transfer agreements, referee reports obtained elsewhere may or may not be included in this compilation. Referee reports are anonymous unless the Referee chooses to sign their reports.)

9th Jul 2021

Dear Prof. Leung,

Thank you for the submission of your manuscript to EMBO Molecular Medicine. We have now received feedback from the three reviewers who agreed to evaluate your manuscript. As you will see from the reports below, referees #1 and #2 acknowledge the interest of the study and are overall supporting publication of your work pending appropriate revisions. Referee #3 is more negative and raises concerns regarding the unclear clinical relevance of the study and the limited novelty in light of previous work on other members of the THEM family.

After cross-commenting with the referees, we would like to invite major revisions of the present manuscript, with the understanding that all referees' concerns should be addressed, in particular regarding controls, statistics and quantifications. The referees also agreed that the part related to other cancer types should be removed, as this seems to add confusion to the manuscript. The title should be modified to more adequately reflect the content of the manuscript.

Acceptance of the manuscript will entail a second round of review. EMBO Molecular Medicine encourages a single round of revision only and therefore, acceptance or rejection of the manuscript will depend on the completeness of your responses included in the next, final version of the manuscript. For this reason, and to save you from any frustrations in the end, I would strongly advise against returning an incomplete revision.

Revised manuscripts should be submitted within three months of a request for revision; they will otherwise be treated as new submissions, except under exceptional circumstances in which a short extension is obtained from the editor.

When submitting your revised manuscript, please carefully review the instructions that follow below. We perform an initial quality control of all revised manuscripts before re-review; failure to include requested items will delay the evaluation of your revision.

We require:

1) A .docx formatted version of the manuscript text (including legends for main figures, EV figures and tables). Please make sure that the changes are highlighted to be clearly visible.

2) Individual production quality figure files as .eps, .tif, .jpg (one file per figure). For guidance, download the 'Figure Guide PDF' (https://www.embopress.org/page/journal/17574684/authorguide#figureformat).

3) A .docx formatted letter INCLUDING the reviewers' reports and your detailed point-by-point responses to their comments. As part of the EMBO Press transparent editorial process, the point-by-point response is part of the Review Process File (RPF), which will be published alongside your paper.

4) A complete author checklist, which you can download from our author guidelines (https://www.embopress.org/page/journal/17574684/authorguide#submissionofrevisions). Please insert information in the checklist that is also reflected in the manuscript. The completed author checklist will also be part of the RPF.

5) It is mandatory to include a 'Data Availability' section after the Materials and Methods. Before submitting your revision, primary datasets produced in this study need to be deposited in an appropriate public database, and the accession numbers and database listed under 'Data Availability'. Please remember to provide a reviewer password if the datasets are not yet public (see https://www.embopress.org/page/journal/17574684/authorguide#dataavailability).

In case you have no data that requires deposition in a public database, please state so in this section. Note that the Data Availability Section is restricted to new primary data that are part of this study.

6) For data quantification: please specify the name of the statistical test used to generate error bars and P values, the number (n) of independent experiments (specify technical or biological replicates) underlying each data point and the test used to calculate p-values in each figure legend. The figure legends should contain a basic description of n, P and the test applied. Graphs must include a description of the bars and the error bars (s.d., s.e.m.).

7) We would also encourage you to include the source data for figure panels that show essential data. Numerical data should be provided as individual .xls or .csv files (including a tab describing the data). For blots or microscopy, uncropped images should be submitted (using a zip archive if multiple images need to be supplied for one panel). Additional information on source data and instruction on how to label the files are available at
.

8) Our journal encourages inclusion of *data citations in the reference list* to directly cite datasets that were re-used and obtained from public databases. Data citations in the article text are distinct from normal bibliographical citations and should directly link to the database records from which the data can be accessed. In the main text, data citations are formatted as follows: "Data ref: Smith et al, 2001" or "Data ref: NCBI Sequence Read Archive PRJNA342805, 2017". In the Reference list, data citations must be labeled with "[DATASET]". A data reference must provide the database name, accession number/identifiers and a resolvable link to the landing page from which the data can be accessed at the end of the reference. Further instructions are available at .

9) We replaced Supplementary Information with Expanded View (EV) Figures and Tables that are collapsible/expandable online. A maximum of 5 EV Figures can be typeset. EV Figures should be cited as 'Figure EV1, Figure EV2" etc... in the text and their respective legends should be included in the main text after the legends of regular figures.
- For the figures that you do NOT wish to display as Expanded View figures, they should be bundled together with their legends in a single PDF file called *Appendix*, which should start with a short Table of Content. Appendix figures should be referred to in the main text as: "Appendix Figure S1, Appendix Figure S2" etc.
- Additional Tables/Datasets should be labeled and referred to as Table EV1, Dataset EV1, etc. Legends have to be provided in a separate tab in case of .xls files. Alternatively, the legend can be supplied as a separate text file (README) and zipped together with the Table/Dataset file.

See detailed instructions here:
.

10) The paper explained: EMBO Molecular Medicine articles are accompanied by a summary of the articles to emphasize the major findings in the paper and their medical implications for the non-specialist reader. Please provide a draft summary of your article highlighting
- the medical issue you are addressing,
- the results obtained and
- their clinical impact.

This may be edited to ensure that readers understand the significance and context of the research. Please refer to any of our published articles for an example.

11) For more information: There is space at the end of each article to list relevant web links for further consultation by our readers. Could you identify some relevant ones and provide such information as well? Some examples are patient associations, relevant databases, OMIM/proteins/genes links, author's websites, etc...

12) Every published paper now includes a 'Synopsis' to further enhance discoverability. Synopses are displayed on the journal webpage and are freely accessible to all readers. They include a short stand first (maximum of 300 characters, including space) as well as 2-5 one-sentences bullet points that summarizes the paper. Please write the bullet points to summarize the key NEW findings. They should be designed to be complementary to the abstract - i.e. not repeat the same text. We encourage inclusion of key acronyms and quantitative information (maximum of 30 words / bullet point). Please use the passive voice. Please attach these in a separate file or send them by email, we will incorporate them accordingly.

Please also suggest a striking image or visual abstract to illustrate your article as a PNG file 550 px wide x 300-600 px high.

13) As part of the EMBO Publications transparent editorial process initiative (see our Editorial at http://embomolmed.embopress.org/content/2/9/329), EMBO Molecular Medicine will publish online a Review Process File (RPF) to accompany accepted manuscripts.
In the event of acceptance, this file will be published in conjunction with your paper and will include the anonymous referee reports, your point-by-point response and all pertinent correspondence relating to the manuscript. Let us know whether you agree with the publication of the RPF and as here, if you want to remove or not any figures from it prior to publication.
Please note that the Authors checklist will be published at the end of the RPF.

EMBO Molecular Medicine has a "scooping protection" policy, whereby similar findings that are published by others during review or revision are not a criterion for rejection. Should you decide to submit a revised version, I do ask that you get in touch after three months if you have not completed it, to update us on the status.

I look forward to receiving your revised manuscript.

With my best wishes,

Lise

Lise Roth, PhD
Editor
EMBO Molecular Medicine

***** Reviewer's comments *****

Referee #1 (Remarks for Author):

In this manuscript, Blomme and colleagues demonstrate that the endoplasmic reticulum resident thioesterase THEM6 regulates lipid homeostasis in prostate cancer and part mediates therapy resistance. The authors show that THEM6 expression in the ER is also tightly linked to ER homeostasis control.

This is a novel result that is very relevant to better understand (and possibly target) the role of lipid homeostasis in organelle biology, adaptive stress responses as well as their contribution to the selective advantage this could bring to tumor cells.

Major comments (limited to the 8 most important)

1) The authors should consider changing the title by indicating which thioesterase is involved and which cancer they study (see also point 6 below)
2) There are a couple of overstatements in the manuscript that should be modified. For instance it is not demonstrated that "THEM6 regulates membrane protein trafficking in the ER" but rather that ER components interact with THEM6 and see their expression altered in THEM6 ko cells.
3) Since there is a decrease in ER protein expression in THEM6 ko cells accompanied by morphological changes of this compartment, these changes should be quantified on EM pictures (size of the ER, ratio ER membrane over total membrane). The amount of ER proteins evaluated should then be corrected by the alterations observed at the ER level.
4) Is the overexpression of THEM6 observed in human tumors accompanied by a change in lipid content similar to that observed in cellular models. The authors use THEM6 ko models but what happens in overexpressing cells (likely to behave like PCa overexpressing tumor cells. The evaluation of lipid composition should as such be carried out in THEM6 overexpressing cells as well (or at least specific lipid species should be evaluated in THEM6 overexpressing cells). Since the sterol biosynthetic pathway appears to be also attenuated, and the ER affected in THEM6 ko cells, did the author evaluate the activation of SREBP1c?
5) The link to the UPR is interesting. How do the authors explain that the ATF4 pathway is the one altered ? Does that depend on PERK? If PERK activation cannot be completed in THEM6 ko cells, why then IRE1 activation could? It appears in Fig5 that there is an alteration of the basal expression of ER stress markers downstream of at least 2 branches (IRE1 and PERK), what happens under ER stress (for instance induced by tunicamycin)? This should be tested to be able to reach the authors conclusions. In addition, the stress response downstream of IRE1 leads to a different gene expression program depending whether the stress is proteotoxic or target lipid membrane composition. Even though this has been observed in yeast, the relevance of this observation could be relevant of the experimental system analyzed by the authors. The THEM6 ko appears to alter the expression of ATF6 target genes, and as such, as for SREBP1c, what about the activation of this arm of the UPR? To document the activation status of the UPR arms, the authors should test the activation of the 3 UPR proximal sensors PERK, IRE1 (through their phosphorylation status) and ATF6 (localization and proteolytic processing).
6) The data on the myc status in other cancers (osteosarcoma and triple negative breast cancer) introduces a lot of confusion to the take home message of the manuscript and could easily be removed, keeping the prostate cancer data and perhaps intersecting these data with those presented in Fig 1.
7) Since the authors have set up the raman analysis on their orthotopic tumors they could consider correlating the lipid content of human tumors (using frozen sections) with the expression of THEM6.
8) This manuscript shows that i) THEM6 overexpression in prostate tumors correlates with resistance to treatment and aggressiveness, ii) THEM6 controls cellular lipid content in cellular models, iii) THEM6 controls ER homeostasis and stress response, however the mechanistic links between these three events are currently missing in this manuscript. For instance, could it be possible to hypothesize that overexpression of THEM6, by altering ER lipid content could favor sustained activation of the UPR and therefore provide a selective advantage to the tumor cells? In which case those THEM6 overexpressing cells might be more sensitive to pharmacological inhibitors of the UPR which could then come as adjuvant to the standard of care.

Minor comments
1) The protein/gene annotation should be checked (CALX in text and legends vs CANX in figure 3)
2) Blots should be quantified and the number of experiments indicated
3) Molecular weight markers should be indicated on the blots

Referee #2 (Comments on Novelty/Model System for Author):

The data presented here are new, not overlapping with previous published work in the literature. The results are convincing, and the approaches used are accurate and adequate to the answers the authors want to get. The results are interesting in the cancer field and probably translatable in the future. Generally, I find the results could be relevant and valuable.

Referee #2 (Remarks for Author):

In this study, authors found that thioesterase superfamily member 6 (THEM6) is a marker of androgen-deprivation therapy resistance in advanced prostate cancer. In patients, THEM6 expression correlates with progressive disease and is associated with poor survival. THEM6 deletion diminishes in vivo tumour growth and restores castration sensitivity in orthograft models of castration-resistant prostate cancer. Authors also went in depth into associated mechanisms.
The data presented here are new, not overlapping with previous published work in the literature. The results are convincing, and the approaches used are accurate and adequate to the answers the authors want to get. The results are interesting in the cancer field and probably translatable in the future. Generally, I find the results could be relevant and valuable.

However, there are some questions that the authors should address before publication:

- Populations of patient´s used for analysis are not always clear. Please, PCas patient´s cohort should be detailed. Data curation is a critical step for using public databases. Paper will be improved if a summary table of clinical-pathological features of patients is included.
- Statistic methods should be carefully described. Multivariable Cox regression models should be derived to estimate the hazard ratio (HR) and mainly to adjusted for potential confounding factors: age, at least, should be included as a covariate.
- ¿Why only GSE21034 was included in the survival analysis? ¿What about GSE35988 and TCGA?

Minor changes:
The first time authors use PRAD abbreviation in the text, it should be explained (page 6).

Referee #3 (Comments on Novelty/Model System for Author):

The study by Blomme et al. focuses on the identification of thioesterases member 6 (THEM6) as a marker of androgen-deprivation therapy resistance and the development of castration-resistant prostate cancer. The key findings presented in the current study are that 1) THEM6 expression correlates with progressive disease in humans, 2) Deletion of THEM6 4educes tumor growth in vivo and restore castration sensitivity, 3)they show that THEM6 which is an ER membrane -associated protein regulates intracellular ether lipids which is necessary for the induction of ER stress. 4) loss of THEM5 alters lipid-mediated activation of ATF4. 5) THEM6 is frequently co-expressed with Myc and is necessary for the growth of Myc-driven tumors. Based on these findings they suggest that THEM6 is a novel marker of resistance to prostate cancer therapy.

The strengths of the study are:

1-The survival data in figure 1H and proliferation in fig. 1J is convincing.

2- The efficacy of KO1 and 2 on THEM6 expression appears the be very high (Fig. 1K).

3- The effect of THEM6 on lipid profiles in figure 2 is convincing.

Overall the study provides new role of THEM6 in ER stress and lipid metabolism.

However, several weaknesses were also noted:

4- The novelty of the findings is limited by the fact that ER stress has been strongly associated with Myc and resistance to therapy in prostate cancer (refs. 8 to 11). Other members of the THEM family have been shown to regulate ER stress via regulation of lipid biosynthesis (19, 20).
The reference to their previous study ref. 21 is missing the journal and volume.

5- In figure 1B: the increase between hormone naïve tumors (CWR22) and CRPC (22v1) is not impressive since the loading suggests that there are more proteins in the 22vr1 samples. Quantification is needed and why are not all tumors run in the same

gels? i.e what is the relative expression of THEM6 in CWE22 relative to LNCap?

6- The staining in Fig. 1D is too strong to distinguished cytosolic from ER-membrane-associated.

7- The choice of CWR22 tumors for the THEM6KO xenografts in Fig. 1n instead of LNCapAI tumors appears as an odd choice since THEM6 was selected based on the differential expression of THEM6 between HN and CRPC tumors. Likewise why the effect of THEM6 on cell growth in panel L done on LNCaP AI cells but not in panel N. Further the tumor volumes in panel M are much smaller (200-350mm) compared to the hormone naïve tumors in N (200-1100mm), yet the effect of KO of THEM6 seems more efficient at restraining growth in the HN tumors compare to CRPC in panel M.
Therefore, the conclusion that loss of THEM6 affects CRCP tumor growth (title of this section of the text) does not seem well justified.

8- The choice of HEK293 cells for the MS analysis is unfortunate since unrelated to prostate cancer.

9- The data presented in figure 4 is interesting but needs confirmation in another cell line.

10- The data related to Myc expression, ATF4 and other cancer types in figure 6 comes across as tagged on and the detracts from the main focus of the paper on CRPC.

Overall, while the study presents novel aspects of THEM6 in ER stress and lipid biosynthesis, considering that many controls are missing and the translation of these findings in the clinic is unclear and the impact on castration resistant prostate cancer specifically is lost as the story unfolds.

Referee #3 (Remarks for Author):

I do not suggest publication in EMM as a short report.

Referee #1 (Remarks for Author):

In this manuscript, Blomme and colleagues demonstrate that the endoplasmic reticulum resident thioesterase THEM6 regulates lipid homeostasis in prostate cancer and part mediates therapy resistance. The authors show that THEM6 expression in the ER is also tightly linked to ER homeostasis control.

This is a novel result that is very relevant to better understand (and possibly target) the role of lipid homeostasis in organelle biology, adaptive stress responses as well as their contribution to the selective advantage this could bring to tumor cells.

We thank Reviewer 1 for finding our story of interest and for the pertinent suggestions. We have now performed additional experiments to address the different comments and we feel that data from these experiments have significantly improved our manuscript.

Major comments (limited to the 8 most important)

1) The authors should consider changing the title by indicating which thioesterase is involved and which cancer they study (see also point 6 below)

6) The data on the myc status in other cancers (osteosarcoma and triple negative breast cancer) introduces a lot of confusion to the take home message of the manuscript and could easily be removed, keeping the prostate cancer data and perhaps intersecting these data with those presented in Fig 1.

We thank Reviewer 1 for these very sound comments. Following Reviewer 1's advices (also shared by Reviewer 3), we have decided to re-focus our paper on the role of THEM6 in CRPC. As suggested, we have now modified our title and removed the distracting data on MYC-driven sarcoma and breast cancer from the current version of the manuscript. Data describing the clinical significance of THEM6 in PCa patients are now presented separately in ***Figure 6****. Moreover, we have improved this section by including clinical correlations between THEM6 expression level and UPR activation in patients (see point 8).*

2) There are a couple of overstatements in the manuscript that should be modified. For instance it is not demonstrated that "THEM6 regulates membrane protein trafficking in the ER" but rather that ER components interact with THEM6 and see their expression altered in THEM6 ko cells.

3) Since there is a decrease in ER protein expression in THEM6 ko cells accompanied by morphological changes of this compartment, these changes should be quantified on EM pictures (size of the ER, ratio ER membrane over total membrane). The amount of ER proteins evaluated should then be corrected by the alterations observed at the ER level.

We thank the Reviewer for these comments. We have now carefully edited our text to avoid potential overstatements. Moreover, as suggested by Reviewer 1, we have quantified the lengths of the ER membrane and of the plasma membrane in CTL and THEM6-depleted cells. We found that loss of THEM6 decreased the length of the ER membrane but not of the plasma membrane. Consequently, the ratio ER/plasma membrane length was also decreased in THEM6 KO cells, supporting the idea that THEM6 loss has a strong impact on the ER membrane composition. Results of this experiment are now presented in ***Figure 3d****.*

4) Is the overexpression of THEM6 observed in human tumors accompanied by a change in lipid content similar to that observed in cellular models. The authors use THEM6 ko models but what happens in overexpressing cells (likely to behave like PCa overexpressing tumor cells. The evaluation of lipid composition should as such be carried out in THEM6 overexpressing cells as well (or at least specific lipid species should be evaluated in THEM6 overexpressing cells). Since the sterol

biosynthetic pathway appears to be also attenuated, and the ER affected in THEM6 ko cells, did the author evaluate the activation of SREBP1c?

*We thank the Reviewer for this sound comment and suggestion. We have now performed an additional lipidomic analysis on THEM6-overexpressing CWR22res cells. Results of this experiment are depicted in **Figure 2d-e**. In line with our KO data, we found that multiple TG, ether TG and ether PC molecules were specifically enriched in THEM6-overexpressing cells when compared to controls. Moreover, while THEM6 overexpression led to increased amounts of several lipid classes, the most significant changes were also observed for these three classes. Together, these results confirm our previous findings and highlight the importance of THEM6 in maintaining cellular lipid homeostasis.*

*Furthermore, we agree with Reviewer 1 that the activation of SREBP1, which is initiated in the ER, might be impaired in THEM6 KO cells. This alteration could possibly explain the decrease in de novo lipid synthesis observed in these cells. In agreement with this idea, we found that SREBP1 levels (precursor and processed forms) were moderately decreased in THEM6-deficient cells. We have now added this information in **Figure EV4e**.*

5) The link to the UPR is interesting. How do the authors explain that the ATF4 pathway is the one altered ? Does that depend on PERK? If PERK activation cannot be completed in THEM6 ko cells, why then IRE1 activation could? It appears in Fig5 that there is an alteration of the basal expression of ER stress markers downstream of at least 2 branches (IRE1 and PERK), what happens under ER stress (for instance induced by tunicamycin)? This should be tested to be able to reach the authors conclusions. In addition, the stress response downstream of IRE1 leads to a different gene expression program depending whether the stress is proteotoxic or target lipid membrane composition. Even though this has been observed in yeast, the relevance of this observation could be relevant of the experimental system analyzed by the authors. The THEM6 ko appears to alter the expression of ATF6 target genes, and as such, as for SREBP1c, what about the activation of this arm of the UPR? To document the activation status of the UPR arms, the authors should test the activation of the 3 UPR proximal sensors PERK, IRE1 (through their phosphorylation status) and ATF6 (localization and proteolytic processing).

*We thank the Reviewer for this pertinent question. From our initial proteomic analysis, performed on 22rv1 cells, we found that both the PERK and the IRE1α branches of the UPR were strongly affected by THEM6 KO (Figure 5a-b-c). In contrast with these data, we found that only ATF4 and ATF4 target genes, but not IRE1α signalling, were significantly down-regulated in absence of THEM6 in the LNCaP AI model (Figure 5e-f and Figure EV5b). This suggested to us that the ATF4 signalling was the main signalling pathway affected by THEM6 loss. Indeed, we confirmed that low ATF4 expression was a consistent feature of stable THEM6 KO cells, as well as following transient siRNA transfection. Moreover, this result was observed in all 9 cell lines tested (including prostate, breast and U2OS cells in the original manuscript). As suggested by Reviewer 1, we have now assessed the phosphorylation status of PERK and IRE1α, and the proteolytic processing of ATF6 in both 22rv1 and LNCaP AI cells using Western Blot analysis. Surprisingly, we found that activation of IRE1α and ATF6 was <u>impaired</u> following tunicamycin treatment in THEM6-depleted <u>22rv1 cells;</u> but we could not observe consistent changes in PERK phosphorylation (which is supposed to be upstream of ATF4) (**Figure EV5c**). Moreover, loss of THEM6 did not affect the tunicamycin-induced activation of PERK, IRE1α nor ATF6 <u>in the LNCaP AI model</u> (**Figure EV5d**). While the molecular mechanisms underlying THEM6-mediated control of ATF4 activation remain to be fully uncovered, we demonstrate that ATF4 activation can be rescued by increasing levels of ether lipids in THEM6 KO cells (hexadecylglycerol treatment, Figure 5j). This suggests that the decrease in ATF4 levels depends, at least in part, on the changes in lipid composition resulting from THEM6 KO. Interestingly, we noticed that THEM6 depletion often led to a decrease in ATF4 expression that was independent from changes in the phosphorylation status of PERK or eiF2α in several cell lines (data not shown). Therefore, we think that THEM6 loss might affect ATF4 expression in a non-canonical manner. Several metabolic factors*

such as mTORC1 (Ben-Sahra, Science, 2016; Park, Cell reports, 2017) or eiF2δ (Vasudevan, Nat. Commun., 2020) have been reported to regulate the activation of ATF4 independently of the PERK-eiF2α axis. While we are planning to test this challenging hypothesis in the future, we feel that the experiments required to answer that question are beyond the scope of the current paper.

7) Since the authors have set up the raman analysis on their orthotopic tumors they could consider correlating the lipid content of human tumors (using frozen sections) with the expression of THEM6.

We thank the Reviewer for this suggestion. Indeed, using Raman spectroscopy to routinely characterise lipid content in clinical biopsies is a protocol that we are currently implementing and that we wish to develop further in the future. However, at the moment, the results obtained from patient material (high heterogeneity, variability and complexity) are too premature to be included in the current study.

8) This manuscript shows that i) THEM6 overexpression in prostate tumors correlates with resistance to treatment and aggressiveness, ii) THEM6 controls cellular lipid content in cellular models, iii) THEM6 controls ER homeostasis and stress response, however the mechanistic links between these three events are currently missing in this manuscript. For instance, could it be possible to hypothesize that overexpression of THEM6, by altering ER lipid content could favor sustained activation of the UPR and therefore provide a selective advantage to the tumor cells? In which case those THEM6 overexpressing cells might be more sensitive to pharmacological inhibitors of the UPR which could then come as adjuvant to the standard of care.

*We would like to particularly thank the Reviewer for raising this important point. Indeed, our hypothesis is that, by regulating lipid homeostasis (especially controlling the levels of ether lipids), the ER-membrane associated protein THEM6 is required for the correct activation of the UPR, especially under conditions of lipid-mediated stress (as evidenced in Figure 5i-j). In the context of treatment-resistant PCa, we believe that THEM6 overexpression will allow cancer cells to cope better with the therapy-induced ER stress by facilitating UPR activation. To support this hypothesis, we have now assessed the enrichment of a specific UPR-gene signature (Adamson et al., Cell, 2016) in PCa patients that were separated according to high and low THEM6 expression. We found that patients with high THEM6 expression displayed a strong positive enrichment of the UPR-gene signature, and that the majority of the UPR-related genes were individually up-regulated in high THEM6 tumours. We have now included these data in **Figure 6m-n** and discussed this point in the Discussion section.*

Minor comments

1) The protein/gene annotation should be checked (CALX in text and legends vs CANX in figure 3)

We have now checked our manuscript and figures for consistency.

2) Blots should be quantified and the number of experiments indicated

We have now quantified Western Blots when required. The number of experiments is indicated in the Figure Legends.

3) Molecular weight markers should be indicated on the blots

Molecular weight marker has been added on the different panels.

Referee #2 (Comments on Novelty/Model System for Author):

In this study, authors found that thioesterase superfamily member 6 (THEM6) is a marker of androgen-deprivation therapy resistance in advanced prostate cancer. In patients, THEM6 expression correlates with progressive disease and is associated with poor survival. THEM6 deletion diminishes

in vivo tumour growth and restores castration sensitivity in orthograft models of castration-resistant prostate cancer. Authors also went in depth into associated mechanisms.

The data presented here are new, not overlapping with previous published work in the literature. The results are convincing, and the approaches used are accurate and adequate to the answers the authors want to get. The results are interesting in the cancer field and probably translatable in the future. Generally, I find the results could be relevant and valuable.

We thank the Reviewer for finding our results of interest and for the positive comments. We have now updated our manuscript to address Reviewer 2's questions.

However, there are some questions that the authors should address before publication:

- Populations of patient´s used for analysis are not always clear. Please, PCas patient´s cohort should be detailed. Data curation is a critical step for using public databases. Paper will be improved if a summary table of clinical-pathological features of patients is included.

We thank the Reviewer for this comment and apologize for the lack of information. We have now uploaded the supplementary tables recapitulating the clinico-pathological features of the two patient cohorts that were used in this study (Vancouver cohort and Glasgow cohort).

- Statistic methods should be carefully described. Multivariable Cox regression models should be derived to estimate the hazard ratio (HR) and mainly to adjusted for potential confounding factors: age, at least, should be included as a covariate.

We thank Reviewer 2 for raising this point. In addition to figure legends, we have now updated the materials and methods section to include the different statistical analysis performed on the patient samples (1-way ANOVA to test for THEM6 expression in the different patient subtypes and logrank test for the survival analysis). As suggested by Reviewer 2, we have also performed a multivariable Cox regression analysis on the patient cohort that was used for the survival analysis.

Our survival dataset (Glasgow cohort) comprised only 69 patients that were stained for THEM6 expression, and included 7 other variables (age, differentiation status, Gleason score, PSA level at diagnostic, PSA level at relapse, presence or absence of perineural invasion and presence or absence of lymphovascular invasion). Only three of these variables (age, perineural invasion and lymphovascular invasion) were consistently annotated for all the patients stained for THEM6 expression:

Clinico-Pathological Characteristics	Patient Numbers n (%) – Total = 69
Differentiation<br>Well / Moderate / Poor	8 (17.8) / 18 (40.0) / 19 (42.2)
Age<br><70 / >=70	27 (39.1) / 42 (60.9)
Gleason<br><=7 / >7	36 (61.0) / 23 (39.0)
Diagnostic PSA<br><10 / 10-20 / >20	17 (30.9) / 12 (21.8) / 26 (47.3)
Relapse PSA<br><10 / 10-20 / >20	30 (81.1) / 0 (0.0) / 7 (18.9)
Perineural Invasion<br>No / Yes	53 (76.8) / 16 (23.2)
Lymphovascular Invasion<br>No / Yes	64 (92.8) / 5 (7.2)

Therefore we performed the Cox regression analysis including these 4 covariates.

Importantly, the multivariate Cox regression analysis confirmed that THEM6 expression was significantly associated with overall survival (HR: 1.874, p-value: 0.041) and cancer-specific survival (HR: 3.084, p-value: 0.005) in PCa patients. As expected, perineural invasion signifies aggressive disease and presence of perineural invasion significantly correlates with overall patient survival. Moreover, association of THEM6 expression with recurrence-free survival was also close to reach significance (HR: 1.901, p-value: 0.067).

Overall survival

Variables in the Equation

	B	SE	Wald	df	Sig.	Exp(B)
THEM6_cyto (Binned)	.628	.307	4.190	1	.041	1.874
Age Code	.580	.318	3.330	1	.068	1.787
Perineural Invasion	-.959	.446	4.627	1	.031	.383
Lymphovascular Invasion	.608	.539	1.273	1	.259	1.836

Cancer-specific survival

Variables in the Equation

	B	SE	Wald	df	Sig.	Exp(B)
THEM6_cyto (Binned)	1.126	.399	7.965	1	.005	3.084
Age Code	.739	.392	3.541	1	.060	2.093
Perineural Invasion	-.992	.547	3.294	1	.070	.371
Lymphovascular Invasion	1.026	.559	3.364	1	.067	2.790

Time to relapse

Variables in the Equation

	B	SE	Wald	df	Sig.	Exp(B)
THEM6_cyto (Binned)	.642	.351	3.355	1	.067	1.901
Age Code	.589	.360	2.671	1	.102	1.802
Perineural Invasion	.424	.384	1.215	1	.270	1.528
Lymphovascular Invasion	2.526	.673	14.095	1	<.001	12.505

- Why only GSE21034 was included in the survival analysis? What about GSE35988 and TCGA?

*We thank the Reviewer for raising this point. We have now included progression-free survival data comparing high THEM6 vs. low THEM6 PCa patients using the PRAD TCGA dataset (performed via www.cbioportal.org). In line with the results obtained from other datasets, we found that high THEM6 expression was associated with shorter progression-free survival in PCa patients. Results of this analysis are now displayed in **Figure 6c**. Moreover, to our knowledge, the GSE35988 dataset does not include detailed survival patient data, and therefore was not used for survival analysis.*

Minor changes:

The first time authors use PRAD abbreviation in the text, it should be explained (page 6).

This has now been corrected.

Referee #3 (Comments on Novelty/Model System for Author):

The study by Blomme et al. focuses on the identification of thioesterases member 6 (THEM6) as a marker of androgen-deprivation therapy resistance and the development of castration-resistant prostate cancer. The key findings presented in the current study are that 1) THEM6 expression correlates with progressive disease in humans, 2) Deletion of THEM6 4educes tumor growth in vivo and restore castration sensitivity, 3)they show that THEM6 which is an ER membrane -associated protein regulates intracellular ether lipids which is necessary for the induction of ER stress. 4) loss of THEM5 alters lipid-mediated activation of ATF4. 5) THEM6 is frequently co-expressed with Myc and is necessary for the growth of Myc-driven tumors. Based on these findings they suggest that THEM6 is a novel marker of resistance to prostate cancer therapy.

The strengths of the study are:

1-The survival data in figure 1H and proliferation in fig. 1J is convincing.

2- The efficacy of KO1 and 2 on THEM6 expression appears the be very high (Fig. 1K).

3- The effect of THEM6 on lipid profiles in figure 2 is convincing.

Overall the study provides new role of THEM6 in ER stress and lipid metabolism.

We thank the Reviewer for the critical evaluation of our manuscript. Following remarks from Reviewer 3 (also shared by Reviewer 1), we have significantly modified the current manuscript to focus on the role of THEM6 in CRPC. We have also performed additional experiments to answer the Reviewer's concerns and we feel that the results from these experiments have significantly improved our manuscript.

However, several weaknesses were also noted:

4- The novelty of the findings is limited by the fact that ER stress has been strongly associated with Myc and resistance to therapy in prostate cancer (refs. 8 to 11). Other members of the THEM family have been shown to regulate ER stress via regulation of lipid biosynthesis (19, 20).

We thank the Reviewer for this comment and would like to convince the Reviewer of the highly novel and impactful nature of our findings. We would like to emphasize that our story is the first to directly assess the biological function of THEM6 and to demonstrate its importance in cancer biology, including tumour response to treatment. Indeed, while a previous report from Kume et al. (Kume et al., Mol. Cell. Proteomics, 2014) showed evidence of THEM6 overexpression in cancerous lesions when compared to normal tissues (using proteomics and immunohistochemistry), the biological function of THEM6 has not yet been studied. Our report describes for the first time the functional role of THEM6 in the regulation of lipid metabolism and ER biology in PCa and it uncovers the importance of THEM6 in the establishment of ADT resistance in PCa. Furthermore, these conclusions are supported by convincing clinical correlations.

We appreciate Reviewer 3's comments on earlier publications suggesting that THEM1 and THEM2 are able to regulate ER stress. However, we would strongly argue that these findings add to the novelty of our report, further strengthening the validity of our results along with their clinical implications. Indeed, we identified a role for THEM6 in regulating lipid metabolism and ER stress using a series of unbiased omics approaches. The fact that other members from the THEM superfamily could affect similar pathways, in different physiological contexts, suggests that we might have uncovered a conserved biological function of the THEM proteins. Importantly, THEM6 shares very little sequence homology with the other THEM (52% with THEM1, 24% with THEM2, 9% with THEM4 and THEM5). In addition, THEM6 is the only THEM family member displaying an N-terminal transmembrane domain. In fact, the Hotdog domain, which confers the thioesterase activity to the members of the THEM superfamily, is the only structural feature that is conserved across the different THEM proteins. Therefore, while their substrates might be different, the observation that

THEM1, THEM2 and THEM6 are independently able to regulate UPR activation suggests that this regulation depends on their shared function in lipid metabolism and, most importantly, relies on their enzymatic activity. This hypothesis is of particular interest for the development of small molecule inhibitors aiming at targeting THEM's enzymatic activity. The function of THEM1 and THEM2 has mainly been described in the context of metabolic diseases. As such, our report broadens the scope of the current literature and further supports the idea of targeting THEM thioesterase activity in different pathophysiological conditions.

We believe that the discovery of the role of THEM6 in lipid metabolism and ER stress regulation was crucial to understand the functional relevance of THEM6 overexpression in treatment-resistant PCa. Hence, our findings add to the literature on existing links between ER stress and therapy resistance in PCa by providing new insight into THEM6 and castration-resistant PCa. Along the same lines, the fact that high levels of UPR activation are a clinically relevant feature of therapy resistance in PCa enhances the translational potential of our findings. THEM6 could be used 1) as a biomarker, to highlight patients that could potentially benefit from UPR inhibition or/and 2) as a potential drug target, to develop adjuvant therapy to ADT (targeting THEM6 enzymatic activity). We have now discussed this point in our Discussion.

-The reference to their previous study ref. 21 is missing the journal and volume.

We thank the Reviewer for this remark. Our previous study was not yet published when we submitted the current manuscript. We have now updated the correct reference.

5- In figure 1B: the increase between hormone naïve tumors (CWR22) and CRPC (22v1) is not impressive since the loading suggests that there are more proteins in the 22vr1 samples. Quantification is needed and why are not all tumors run in the same gels? i.e what is the relative expression of THEM6 in CWE22 relative to LNCap?

We thank Reviewer 3 for highlighting this point and apologize for the lack of clarity. We have now quantified the Western Blots displayed in Figure 1b and confirmed that THEM6 is over-expressed in ADT-resistant tumours (CRPC) when compared to hormone-naïve (HN) counterparts. In addition, we would like to stress that our initial proteomic analysis was designed to specifically identify proteins that are associated with long-term resistance to ADT. For this purpose, we used matched pairs (hormone-naïve and castration-resistant) of isogenic prostate cancer cell lines that were orthotopically implanted into the prostate of immunodeficient mice. From this analysis, we identified THEM6 as a protein that was consistently up-regulated following ADT in both models (22rv1 vs CWR22res and LNCaP AI vs LNCaP). Therefore, the primary goal of the Western Blots presented in Figure 1b was to validate the increase in THEM6 expression following ADT in each comparison, without necessarily comparing the levels of THEM6 between the different HN tumours. As such, we did not run all tumours on the same gel. Unfortunately, we did not have enough tumour material left to run additional Western Blots. However, we extracted the data from our initial SILAC-based proteomic analysis and compared the levels of THEM6 between the different conditions:

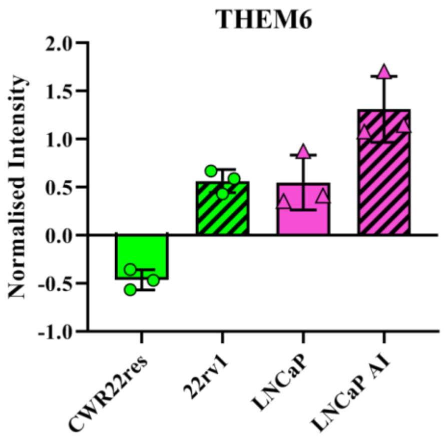

These results indicate that THEM6 is expressed at a higher level in LNCaP tumours than in CWR22res tumours. Moreover, this result is consistent with the expression level of THEM6 across different PCa cell lines which is displayed in ***Figure EV1b****.*

6- The staining in Fig. 1D is too strong to distinguished cytosolic from ER-membrane-associated.

*We agree with the Reviewer that the strong THEM6 staining in CRPC patients makes it difficult to discriminate between cytosolic and ER-membrane (perinuclear) staining. We have now provided additional high-magnification pictures showing ER-membrane (perinuclear) staining for THEM6 in patients (****Figure EV6c****). However, we would like to mention that total (cytosolic + membrane) THEM6 staining was used to quantify THEM6 expression in patient biopsies (****Figure 6f****).*

7- The choice of CWR22 tumors for the THEM6KO xenografts in Fig. 1n instead of LNCapAI tumors appears as an odd choice since THEM6 was selected based on the differential expression of THEM6 between HN and CRPC tumors. Likewise why the effect of THEM6 on cell growth in panel L done on LNCaP AI cells but not in panel N. Further the tumor volumes in panel M are much smaller (200-350mm) compared to the hormone naïve tumors in N (200-1100mm), yet the effect of KO of THEM6 seems more efficient at restraining growth in the HN tumors compare to CRPC in panel M.

Therefore, the conclusion that loss of THEM6 affects CRCP tumor growth (title of this section of the text) does not seem well justified.

We thank Reviewer 3 for the suggestion and again apologize for the lack of clarity. As suggested by the Reviewer, we have now evaluated the impact of THEM6 KO on the growth of the LNCaP AI tumours. Similar to the results obtained with the 22rv1 model, we show that THEM6 loss significantly impairs the growth of LNCaP AI tumours developed in castrated mice. Results of this experiment are shown in ***Figure 1h****. Furthermore, we agree that the use of the CWR22res model as a CRPC model might have been confusing. In fact, we used this model to complement the CRPC models (22rv1 and LNCaP AI) and to assess tumour sensitivity to acute ADT treatment. Accordingly, we observed that the growth of the THEM6-depleted CWR22res tumours was impaired following short term ADT in comparison to CTL tumours (Figure 1i, weeks 6-7). However, it is worth noting that THEM6 depletion did not affect tumour initiation nor tumour growth in the early timepoints of the experiment (Figure 1i, week 3). Therefore, these results suggest that THEM6 plays a more important role in tumour resistance to ADT than in tumour initiation. They also suggest that targeting THEM6 might be used as a potential strategy to increase PCa tumour response to ADT. We have now modified the Results section to clarify this point and to better describe the different models.*

8- The choice of HEK293 cells for the MS analysis is unfortunate since unrelated to prostate cancer.

*HEK-293 cells were initially selected because of their high transfection efficiency (**Figure EV3e**), in order to maximise the chances of identifying THEM6 interacting partners. While we agree that the experiment could have been performed on PCa cells, we have formally validated direct interactions between endogenous THEM6 and several hits from the proteomic analysis using immunoprecipitation experiments in both 22rv1 and LNCaP AI cells (**Figure 3i**).*

9- The data presented in figure 4 is interesting but needs confirmation in another cell line.

*We thank Reviewer 3 for this sound suggestion. Using RNA-Seq analysis of LNCaP AI cells, we now demonstrate that the genes involved in sterol biosynthesis are negatively enriched in THEM6 KO cells, a result that is similar to the 22rv1 model (**Figure 4a-c**). Moreover, to confirm that the negative impact of THEM6 loss on sterol and fatty acid (FA) synthesis is not cell type dependent, we performed additional sterol and FA tracing analyses using the highly steroidogenic and lipogenic human breast cancer MCF-7 cell line. For both experiments, we found that THEM6 KO significantly reduced the rates of de novo cholesterol and FA synthesis. Results of these experiments are now presented in **Figure 4g** and **Figure EV4g-i**. Finally, we also demonstrate that THEM6 expression is strongly associated with several genes involved in cholesterol and FA synthesis in PCa patients (Figure 4h-i), suggesting that the link between THEM6 and sterol biosynthesis might be conserved in PCa patients.*

10- The data related to Myc expression, ATF4 and other cancer types in figure 6 comes across as tagged on and the detracts from the main focus of the paper on CRPC.

Overall, while the study presents novel aspects of THEM6 in ER stress and lipid biosynthesis, considering that many controls are missing and the translation of these findings in the clinic is unclear and the impact on castration resistant prostate cancer specifically is lost as the story unfolds.

*We thank the Reviewer for highlighting this critical issue. Following Reviewer 3's (and Reviewer 1's) comments, we have now removed the distracting data presented in Figure 6 (MYC-inducible and triple negative breast cancer models). Moreover, in addition to the role of THEM6 in the regulation of CRPC-associated lipid metabolism, we now demonstrate that high THEM6 expression in PCa patients is associated with a sustained activation of the UPR (**Figure 6m-n**). Therefore, we believe that THEM6 could be used as a biomarker to identify PCa patient subgroups that could potentially benefit from pharmacological inhibition of the UPR. Moreover, we think that further research is warranted to explore THEM6 as a potential therapeutic target. For example, small molecule inhibitors aiming at targeting THEM6 enzymatic activity could be used as adjuvant therapy to sensitise CRPC patients to ADT.*

26th Nov 2021

Dear Prof. Leung,

Thank you for the submission of your revised manuscript to EMBO Molecular Medicine.
We have received the enclosed reports from the referees who had originally reviewed your manuscript, and as you will see, they are now fully supportive of publication. I am therefore very pleased to inform you that we will be able to accept your manuscript once the following minor editorial points will be addressed:

1/ Main manuscript text:
- Thank you for addressing the queries from the data editors. Please accept the changes and remove the red text.
- Thank you for providing the antibodies details, please also indicate the dilutions used.
- Data availability section: Please note that the datasets must be public before acceptance of the manuscript. Only new generated datasets must be listed in this section, you may therefore remove the list of already published databases that were used in the study.

2/ Figures and Appendix:
- We usually accommodate a maximum of 5 EV figures. Would you consider making one of your EV figures a main figure?
- Please make sure that all exact p values are indicated in the figures or in their legends, including for n.s. (non significant).

3/ Thank you for providing a synopsis text. Please also suggest a striking image or visual abstract to illustrate your article as a PNG/TIFF/JPEG file 550 px wide x 300-600 px high.

4/ As part of the EMBO Publications transparent editorial process initiative (see our Editorial at http://embomolmed.embopress.org/content/2/9/329), EMBO Molecular Medicine will publish online a Review Process File (RPF) to accompany accepted manuscripts.
This file will be published in conjunction with your paper and will include the anonymous referee reports, your point-by-point response and all pertinent correspondence relating to the manuscript. Let us know whether you agree with the publication of the RPF and as here, if you want to remove or not any figures from it prior to publication.
Please note that the Authors checklist will be published at the end of the RPF.

I look forward to receiving your revised manuscript.

With kind regards,

Lise Roth

Lise Roth, PhD
Editor
EMBO Molecular Medicine

****** Reviewer's comments *****
Referee #1 (Remarks for Author):

I would like to thank the authors for the efforts made to address my comments raised on the initial version of the manuscript. Results are now very clear and robust, the conclusions are supported by the data, these conclusions are novel and identify THEM6 as a major actor in therapy resistance in prostate cancer. At last the flow of the manuscript has now reached maturity in the revised version. I strongly recommend publication of this mansucript in Embo Molecular Medicine.

Referee #2 (Remarks for Author):

Authors have answered all previous concerns. Manuscript has been improved and I don´t have additional comments.

Referee #3 (Comments on Novelty/Model System for Author):

The revised version now includes valuation of key data in other cell lines and justification of the novelty of the findings relative to what was already published for other THEM members.

Referee #3 (Remarks for Author):

The revised version has addressed all of this reviewer previous concerns.

The authors performed the requested editorial changes.

15th Dec 2021

Dear Prof. Leung,

Thank you for submitting the revised files. I am very pleased to inform you that your manuscript is now accepted for publication and will be sent to our publisher to be included in the next available issue of EMBO Molecular Medicine!

Please read below for additional important information regarding your article, its publication and the production process.

Congratulations on your interesting work!

With my best wishes,

Lise Roth

Lise Roth, Ph.D
Editor
EMBO Molecular Medicine

EMBO PRESS

YOU MUST COMPLETE ALL CELLS WITH A PINK BACKGROUND ↓

PLEASE NOTE THAT THIS CHECKLIST WILL BE PUBLISHED ALONGSIDE YOUR PAPER

Corresponding Author Name: Arnaud Blomme/Hing Leung
Journal Submitted to: EMBO Molecular Medicine
Manuscript Number: EMM-2021-14764

Reporting Checklist For Life Sciences Articles (Rev. June 2017)

This checklist is used to ensure good reporting standards and to improve the reproducibility of published results. These guidelines are consistent with the Principles and Guidelines for Reporting Preclinical Research issued by the NIH in 2014. Please follow the journal's authorship guidelines in preparing your manuscript.

A- Figures

1. Data

The data shown in figures should satisfy the following conditions:

- the data were obtained and processed according to the field's best practice and are presented to reflect the results of the experiments in an accurate and unbiased manner.
- figure panels include only data points, measurements or observations that can be compared to each other in a scientifically meaningful way.
- graphs include clearly labeled error bars for independent experiments and sample sizes. Unless justified, error bars should not be shown for technical replicates.
- if n< 5, the individual data points from each experiment should be plotted and any statistical test employed should be justified
- Source Data should be included to report the data underlying graphs. Please follow the guidelines set out in the author ship guidelines on Data Presentation.

2. Captions

Each figure caption should contain the following information, for each panel where they are relevant:

- a specification of the experimental system investigated (eg cell line, species name).
- the assay(s) and method(s) used to carry out the reported observations and measurements
- an explicit mention of the biological and chemical entity(ies) that are being measured.
- an explicit mention of the biological and chemical entity(ies) that are altered/varied/perturbed in a controlled manner.

- the exact sample size (n) for each experimental group/condition, given as a number, not a range;
- a description of the sample collection allowing the reader to understand whether the samples represent technical or biological replicates (including how many animals, litters, cultures, etc.).
- a statement of how many times the experiment shown was independently replicated in the laboratory.
- definitions of statistical methods and measures:
  - common tests, such as t-test (please specify whether paired vs. unpaired), simple χ2 tests, Wilcoxon and Mann-Whitney tests, can be unambiguously identified by name only, but more complex techniques should be described in the methods section;
  - are tests one-sided or two-sided?
  - are there adjustments for multiple comparisons?
  - exact statistical test results, e.g., P values = x but not P values < x;
  - definition of 'center values' as median or average;
  - definition of error bars as s.d. or s.e.m.

Any descriptions too long for the figure legend should be included in the methods section and/or with the source data.

In the pink boxes below, please ensure that the answers to the following questions are reported in the manuscript itself. Every question should be answered. If the question is not relevant to your research, please write NA (non applicable). We encourage you to include a specific subsection in the methods section for statistics, reagents, animal models and human subjects.

USEFUL LINKS FOR COMPLETING THIS FORM

http://www.antibodypedia.com
http://1degreebio.org
http://www.equator-network.org/reporting-guidelines/improving-bioscience-research-repor
http://grants.nih.gov/grants/olaw/olaw.htm
http://www.mrc.ac.uk/Ourresearch/Ethicsresearchguidance/Useofanimals/index.htm
http://ClinicalTrials.gov
http://www.consort-statement.org
http://www.consort-statement.org/checklists/view/32-consort/66-title
http://www.equator-network.org/reporting-guidelines/reporting-recommendations-for-tumo
http://datadryad.org
http://figshare.com
http://www.ncbi.nlm.nih.gov/gap
http://www.ebi.ac.uk/ega
http://biomodels.net/
http://biomodels.net/miriam/
http://jjj.biochem.sun.ac.za
https://osp.od.nih.gov/biosafety-biosecurity-and-emerging-biotechnology/
http://www.selectagents.gov/

B- Statistics and general methods

Please fill out these boxes ↓ (Do not worry if you cannot see all your text once you press return)

1.a. How was the sample size chosen to ensure adequate power to detect a pre-specified effect size?	At least three samples were used per experiment assessing any biological phenotype. This number is sufficient to support the statistical analyses performed in this manuscript.
1.b. For animal studies, include a statement about sample size estimate even if no statistical methods were used.	For mice experiment, a pilot study was performed to evaluate the take up rate of Pca tumours (22rv1 and CWR22res = 100%; LNCaP AI = 80%). Therefore the initial number of mice was determined to ensure at least 5 replicates per group and to allow to perform statistical analysis.
2. Describe inclusion/exclusion criteria if samples or animals were excluded from the analysis. Were the criteria pre-established?	Animals that did not develop LNCaP AI tumours (1 per group) were excluded from the analysis according to pre-established criteria.
3. Were any steps taken to minimize the effects of subjective bias when allocating animals/samples to treatment (e.g. randomization procedure)? If yes, please describe.	Mice were randomly allocated into experimental groups at the time of injection.
For animal studies, include a statement about randomization even if no randomization was used.	Mice were randomly allocated into experimental groups at the time of injection.
4.a. Were any steps taken to minimize the effects of subjective bias during group allocation or/and when assessing results (e.g. blinding of the investigator)? If yes please describe.	Mouse experiments always involved two investgators. One investigator was harvestng and counting the cells before handing the suspension to the second investigator, who carried the injection/surgery blindly (not knowing which condition was injected).
4.b. For animal studies, include a statement about blinding even if no blinding was done	Cell injections were performed blindly by the investigator (not knowing which cell suspension was prepared).
5. For every figure, are statistical tests justified as appropriate?	Statistical tests are justified in Figure Legends
Do the data meet the assumptions of the tests (e.g., normal distribution)? Describe any methods used to assess it.	Any assumptions or corrections (adjustment for multiple comparisons) have been mentioned in the Figure Legends. Non-parametric tests were used for the statistical analysis of mouse and human tumour comparisons.
Is there an estimate of variation within each group of data?	Data are presented as mean values +/- SD

Is the variance similar between the groups that are being statistically compared?	Yes

C- Reagents

6. To show that antibodies were profiled for use in the system under study (assay and species), provide a citation, catalog number and/or clone number, supplementary information or reference to an antibody validation profile. e.g., Antibodypedia (see link list at top right), 1DegreeBio (see link list at top right).	All antibodies used in this study were validated on the manufacturer's website. A detailed list of antibodies is provided as supplementary table.
7. Identify the source of cell lines and report if they were recently authenticated (e.g., by STR profiling) and tested for mycoplasma contamination.	CWR22Res cells (hormone-responsive variant of CWR22 cells) were obtained from Case Western Reserve University, Cleveland, Ohio. All other cell lines were obtained from ATCC. All cells were maintained at 37°C under 5% CO2 and routinely harvested with trypsin. All cell lines were authenticated by STR DNA profiling and were tested negative for mycoplasma using the Mycoalert mycoplasma detection kit (Lonza, Basel, Switzerland). Cells were kept in culture for a maximum of 10 passages after recovery from frozen vials.

* for all hyperlinks, please see the table at the top right of the document

D- Animal Models

8. Report species, strain, gender, age of animals and genetic modification status where applicable. Please detail housing and husbandry conditions and the source of animals.	10-weeks old CD1-nude male mice were used for xenograft and orthograft experiments. Mice were ordered from Charles River (UK), housed in randomised groups of five, at 19°C to 23°C with a 12-hour light-dark cycle, and were fed a conventional diet (Rat and Mouse Standard Expanded, B&K Universal, UK) with water ad libitum. They were housed in enriched environment, with igloos, cardboard tubes and chewing sticks.
9. For experiments involving live vertebrates, include a statement of compliance with ethical regulations and identify the committee(s) approving the experiments.	In vivo orthograft experiments were performed in accordance with the ARRIVE guidelines63, and approved by a local ethics committee (University of Glasgow) under the Project Licences P5EE22AEE and 70/8645 in full compliance with the UK Home Office regulations (UK Animals (Scientific Procedures) Act 1986) and EU directive.
10. We recommend consulting the ARRIVE guidelines (see link list at top right) (PLoS Biol. 8(6), e1000412, 2010) to ensure that other relevant aspects of animal studies are adequately reported. See author guidelines, under 'Reporting Guidelines'. See also: NIH (see link list at top right) and MRC (see link list at top right) recommendations. Please confirm compliance.	Our study complies with the ARRIVE guidelines.

E- Human Subjects

11. Identify the committee(s) approving the study protocol.	This study was approved by the West of Scotland Research Ethics Committee (05/S0704/94) and the Chair of the University of British Columbia Clinical Research Ethics Board (UBC CREB number: H09-01628).
12. Include a statement confirming that informed consent was obtained from all subjects and that the experiments conformed to the principles set out in the WMA Declaration of Helsinki and the Department of Health and Human Services Belmont Report.	All patients involved in this study provided written informed consent and all experiments conformed to the principles set out in the WMA Declaration of Helsinki and the Department of Health and Human Services Belmont Report.
13. For publication of patient photos, include a statement confirming that consent to publish was obtained.	NA
14. Report any restrictions on the availability (and/or on the use) of human data or samples.	NA
15. Report the clinical trial registration number (at ClinicalTrials.gov or equivalent), where applicable.	NA
16. For phase II and III randomized controlled trials, please refer to the CONSORT flow diagram (see link list at top right) and submit the CONSORT checklist (see link list at top right) with your submission. See author guidelines, under 'Reporting Guidelines'. Please confirm you have submitted this list.	NA
17. For tumor marker prognostic studies, we recommend that you follow the REMARK reporting guidelines (see link list at top right). See author guidelines, under 'Reporting Guidelines'. Please confirm you have followed these guidelines.	NA

F- Data Accessibility

18: Provide a "Data Availability" section at the end of the Materials & Methods, listing the accession codes for data generated in this study and deposited in a public database (e.g. RNA-Seq data: Gene Expression Omnibus GSE39462, Proteomics data: PRIDE PXD000208 etc.) Please refer to our author guidelines for 'Data Deposition'.<br><br>Data deposition in a public repository is mandatory for:<br>a. Protein, DNA and RNA sequences<br>b. Macromolecular structures<br>c. Crystallographic data for small molecules<br>d. Functional genomics data<br>e. Proteomics and molecular interactions	A Data Availability section is provided at the end of the Materials and Methods section: For proteomics, the raw files and the MaxQuant search results files have been deposited as partial submission to the ProteomeXchange Consortium via the PRIDE partner repository64. Data are available with identifiers PXD024407, PXD024433 and PXD024456. For lipidomics, the raw LC-MS/MS files and the processed data have been deposited on the Metabolomics Workbench repository. GSEA code is available on https://github.com/prepiscak/them6_gsea.<br>The following databases were used in this study:<br>The Cancer Genome Atlas (TCGA - https://tcga-data.nci.nih.gov/tcga/);<br>GSE35988 (https://www.ncbi.nlm.nih.gov/geo/query/acc.cgi?acc=GSE35988);<br>GSE21034 (https://www.ncbi.nlm.nih.gov/geo/query/acc.cgi?acc=GSE21034);<br>STRING v11.0 (https://string-db.org/cgi/input.pl).
19. Deposition is strongly recommended for any datasets that are central and integral to the study; please consider the journal's data policy. If no structured public repository exists for a given data type, we encourage the provision of datasets in the manuscript as a Supplementary Document (see author guidelines under 'Expanded View' or in unstructured repositories such as Dryad (see link list at top right) or Figshare (see link list at top right).	Proteomics and Lipidomics analysis have been deposited and referenced in the Data Availability section.
20. Access to human clinical and genomic datasets should be provided with as few restrictions as possible while respecting ethical obligations to the patients and relevant medical and legal issues. If practically possible and compatible with the individual consent agreement used in the study, such data should be deposited in one of the major public access-controlled repositories such as dbGAP (see link list at top right) or EGA (see link list at top right).	Genomic datasets used were available from www.cbioportal.org. Other publicly available datasets have been referenced in the Data Availability section.
21. Computational models that are central and integral to a study should be shared without restrictions and provided in a machine-readable form. The relevant accession numbers or links should be provided. When possible, standardized format (SBML, CellML) should be used instead of scripts (e.g. MATLAB). Authors are strongly encouraged to follow the MIRIAM guidelines (see link list at top right) and deposit their model in a public database such as Biomodels (see link list at top right) or JWS Online (see link list at top right). If computer source code is provided with the paper, it should be deposited in a public repository or included in supplementary information.	Source code have been provided.

G- Dual use research of concern

22. Could your study fall under dual use research restrictions? Please check biosecurity documents (see link list at top right) and list of select agents and toxins (APHIS/CDC) (see link list at top right). According to our biosecurity guidelines, provide a statement only if it could.	Our study does not fall under dual use research restrictions.
